# Supplementary material for: Meta-ethnography to understand healthcare professionals’ experience of treating adults with chronic non-malignant pain
Source: BMJ Open. 2017 Dec 21;7(12):e018411. doi: 10.1136/bmjopen-2017-018411 (PMC5778293; doi:10.1136/bmjopen-2017-018411)
Supplement: Supplementary file 1 [file bmjopen-2017-018411supp001.pdf]

Appendix 1: Concepts not fitting the conceptual analysis

1. **BARRY 2010: Logistical Factors - Ancillary Staff**: Physicians expressed concern that they had insufficient qualified staff to implement pain management.
2. **BARRY 2010: Logistical Factors - Insurance Coverage:** Some physicians expressed concerns about the logistics of insurance coverage for pain management services and the difficulty in characterizing patients' pain status because of restrictions from insurance companies.
3. **FONTANA 2008: critical analysis:** A conflict of interest in which the patients' best interests are given a low priority. Nurses did not see prescribing decisions as ethical ones and, as a result, did not recognize the conflicts that were at work when they made these decisions.
4. **HOLLOWAY 2009A: Initiating clinical care**: The ability to provide pain management for residents when needed varied considerably between facilities; for some it involved basic care such as emotional support, positioning and using hot-packs, whereas in some facilities, they administered pain medication and had responsibility for monitoring the effectiveness of the pain management interventions and documentation.
5. **HOLLOWAY 2009B: Perfect Positioning (Rewards of Getting It Right):** Assistants felt sustained and fulfilled by the rewarding aspects of caring. All spoke of their passion for, enjoyment of, and love for their work (and this is why they stayed in it). Despite the emotional distress associated with observing people in pain, assistants gained satisfaction from seeing residents relieved of pain. Discussed poor financial remuneration they received and expressed the view that it was emotional fulfilment that made the job worthwhile.
6. **KAASALAINEN 2010A: interactions with long-term care staff and managers:** Nurse Practitioner was viewed as a nurse with added skills who assisted other healthcare team members with managing uncontrolled pain and was often used as an additional resource for nurses.
7. **LIU 2014: Instigator implementing non-pharmacological interventions**: Skills in distraction, reassurance and being gentle. Nursing assistants explained how they distracted or reassured residents who were in pain.
8. **LOCKENHOFF 2013: Age Differences in Time Horizons (treatment planning):** Consistently reported that they planned and administered pain management regimens for the long run.
9. **LUNDH 2004: variation 1:** ''I can feel very curious! What do these symptoms stand for?''
10. **OOSTERHOF 2014: Experiences concerning the treatment outcome (Learning new behaviour):** HCPs recognised that behaviour change takes a lot of effort, and requires a combination of explanation and practice. Some patients managed to learn new behaviour

and implement it within their daily life because they have always been active or because of their good body awareness or physical preference. Other might find it difficult to keep up effort due to personal problems and poor social support.

11. **SCOTT-DEMPSTER 2015: ''It's not a One Trick Pony'':** Physiotherapists regarded activity pacing as part of the pain management tool box to bring about change. Activity pacing was not described as something that was clearly definable or had fixed parameters. Achieving this flexibility could be challenging, as it meant that the physiotherapist had to adapt activity pacing for each individual.
12. **SEAMARK 2013: Cost:** Some did not consider cost and prescribed what was needed. Others felt it was important to bear in mind.
13. **SIEDLECKI 2014: CORE CONCEPTS/ TAKING OWNERSHIP:** Some did not take ownership of the problem and saw it as someone else's problem.
14. **STINSON 2013: Barriers to Care (patient-specific barriers):** Difficult to maintain a consistent pain management regimen because of time commitments and reluctance of younger people with pain.
15. **STINSON 2013: Pain Management Strategies (support systems)**: HCPs recognised the importance of peer support for patients.
