# Supplementary material for: Meta-ethnography to understand healthcare professionals’ experience of treating adults with chronic non-malignant pain
Source: BMJ Open. 2017 Dec 21;7(12):e018411. doi: 10.1136/bmjopen-2017-018411 (PMC5778293; doi:10.1136/bmjopen-2017-018411)
Supplement: Author's manuscript [file bmjopen-2017-018411.draft_revisions.pdf]

BMJ Open is committed to open peer review. As part of this commitment we make the peer review history of every article we publish publicly available.

When an article is published we post the peer reviewers' comments and the authors' responses online. We also post the versions of the paper that were used during peer review. These are the versions that the peer review comments apply to.

The versions of the paper that follow are the versions that were submitted during the peer review process. They are not the versions of record or the final published versions. They should not be cited or distributed as the published version of this manuscript.

BMJ Open is an open access journal and the full, final, typeset and author-corrected version of record of the manuscript is available on our site with no access controls, subscription charges or pay-per-view fees (http://bmjopen.bmj.com).

If you have any questions on BMJ Open's open peer review process please email editorial.bmjopen@bmj.com

BMJ Open

A meta-ethnography to understand healthcare professionals' experience of treating adults with chronic non-malignant pain

Journal:	*BMJ Open*
Manuscript ID	bmjopen-2017-018411
Article Type:	Research
Date Submitted by the Author:	27-Jun-2017
Complete List of Authors:	Toye, Francine; Nuffield Orthopaedic Centre, Oxford, Nuffield Orthopaedic Centre<br>Seers, Kate; Royal College of Nursing Research Institute, Warwick Medical School, University of Warwick, RCN RI<br>Barker, Karen L.; University of Oxford, Nuffield Department of Orthopaedics, Rheumatology and Musculoskeletal Sciences
**Primary Subject Heading**:	Qualitative research
Secondary Subject Heading:	Patient-centred medicine
Keywords:	QUALITATIVE RESEARCH, PAIN MANAGEMENT, meta-ethnography, qualitative evidence synthesis, healthcare professionals

SCHOLARONE™
Manuscripts

A meta-ethnography to understand healthcare professionals' experience of treating adults with chronic non-malignant pain

Fran Toye (corresponding author) Nuffield Orthopaedic Centre, Oxford University Hospitals NHS Foundation Trust, Windmill Road, Oxford, OX3 7HE, UK; frantoye@hotmail.com; +44 1865 737526

Kate Seers, Royal College of Nursing Research Institute, Warwick Medical School, University of Warwick, Coventry, UK

Karen Barker, Nuffield Department of Orthopaedics, Rheumatology and Musculoskeletal Sciences (NDORMS), University of Oxford, Oxford, UK.

KEY WORDS

QUALITATIVE RESEARCH

PAIN MANAGEMENT

Meta-ethnography

Healthcare professionals

Word count 4708

For peer review only

Abstract

Objectives: We aimed to explore healthcare professionals (HCPs) experience of treating chronic non-malignant pain by conducting a conceptual synthesis of qualitative research. Understanding this experience from the perspective of HCPs can contribute to improvements in the provision of care.

Design: Synthesis of qualitative research using meta-ethnography. We searched five electronic bibliographic databases from inception to November 2016. We included studies that explore HCPs experience of treating adults with chronic non-malignant pain. We utilised the GRADE-CERQual framework to rate confidence in review findings.

Results: We screened the 954 abstracts and 184 full texts and included 77 published studies reporting the experiences of over 1551 international HCPs including doctors, nurses and allied health professionals. We abstracted 6 themes: (1) a sceptical cultural lens; (2) navigating juxtaposed models of medicine; (3) navigating patient-clinician borderland; (4) challenge of dual advocacy; (5) personal costs; (6) the craft of pain management. We rated confidence in review findings as moderate (5 findings) or high (1 finding).

Conclusions: This is the first internationally relevant synthesis of healthcare professionals' experiences of treating people with chronic non-malignant pain. We have presented a conceptual model that helps us to understand, think about and modify our experience of treating patients with chronic pain. Our findings highlight cultural scepticism that might explain why patients with chronic pain feel they are not believed. Findings highlight a potential dualism in the bio-psychosocial model and the complexity of navigating therapeutic relationships. Our conceptual model may be transferable to other patient groups or situations.

For peer review only

For peer review only - http://bmjopen.bmj.com/site/about/guidelines.xhtml

Strengths and limitations of this study

- The synthesis is drawn from a systematic review of 77 qualitative studies reporting the experience of treating patients with chronic non-malignant pain.
- Conceptual syntheses of qualitative research provide insight into complex processes of healthcare and provide tools that allow clinicians to reflect on this process.
- Although the GRADE-CERQual provides a useful framework for determining confidence in qualitative syntheses, there is currently no consensus on how to do this.

The original protocol

The original study protocol can be found at:
https://www.journalslibrary.nihr.ac.uk/programmes/hsdr/1419807/#/

A short film presenting the key themes can be found on NIHR YouTube (https://www.youtube.com/watch?v=477yTJPg10o)

Funding statement

National Institute for Health Research Health Services and Delivery Research Programme (14/198/07). The views and opinions expressed therein are those of the authors and do not necessarily reflect those of the HS&DR Programme, NIHR, NHS or the Department of Health.

Competing interests statement.

This study was funded by the National Institute for Health Research Health Services and Delivery Research Programme (14/198/07). KB and FT authored 2 of the 77 studies included in this review.

Data sharing statement

The original protocol, full monograph and supporting material can be found at: http://www.nets.nihr.ac.uk/projectsOld/hsdr/1419807

Contributor statement

FT, KB and KS made a substantial contribution to the design, acquisition, analysis and interpretation of data. FT drafted the first, subsequent and final versions. KS and KB revised all versions for important intellectual content and approved the final version. All authors agree to be accountable for the accuracy and integrity of the work

For peer review only - http://bmjopen.bmj.com/site/about/guidelines.xhtml

Background

Each year over five million people develop chronic pain [1]. This can be challenging to treat because it persists beyond healing time and is not easy to explain. We already know that patients with chronic non-malignant pain can experience healthcare as adversarial [2]. Understanding this experience from the perspective of healthcare professionals (HCPs) will contribute to improvements in care provision. Although there is a large body of qualitative research exploring HCPs experience of treating chronic non-malignant pain, there has been no attempt to systematically search for and integrate this knowledge. We aimed to explore HCPs experience by conducting a conceptual synthesis of qualitative research. The Cochrane Qualitative Research Methods Group acknowledges the importance of including qualitative findings within evidence based healthcare [3] and insights from several syntheses have contributed to a greater understanding of complex processes. For example, medicine taking [4], diabetes [5] antidepressants [6], osteoporosis [7]chronic musculoskeletal pain [2, 8] and chronic pelvic pain [9].

Methods

We used the methods of meta-ethnography [10] refined for larger studies [11]. There are various methods for synthesising qualitative research [12-16]. An important distinction is between (a) those that *describe* findings and (b) those, like meta-ethnography, that develop *conceptual* understandings [10]. There are seven stages to meta-ethnography: getting started, deciding what is relevant, determining how studies are related, translating studies into each other, synthesising translations and expressing the synthesis [11].

In their original text, Noblit and Hare do not advocate an exhaustive search [10] and the number of studies included in meta-ethnographies ranges [13, 15, 17]. We searched five electronic bibliographic databases (Medline, Embase, Cinahl, Psychinfo, Amed) using terms adapted from the InterTASC Information Specialists' Sub-Group (ISSG) Search Filter Resources [18-21]. We used subject headings and free text terms for *qualitative research*, combined with subject heading and free text terms for *pain* (figure 1). We did not include citation checks, hand searching, grey literature or PhDs. Previous experience has shown us that this does not necessarily add significant conceptual value [11]. We included studies that explored HCP's experience of treating adults with chronic non-malignant pain. We excluded: acute pain, head pain and arthritic conditions. XX and YY screened the titles, abstracts and full text of potential studies.

There is currently no consensus on what makes a qualitative study 'good' [17, 22]. However, a growing number of reviewers are appraising studies for qualitative synthesis [15]. We did not intend

For peer review only - http://bmjopen.bmj.com/site/about/guidelines.xhtml

to use rigid guidelines, but felt it important to seriously consider quality. We used three methods of appraisal: (a) The Critical Appraisal Skills Programme (CASP) questions for qualitative research [23]; (b) constructs from a qualitative study in a previous meta-ethnography [24]; (c) a global appraisal of whether the study was: 'key' (conceptually rich); 'satisfactory'; 'irrelevant'; or 'fatally flawed' [6, 22]. If XX and YY did not agree, they consulted ZZ for a final decision. We utilised the GRADE-CERQual framework [25] which aims to rate how much confidence readers can place in review findings. GRADE-CERQual suggest four domains: (1) '*Methodological limitations'*; (2) '*Relevance'* ; (3) '*Adequacy of data'* (the 'degree of richness and quantity of data supporting a review finding'); *(4)* '*Coherence'* (consistency across primary studies) [25]; and finally, an overall rating of confidence (high, moderate, low, very low).

We read studies in batches of topic or professional grouping and did not use an *index* paper to *orientate* the synthesis [26], as we felt that this choice can have a dramatic impact on the interpretation [27]. Two reviewers read each paper to identify, describe and list concepts. If they agreed that there was no clear concept then it was excluded. XX, YY and ZZ sorted the concepts into conceptual categories through constant comparison[10]. The three reviewers worked with a project advisory group of patient and professional members to finalise the themes that would be included in the final analytic stage ('synthesising translations') [10].

Findings

Figure 2 shows the number of hits, exclusions and inclusions from our search. We screened the abstracts of 954 studies and excluded 770. We retrieved 184 full texts and excluded 99 for the following reasons: not HCP experience [28-40], not qualitative [41-50], not chronic pain [51-95], out of scope [96-122], or limited qualitative data [123-126]. Of the 85 potential studies, we unanimously excluded six on the grounds of methodological report [127-132], and two as irrelevant [133, 134]. XX and YY unanimously appraised five studies as 'key papers' [40, 135-138] and 72 as 'satisfactory'. They did not agree about four studies [139-142] which were subsequently included by ZZ. We included 77 published studies reporting the experiences of over 1551 HCPs (table 1). HCPs included a diversity of doctors, nurses and allied health professionals in various contexts and geographical locations. Table 1 shows the author and year of publication, geographic context, number of participants, data collection method, analytic approach and sequence of analysis.

Two reviewers identified 371 concepts from 77 studies. Fifteen out of 371 concepts did not fit our conceptual analysis (appendix 1). There were several topics with insufficient weight to develop robust themes: ethnicity [143-147]; gender [148] and older people [149-157]. These may indicate

For peer review only - http://bmjopen.bmj.com/site/about/guidelines.xhtml

useful areas of further research. Experience specific to opioid prescribing is reported elsewhere. A short film presenting the key themes is available on YouTube [https://www.youtube.com/watch?v=477yTJPg10o].

XX, YY and ZZ organised the 371 concepts into 42 conceptual categories. They worked with a project advisory group of patient and professional members to further abstract these categories into six themes: (1) a sceptical cultural lens; (2) navigating juxtaposed models of medicine; (3) navigating patient-clinician borderland; (4) the challenge of dual advocacy; (5) personal cost; (6) the craft of pain management; These themes are illustrated below with narrative exemplars. Indicators of confidence in each review findings [25] are shown in table 2, which shows: the number of studies rated as key/valuable or satisfactory (*methodological limitations*); the number of concepts (*adequacy*); the number of studies out of 77 (*coherence*); an assessment of study relevance; our overall assessment of confidence. We rated our confidence in the review finding as high when it was supported by more than half of the studies. However, there is currently no agreed way of making an assessment of confidence for qualitative synthesis.

A sceptical cultural lens

This describes a cultural lens that provides a sceptical view of chronic non-malignant pain. Clinical work involves determining whether the pain is *something* or *nothing*. HCPs found themselves making non-clinical judgements about whether the pain was *real* or *imagined*, and pondered dissonance between what the patient said and what the HPC could see.

> Sometimes I could have a patient sitting there and saying that they are hurting, 10 out of 10, and they are sitting like you and I[158].
>
> Some people say "This is the worst pain I've had in my whole life" without any real sort of physical signs of pain so it's really tough; we have a complex job in assessing that[159].

There was a sense of being 'on guard' [160] against exploitation from fraudulent claims.

> It is not clear to me why he is the way he is... this catastrophic pain and what he is telling himself about it . . . but there is always a little bit . . . of concern; am I being manipulated, is this really real? [161]
>
> Such people . . . ones whose wishes you cannot fathom – provoke anger and frustration because at some point, you don't always know how to verify their complaints. You feel somewhat exploited. It is a very unpleasant feeling [162]

For peer review only - http://bmjopen.bmj.com/site/about/guidelines.xhtml

HCPs engaged in moral 'boundary work' in order to decipher patients claims. This boundary work hinged on a multiplicity of dualities superposed on a polarity of *'good'* and *'bad'* (easy/difficult; explained/not explained, local/diffuse pain; adherent/non-adherent; stoical/weak; motivated; unmotivated; accepting/resisting; non-complaining/complaining; deserving/non-deserving). Some recognised that this moral boundary work was flawed and advocated trust as the basis of the therapeutic relationship.

> Sometimes we say 'oh she came in with back pain but I don't think she's really in pain'. . . but really even if somebody is in pain and distress, [it] doesn't always have to be in how they present themselves . . . that doesn't mean she is not in pain [163].

> "I hate to say it . . . but I used to be one of the people that used to say, 'Oh, well, they are probably just wanting attention.' But I've changed in that matter. People are in pain, and it's not just to get attention." [164]

Navigating juxtaposed models of medicine

This describes the challenge of navigating juxtaposed models of medicine: the biomedical and the bio-psychosocial. There was a culturally entrenched pull towards the biomedical *siren song* of diagnosis[136].

> Being able to track something gives me more comfort than going by what you're telling me . . . because I like to see proof . . . You [want to] . . . be convinced that you're treating *something* and that what you're treating is real [165].

> I will listen to their story, I will examine them and I always say you have got to exclude the physical first that is your job . . . we have an obligation to exclude the physical first and not jump into [psychosocial explanations] because it reduces the patient to being an un-necessary complainer and I don't believe that they really are [137]

At times, chronic pain was seen to obscure 'real' health problems.

> They don't seem to worry about issues that might be real . . . like his cholesterol is high . . . there are some other issues that he needs to attend to . . . his father died when he was fifty two. He's not worried [158].

Some HPCs utilised a dualistic bio-psychosocial model, whereby once *something* biomedical has been excluded, they made an abrupt shift towards psychosocial explanations. Here clinical work

For peer review only - http://bmjopen.bmj.com/site/about/guidelines.xhtml

shifted away from diagnosis towards persuading patients that psychosocial factors influenced pain. This abrupt shift could threaten the therapeutic relationship.

> The terminology . . . psychiatric and psychological . . . have a stigma attached to them that is not intended . . . we accept that patients with long term pain will have a psychological component to it but actually labelling it as that [163]

HCPs tended to default to physical explanations or used ‘bridging’ strategies to conceal the shift.

> It is a subtlety and if you present [the explanation for pain] as a completely airy fairy psychological, it is up to you, then they are going to go away dissatisfied, so you have got to lead them in gently [137]

> If you start from the body and if you ask a little carefully how things work when you are physically like that, then it is not threatening, and you can approach things, like, through the body [138]

Some felt that a diagnosis could help a patient to move forward, or give a sense of relief. Some even ‘feign[ed] diagnostic certainty’ to achieve this goal [137].

> The fact that they tell you that you have a problem that’s not just to do with your nerves and that there’s something wrong physically . . . Just that gives you a certain sense of relief[166].

Other voiced ethical concerns about the deception of *feigning* diagnostic certainty.

> I think giving it a label that actually has no justification I think is misleading to the patient and I actually feel quite strongly about that [137]

Not all HCPs utilised a dualistic bio-psychosocial model and did not make this abrupt shift towards psychosocial explanations. Rather, they utilised an embodied bio-psychosocial approach with ‘no breaking point where the physical becomes psychological’[167]. There was a sense that pain is multidimensional and that the ‘physician gaze’ [168] multifocal. Here clinical work involved understanding person’s suffering from the outset of care.

> While we talked . . . many losses came up and I began myself to think about what all this was about in fact, what is this pain? Where it came out that there was a lot of disappointment, where there was divorce and . . . yes, it can’t be purely physiological [138]

For peer review only - http://bmjopen.bmj.com/site/about/guidelines.xhtml

> Once a person's life has fallen apart it's not so much about the pain and the injury anymore. It's about all these other things in their life and it's all these other things that need to be addressed in order to get them better and get them back on track [169].

Those who utilised an embodied psychosocial model recognised that sitting alongside and supporting patients, rather trying to 'fix' them, could be rewarding for both the patient and their HCP.

> See [each clinical encounter] as a journey rather than 'this is my one chance at this and I have to get it all done in one go' . . . then you both have a more realistic expectation of where things are going to take you. It's about moving away from that place of stuckness and creating a little bit of momentum . . . you know when you're pushing a car . . . [its] the first movement that is the hardest one, and then it builds [163].

> I think the sort of traditional model of treatment doesn't allow people to express how pain has affected their whole life, it is very homed in to the particular area of the body and trying to fix it, and I just find it more satisfying to work in a way that acknowledges and discusses the impact[170]

However, time restrictions encouraged HCPs to focus on the physical body and were perceived as a barrier to an embodied approach/

> We are limited by the amount of time with the patient. I know this sounds bad, but [talking about pain] opens a can of worms[171].

Navigating patient-clinician borderland

This describes the complexity of navigating the geography between patient and HPC. Tensions could arise when patient's expectations of diagnosis and treatment were not met.

> People feel let down by their doctors . . . The degree of satisfaction is very low . . . basically because we don't solve their problem . . . They go from one to the other, they find a doctor who gives them hope and they go to him [166].

HPCs made concessions in order to navigate the borderland. For example, they might make choices of doubtful medical utility, such as prescribing pain killers or referring for an investigation, in order to maintain relationships. Concessions were necessary to balance long and short term gains.

For peer review only - http://bmjopen.bmj.com/site/about/guidelines.xhtml

> Sometimes patients refuse to believe that their condition cannot be treated . . . and insistently ask for a series of medical investigations that you, as a doctor, would not perform. In such cases, a medical investigation can work as a therapy because it . . . shows that you listened to them [172].
>
> There definitely have been times when I've issued a [sick] note just purely because it's became so antagonistic in a consultation that's what I've done [173].

HCPs also described the challenge of balancing professional expertise and patient empowerment, and could find it difficult to stand back and let patients make a *'wrong'* decision.

> Trying to allow myself to listen objectively and to . . . sit with the fact that actually [the patient] might want to do something which is wholly *unsensible*, but allowing that to happen if that truly is what they want [170].

> I recognise that . . . we are trying to promote learning by giving choice and allowing people to get it wrong . . . we learn by *doing* not by being told what to do. I get that, although it is still hard . . . not to give advice when I see . . . that the advice can be really helpful[174]

HCPs wanted patients to be empowered, but felt on guard against being 'bullied' into unhelpful clinical decisions

> You want to have a team effort with the patient but you also don't want to be bullied. . . . On days when you are feeling a bit under par they can be the toughest patients to deal with [160].

When there was conflict, the 'short-circuit' [175] was to take control. However, there remained a sense that discussion rather than enforcement would be more effective in the long-term.

> [If we think] 'I know that this is the correct answer' . . . then you do not allow the patient to participate. He then becomes a receiver. But if you share your knowledge . . . then you offer the patient an opportunity to think and decide by himself [175].

> Patients have to embrace our suggestion because they are convinced that it is the right one and not because we want them to choose a particular option. If you propose something

For peer review only - http://bmjopen.bmj.com/site/about/guidelines.xhtml

> that is inconsistent with their experience or knowledge, there is a risk that they will not listen to you [172].

The challenge of dual advocacy

This theme describes the HCP as simultaneously advocate of the patient and the healthcare system. Whilst representing the patients' interests, at the same time, HPCs are a cog within the system. It could feel like cogs were working against each other.

> It all ends up on our doorstep. It is not only we who face the system – we are mediators of sorts between the patient and the system. Not only must we work with the patient against the system, but with the system as well [162].

> As soon as someone gets sort of uncomfortable they will shift to a different prescriber and they will push them along a certain course . . . . and I honestly think it's like a ship without a rudder and it's just going round and round in circles[176].

At times it felt difficult to access specialist pain services, or that there was a mismatch between what was expected and received.

> There is a really big access issue with the pain clinics right now . . . while I can refer them, their likelihood of getting an appointment, even with strong advocacy from me, is very low[177]

> Often I find that they are not accomplishing any more than I was and [patients] are often sent back to me with them essentially saying, 'we did our best.' It's very frustrating, because if they were easy . . . they wouldn't have been referred [177].

Some described the benefits of a system where the cogs worked smoothly: the benefits of reciprocity and collaboration, being confident in the capabilities of colleagues and reciprocal respect.

> We get a lot of mileage about slapping each other on the back a little bit. And increasing other members of the team's confidence by respecting other members of the team, their profile is improved [178].

For peer review only - http://bmjopen.bmj.com/site/about/guidelines.xhtml

For peer review only

> If the team sort of echoes the same message and provides richness in terms of their different perspective on it . . . then I think there's less confusion for the poor clients and they're able to follow through on a unified evidence-based recommendation [169].

Personal costs

This theme describes the emotional costs of treating patients with chronic pain. First, the biomedical model could create a sense of professional failure for not being able to *fix*; 'how did we fail them?' [171].

> You become a doctor not to tell people I can't do anything, I can't find anything, you have this perception of yourself as well that you're going to sort it out and if you can't sort it out, it's frustrating. What's the point of you being there? [144]

> It's awful, and I think it's demoralizing when you leave people in pain. That's just so disrespectful. I mean you're supposed to be a doctor, you're supposed to relieve pain and suffering, and you ignore the pain [177].

However, an embodied bio-psychosocial approach which hinged on recognising human losses could incur a deep sense of personal loss. HPCs had to manage the tension between proximity and distance.

> We forget how much chronic pain affects the patient. They lose their jobs, they have emotional stress and depression and the depression itself is a big loss of productivity to the patient but also to the entire family and to the community[171]

> Trying to listen to the person . . . sort of empathise . . . . [but] almost protected professionally . . . trying to see where that person was coming from but not letting it become too personal . . . I've used the phrase detached empathy [163].

The craft of pain management

This describes clinical work as an experience-based competence or 'craft' [179] gained from experience rather than didactic education. At times HCPs felt under-skilled.

For peer review only - http://bmjopen.bmj.com/site/about/guidelines.xhtml

> The problem is, we don't know how to treat pain. And so everybody is telling me I'm not treating pain well, but nobody is helping me figure out how to treat the pain[180].

> I am not a psychologist . . . . I don't know whether it is fair to expect me to do all of that and I don't know if anyone is expecting me to. . . Someone bringing out a lot about their past or perhaps a very complex situation . . . we don't want to say the wrong thing and it be to someone's detriment . . . you don't want to open this can of worms[174].

Personal experience or maturity, patient mileage and learning from colleagues underpinned craft knowledge.

> One becomes more stable as a person [with age], and does not really have the same demands and does not believe that one can do everything, that one is able to solve everything . . . Young doctors can have in them, that they believe that they will solve everything[181].

> New grads can't learn all of this, they need a certain number of years, you can't teach them all of this[136].

Although some HCPs felt that clinical guidelines could support a more patient-centred approach [182, 183], there was a stronger sense that they constrained craft knowledge.

> Treatment has to be tailored to patient's needs and prescriptive guidelines promoting "one size fits all" is not acceptable[135].

> If you work according to the guidelines, you are constrained in your performance . . . what would be left of your independence, your own competence, your own practical experience . . . Am I to conclude then that my training was useless[183]

HCPs therefore used guidelines pragmatically within the remit of their own knowledge.

> I'm free to take or leave these things, to look at whether they suit my own ideas of how to approach my patients[183].

For peer review only - http://bmjopen.bmj.com/site/about/guidelines.xhtml

Conceptual model

The final phase of meta-ethnographic analysis is to develop a conceptual model that is abstracted from, *but more than the sum of*, the themes (figure 3). Through discussion XX, YY and ZZ developed a model which helps us to understand the experience of treating patients with chronic non-malignant pain. The model is underpinned by a series of tensions: (a) between a dualistic biomedical model and an embodied psychosocial model; (b) between professional distance and proximity; (c) between professional expertise and patient empowerment (d) between a need to make concessions in order to maintain relationships and known biomedical utility (e) between patient and healthcare system advocacy. We conceptualise these tensions, on a mixing console[1], as underpinning the craft of chronic non-malignant pain management. The poles are neither inherently *good* nor *bad*; just as bass and treble are neither inherently good nor bad. It is the correct mix within a context that contributes to the quality of music. Our console also incorporates the *pitch* or level of loss, both professional and personal, that can contribute to the harmony or dissonance of a therapeutic encounter.

Discussion

Our innovation is to present the first internationally relevant synthesis of healthcare professionals' experiences of treating chronic non-malignant pain. Already we know that, from the patient perspective, this experience can be adversarial [2]. Patients with chronic pain struggle to affirm their sense of self; their present and future appears unpredictable; they search for a credible explanation for their pain; they do not always feel heard, believed or valued by healthcare professionals; they struggle to prove themselves in the face of scepticism. We present our conceptual model as a mixing console that can help us to understand, think about and modify our experience of treating patients with chronic pain, particularly in cases where the encounter appears to be less rewarding. For example, consider: am I making a sudden shift to psychosocial explanations when I can find nothing biomedical or am I considering psychosocial factors alongside medical investigations; do I understand this patient's experience or am I too distant; have I discussed and negotiated the various options or am I trying to instruct and enforce; am I considering medical utility or am I making a concession (and is this concession for my benefit or my patient's benefit); am I effectively balancing my role as dual advocate? Beyond these dualities, our model encourages HCPs to consider the personal impact of treating patients with chronic non-malignant pain. How often do you find

[1] Idea for image of a mixing console from Cathy Jenkins, OUH NHS Foundation Trust, Oxford

For peer review only - http://bmjopen.bmj.com/site/about/guidelines.xhtml

yourself wondering whether you have failed as a professional? (professional loss) Are you feeling *bombarded by despair*? (personal loss). If the answer is yes to either of these, what measures are there in place to tackle this?

Our findings highlight the cultural scepticism that underpins the *siren song of diagnosis*, where HCPs and patients can be driven by the need for a diagnosis. This may help us to understand why patients with chronic pain often experience a strong sense of not being believed. They also demonstrate how the bio-psychosocial model can hide a continuing dualism, where HCPs prioritise biomedical findings and make an abrupt switch to psychosocial explanations when no diagnosis is found. This abrupt shift may explain patients' feeling of lost credibility. A more embodied non-dualistic bio-psychosocial approach at the outset would help HCPs to support patients with chronic pain. Our findings also demonstrate the complexity of navigating the geography between patients and HCPs. In this borderland, HCPs sometimes make concessions that are not evidence-based in order to maintain effective relationships. These concessions have policy and practice implications, for example, in the context of recent USA [184]and UK [185] guidelines on opioid prescription for chronic non-malignant pain, it might help to explain why an increasing number of HCPs are prescribing opioids despite very limited evidence for long-term opioid therapy for chronic pain outside of end-of-life care [186]. Our findings also have educational implications: for example, navigating relationships requires skills to finely balance the tensions inherent in the model whilst managing potential personal and professional losses. It might be useful for clinical educators to consider overlaps in training need between palliative care and chronic pain management. HPCs included in this review did not discuss their own personal life context which is also likely, at times, to contribute and make HPCs less resilient to personal or professional losses.

We need to consider that there is currently no agreed way of making an assessment of confidence for qualitative synthesis. We utilised the GRADE-CERQual to rate confidence as high when a theme was supported by a least half of the studies (n≥39). However, there are issues to be considered in our use of GRADE-CERqual: First, we know that there is limited agreement about what a good qualitative study is [22, 24]. Secondly, although weight (adequacy) and consistency (coherence) [25] of data can contribute to the gravity of a finding, it is important to consider that gravity has a qualitative component; a single idea can exert a strong gravitational pull. The Tale of the Emperor's New Clothes highlights the validity of a small voice in the crowd.

For peer review only - http://bmjopen.bmj.com/site/about/guidelines.xhtml

Findings from conceptual syntheses of qualitative research in health aim to provide ideas that can help to improve the experience of healthcare. We have presented a novel conceptual synthesis that helps us to understand, think about and modify our experience of treating patients with chronic non-malignant pain. Our conceptual model may be transferable to other patient groups or situations. We conceptualise these dualities, on a mixing console, as a useful way to frame the patient-clinician relationship. It would be useful for HPCs to consider their individual mix and contemplate a *re-mix* if necessary in order to successfully support people with chronic pain.

For peer review only

References

1. Department-of-Health: **Pain breaking through the barrier**. *Chief Medical Officer's report* 2009, **150th annual report of the Chief Medical Officer**.
2. Toye F, Seers K, Allcock N, Briggs M, Carr E, Andrews J, Barker K: **A meta-ethnography of patients' experiences of chronic non-malignant musculoskeletal pain**. *Health Services and Delivery Research* 2013, **1**(12):1-189.
3. Noyes J, Popay J, Pearson A, Hannes K, Booth A: **Qualitative Research and Cochrane Reviews**. In: *Cochrane Handbook for Systematic Reviews.* edn. Edited by Higgins J, Green S. Chichester, UK: John Wiley & Sons; 2008.
4. Britten N, Campbell R, Pope C, Donnovan J, Morgan M, Pill R: **Using meta ethnography to synthesise qualitative research: a worked example**. *Journal of Health Services and Research Policy* 2002, **7**(4):209-215.
5. Campbell R, Pound P, Pope C, Britten N, Pill R, Morgan M, Donovan J: **Evaluating meta-ethnography: a synthesis of qualitative research on lay experience of diabetes and diabetes care**. *Social Science & Medicine* 2003, **56**:671-684.
6. Malpass A, Shaw A, Sharp D, Walter F, Feder G, Ridd M, Kessler D: **"Medication career" or "Moral career"? The two sides of managing anti-dperessants: A meta-ethnography of patients experience of antidepressants**. *Social Science and Medicine* 2009, **68**:154-168.
7. Barker K, Toye F, MinnsLowe C: **A qualitative systematic review of patients' experience of osteoporosis using meta-ethnography**. *Archives of Osteoporosis* 2016, **11**(33):DOI 10.1007/s11657-11016-10286-z.
8. Toye F, Seers K, Allcock N, Briggs M, Carr E, Barker K: **A synthesis of qualitative research exploring the barriers to staying in work with chronic musculoskeletal pain**. *Disability & Rehabilitation* 2016, **38**(6):566-572.
9. Toye F, Seers K, Barker K: **A meta-ethnography of patients' experiences of chronic pelvic pain: struggling to construct chronic pelvic pain as 'real'**. *Journal of Advanced Nursing* 2014, **70**(12):2713-2727.
10. Noblit G, Hare R: **Meta-ethnography: Synthesising Qualitative Studies**. California: Sage Publications; 1988.
11. Toye F, Seers K, Allcock N, Briggs M, Carr E, Barker K: **Meta-ethnography 25 years on: challenges and insights for synthesising a large number of qualitative studies**. *BMC Medical Research Methodology* 2014, **14**(80).

For peer review only - http://bmjopen.bmj.com/site/about/guidelines.xhtml

12. Sandelowski M, Barrosso J: **Handbook for synthesising qualitative research**. New York: Springer Publishing Company; 2007.
13. Dixon-Woods M, Booth A, Sutton A: **Synthesizing qualitative research: a review of published reports**. *Qualitative Research* 2007, **7**:375-422.
14. Dixon-Woods M, Agarwal S, Jones D, Young B, Sutton A: **Synthesising qualitative and quantitative research evidence: a review of possible methods**. *Journal of Health Services and Research Policy* 2005, **10**(1):45-53.
15. Hannes K, Macaitis K: **A move to more systematic and transparent approaches in qualitative evidence synthesis: update on a review of published papers**. *Qualitative Research* 2012, **12** (4):402-442
16. Barnett-Page E, Thomas J: **Methods for synthesis of qualitative research: a critical review**. *Economic and Social Research Council Research Methods* 2009, **National Centre for Research Methods Working Paper Series**(01/09).
17. Campbell R, Pound P, Morgan M, Daker-White G, Britten N, Pill R, Yardley L, Pope C, Donovan J: **Evaluating meta-ethnography: systematic analysis and synthesis of qualitative research**. *Health Technology Assessment* 2011, **15**(43).
18. Wong SS WN, Haynes RB. : **Developing optimal search strategies for detecting clinically relevant qualitative studies in MEDLINE**. *Medinfo* 2004, **11**(1):311-316.
19. Wilczynski NL MS, Haynes RB. : **Search strategies for identifying qualitative studies in CINAHL**. *Qualitative Health Research* 2007, **17**(5):705-710.
20. McKibbon KA WN, Haynes RB. : **Developing optimal search strategies for retrieving qualitative studies in PsycINFO**. *Evaluation & the Health Professions* 2006, **29**(4):440-454.
21. Walters LA WN, Haynes RB; Hedges Team. : **Developing optimal search strategies for retrieving clinically relevant qualitative studies in EMBASE**. *Qualitative Health Research* 2006, **16**(1):162-168.
22. Dixon-Woods M, Sutton A, Shaw R, Miller T, Smith J, Young B, Bonas S, Booth A, Jones D: **Appraising qualitative research for inclusion in systematic reviews: a quantitative and qualitative comparison of three methods**. *Journal of Health Services and Research Policy* 2007, **12**(1):42-47.
23. CASP: **Critical Appraisal Skills Programme: making sense of evidence about clinical effectiveness: 10 questions to help you make sense of qualitative research**. *http://wwwcasp-uknet/wp-content/uploads/2011/11/CASP_Qualitative_Appraisal_Checklist_14oct10pdf* 2010.
24. Toye F, Seers K, Allcock N, Briggs M, Carr E, Andrews J, Barker K: **'Trying to pin down jelly' - exploring intuitive processes in quality assessment for meta-ethnography**. *BMC Medical Research Methodology* 2013, **13**:46.
25. Lewin S, Glenton C, Munthe-Kaas H, Carlsen B, Colvin CJ, Gülmezoglu M, Noyes J, Booth A, Garside R, Rashidian A: **Using Qualitative Evidence in Decision Making for Health and Social Interventions: An Approach to Assess Confidence in Findings from Qualitative Evidence Syntheses (GRADE-CERQual)**. *PLOS Medicine* 2016, **12**(10):e1001895. doi:1001810.1001371/journal. pmed.1001895.
26. Pope C, Mays N, Popay J: **Synthesizing Qualitative and Quantitative Health Research: A Guide to Methods**. Berkshire, UK: Open University Press; 2007.
27. Toye F, Seers K, Allcock N, Briggs M, Carr E, Andrews J, Barker K: **Patients' experiences of chronic non-malignant musculoskeletal pain: a qualitative systematic review**. *The British journal of general practice : the journal of the Royal College of General Practitioners* 2013, **63**(617).
28. McGee J, Kaylor D, Emmott H, Christopher J: **Defining chronic pain ethics**. *Pain Medicine* 2011, **12**(9):1376.
29. Lærum E, Indahl A, Skouen JS: **What is 'the good back-consultation'? A combined qualitative and quantitative study of chronic low back pain patients' interaction with and

For peer review only - http://bmjopen.bmj.com/site/about/guidelines.xhtml

perceptions of consultations with specialists. *Journal of Rehabilitation Medicine (Taylor & Francis Ltd)* 2006, **38**(4):255-263.
30. Rutledge N, Cantero P, Ruiz J: **Managing chronic pain in low income overweight Latinos: provider perceptions**. *Communicating Nursing Research* 2013, **46**:639-640.
31. Oien AM, Iversen S, Stensland P: **Narratives of embodied experiences - Therapy processes in Norwegian psychomotor physiotherapy**. *Advances in Physiotherapy* 2007, **9**(1):31-39.
32. Oien AM, Råheim M, Iversen S, Steihaug S: **Self-perception as embodied knowledge -- changing processes for patients with chronic pain**. *Advances in Physiotherapy* 2009, **11**(3):121-130.
33. Wootton IM, Wood V, Cook F: **Who wants expert patient programmes for chronic mechanical spinal pain?. An investigation into the value of, and recruitment to, an expert patient programme as part of the physiotherapy management of chronic spinal pain**. *Physiotherapy* 2008, **94**(1):78-84.
34. Thomson D: **An ethnographic study of physiotherapists' perceptions of their interactions with patients on a chronic pain unit**. *Physiotherapy theory and practice* 2008, **24**(6):408.
35. Bullington J, Nordemar R, Nordemar K, Sjöström-Flanagan C: **Meaning out of chaos: a way to understand chronic pain**. *Scandinavian Journal of Caring Sciences* 2003, **17**(4):325-332.
36. Doran J: **Experiencing Wellness Within Illness: Exploring a Mindfulness-Based Approach to Chronic Back Pain**. *Qualitative Health Research* 2014, **24**(6):749-761.
37. Howarth M, Warne T, Haigh C: **Pain from the inside: understanding the theoretical underpinning of person-centered care delivered by pain teams**. *Pain management nursing : official journal of the American Society of Pain Management Nurses* 2014, **15**(1):340-348.
38. Buchbinder M, Wilbur R, McLean S, Sleath B: **"Is there any way I can get something for my pain?" Patient strategies for requesting analgesics**. *Patient Education and Counseling* 2015, **98**(2):137-143.
39. Buijs PC, Lambeek LC, Koppenrade V, Hooftman WE, Anema JR: **Can workers with chronic back pain shift from pain elimination to function restore at work? Qualitative evaluation of an innovative work related multidisciplinary programme**. *Journal of Back and Musculoskeletal Rehabilitation* 2009, **22**(2):65-73.
40. Thunberg KA, Carlsson SG, Hallberg LRMRM: **Health care professionals' understanding of chronic pain: A grounded theory study**. *Scandinavian journal of caring sciences* 2001, **15**(1):99.
41. Valjakka AL, Salantera S, Laitila A, Julkunen J, Hagelberg NM: **The association between physicians' attitudes to psychosocial aspects of low back pain and reported clinical behaviour: A complex issue**. *Scandinavian Journal of Pain* 2013, **4**(1):25-30.
42. Kotarba A: **Perceptions of death, belief systems and the process of coping with chronic pain**. *Social Science & Medicine* 1983, **17**(10):681.
43. Cedraschi C, Robert J, Goerg D, Perrin E, Fischer W, Vischer TL: **Is chronic non-specific low back pain chronic? Definitions of a problem and problems of a definition**. *The British journal of general practice : the journal of the Royal College of General Practitioners* 1999, **49**(442):358.
44. Cedraschi C, Robert J, Perrin E, Fischer W, Goerg D, Vischer TL: **The role of congruence between patient and therapist in chronic low back pain patients**. *Journal of Manipulative and Physiological Therapeutics* 1996, **19**(4):244-249.
45. Sloan TJ, Walsh DA: **Explanatory and diagnostic labels and perceived prognosis in chronic low back pain**. *Spine* 2010, **35**(21).
46. Al-Shaer D, Hill PD, Anderson MA: **Nurses' knowledge and attitudes regarding pain assessment and intervention**. *Medsurg nursing : official journal of the Academy of Medical-Surgical Nurses* 2011, **20**(1):7-11.
47. Ferrell B: **Ethical perspectives on pain and suffering**. *Pain management nursing : official journal of the American Society of Pain Management Nurses* 2005, **6**(3):83.

For peer review only - http://bmjopen.bmj.com/site/about/guidelines.xhtml

48. Walker JM, Akinsanya JA, Davis BD, Marcer D: **The nursing management of elderly patients with pain in the community: study and recommendations**. *Journal of Advanced Nursing* 1990, **15**(10):1154-1162.
49. Gillmore R, Hill T: **Reactions to patients who complain of pain: Effects of ambiguous diagnosis**. *Journal of Applied Social Psychology* 1981, **11**(1):14.
50. Haq I, Fuller J, Dacre J: **The use of patient partners with back pain to teach undergraduate medical students**. *Rheumatology* 2006, **45**(4):430-434.
51. Jeffrey E, Foster E: **A qualitative investigation of physical therapists' experiences and feelings of managing patients with nonspecific low back pain**. *Physical therapy* 2012, **92**(2):266.
52. Skelton AM, Murphy EA, Murphy RJ, O'Dowd TC: **Patient education for low back pain in general practice**. *Patient education and counseling* 1995, **25**(3):329.
53. Skelton AM, Murphy EA, Murphy RJ, O'Dowd TC: **General practitioner perceptions of low back pain patients**. *Family practice* 1995, **12**(1):44.
54. Elder N, Ricer R, Tobias B: **How respected family physicians manage difficult patient encounters**. *Journal of the American Board of Family Medicine : JABFM* 2006, **19**(6):533.
55. Carr CJ, Worswick L, Wilcock M, Campion-Smith C, Hettinga D: **Improving services for back pain: putting the patient at the centre of interprofessional education**. *Quality in primary care* 2012, **20**(5):345.
56. Karstens S, Joos S, Hill JC, Krug K, Szecsenyi J, Steinhauser J: **General practitioners views of implementing a stratified treatment approach for low back pain in Germany: A qualitative study**. *PLoS ONE* 2015, **10**(8).
57. Roberts C, Adebajo AO, Long S: **Improving the quality of care of musculoskeletal conditions in primary care**. *Rheumatology (Oxford, England)* 2002, **41**(5):503.
58. Breen A, Austin H, Campion-Smith C, Carr E, Mann E: **"You feel so hopeless": A qualitative study of GP management of acute back pain**. *European Journal of Pain* 2007, **11**(1):21-29.
59. May C, Allison G, Chapple A, Chew-Graham C, Dixon C, Gask L, Graham R, Rogers A, Roland M: **Framing the doctor-patient relationship in chronic illness: A comparative study of general practitioners' accounts**. *Sociology of health & illness* 2004, **26**(2):135.
60. McIntosh A, Shaw FM: **Barriers to patient information provision in primary care: patients' and general practitioners' experiences and expectations of information for low back pain**. *Health expectations : an international journal of public participation in health care and health policy* 2003, **6**(1):19.
61. Miller S, Pinnington A: **Straightforward consultation or complicated condition? General practitioners' perceptions of low back pain**. *The European journal of general practice* 2003, **9**(1):3.
62. Rogers A: **Whose autonomy? Which choice? A study of GPs' attitudes towards patient autonomy in the management of low back pain**. *Family practice* 2002, **19**(2):140.
63. Sanders T, Foster E, Ong BN: **Perceptions of general practitioners towards the use of a new system for treating back pain: a qualitative interview study**. *BMC medicine* 2011, **9**:49.
64. Barker KL, Reid M, Lowe CJM: **Divided by a lack of common language? - A qualitative study exploring the use of language by health professionals treating back pain**. *BMC Musculoskeletal Disorders* 2009, **10**(1).
65. Billis V, McCarthy J, Stathopoulos I, Kapreli E, Pantzou P, Oldham A: **The clinical and cultural factors in classifying low back pain patients within Greece: A qualitative exploration of Greek health professionals**. *Journal of evaluation in clinical practice* 2007, **13**(3):337.
66. Magnusson JE, Fennell JA: **Understanding the role of culture in pain: Maori practitioner perspectives relating to the experience of pain**. *New Zealand Medical Journal* 2011, **124**(1328):41-51.

For peer review only - http://bmjopen.bmj.com/site/about/guidelines.xhtml

67. May S, Greasley A, Reeve S, Withers S: **Expert therapists use specific clinical reasoning processes in the assessment and management of patients with shoulder pain: a qualitative study**. *The Australian journal of physiotherapy* 2008, **54**(4):261-266.
68. McDonald DD: **Trialing to Pain Control: A Grounded Theory**. *Research in Nursing & Health* 2014, **37**(2):107-117.
69. Montali L, Monica C, Riva P, Cipriani R: **Conflicting representations of pain: A qualitative analysis of health care professionals' discourse**. *Pain Medicine* 2011, **12**(11):1585.
70. Ong BN, Hooper H: **Comparing clinical and lay accounts of the diagnosis and treatment of back pain**. *Sociology of Health & Illness* 2006, **28**(2):203-223.
71. Ong BN, Hooper H: **Involving users in low back pain research**. *Health Expectations* 2003, **6**(4):332-341.
72. Pincus T, Vogel S, Breen A, Foster N, Underwood M: **Persistent back pain - Why do physical therapy clinicians continue treatment? A mixed methods study of chiropractors, osteopaths and physiotherapists**. *European Journal of Pain* 2006, **10**(1):67-76.
73. Vegni E, Mauri E, Moja EA: **Stories from doctors of patients with pain. A qualitative research on the physicians' perspective**. *Supportive Care in Cancer* 2005, **13**(1):18-25.
74. Askew R, Kibelstis C, Overbaugh S, Walker S, Nixon-Cave K, Shepard KF: **Physical therapists' perception of patients' pain and its effect on management**. *Physiotherapy Research International* 1998, **3**(1):37-58.
75. Cruz B, Moore A, Cross V: **Clinical reasoning and patient-centred care in musculoskeletal physiotherapy in Portugal--a qualitative study**. *Manual therapy* 2012, **17**(3):246.
76. Dean G, Smith A, Payne S, Weinman J: **Managing time: an interpretative phenomenological analysis of patients' and physiotherapists' perceptions of adherence to therapeutic exercise for low back pain**. *Disability and rehabilitation* 2005, **27**(11):625.
77. Perreault K, Dionne CE, Rossignol M, Morin D: **Interprofessional practices of physiotherapists working with adults with low back pain in Quebec's private sector: Results of a qualitative study**. *BMC Musculoskeletal Disorders* 2014, **15**(1).
78. Sanders T, Foster E, Bishop A, Ong BN: **Biopsychosocial care and the physiotherapy encounter: physiotherapists' accounts of back pain consultations**. *BMC musculoskeletal disorders* 2013, **14**:65.
79. Smart K, Doody C: **The clinical reasoning of pain by experienced musculoskeletal physiotherapists**. *Manual therapy* 2007, **12**(1):40.
80. Francke AL, Lemmens ABT, Abu-Saad HH, Grypdonck M: **Nurses' perceptions of factors influencing the use of a pain program**. *Journal of Pain & Symptom Management* 1997, **14**(5):300-311.
81. Larsson H, Blomqvist K: **From a diagnostic and particular approach to a person-centred approach: a development project**. *Journal of Clinical Nursing* 2015, **24**(3/4):465-475.
82. Parke B: **Gerontological nurses' ways of knowing. Realizing the presence of pain in cognitively impaired older adults**. *Journal of gerontological nursing* 1998, **24**(6):21.
83. Droes NS: **Role of the nurse practitioner in managing patients with pain**. *Internet Journal of Advanced Nursing Practice* 2004, **6**(2):12p-NaN.
84. Schers H, Wensing M, Huijsmans Z, van M, Grol R: **Implementation barriers for general practice guidelines on low back pain a qualitative study**. *Spine* 2001, **26**(15):E348-353.
85. Bussieres AE, Patey AM, Francis JJ, Sales AE, Grimshaw JM, Canada PT, Brouwers M, Godin G, Hux J, Johnston M *et al*: **Identifying factors likely to influence compliance with diagnostic imaging guideline recommendations for spine disorders among chiropractors in North America: a focus group study using the Theoretical Domains Framework**. *Implementation science : IS* 2012, **7**.
86. Ottenheijm PG, Hesselmans JJM, Kemper A, Moser A, de AR, Dinant G-J, Cals WL: **GPs' perspectives on the diagnostic work-up in patients with shoulder pain: a qualitative study**. *Journal of evaluation in clinical practice* 2014, **20**(3):239.

For peer review only - http://bmjopen.bmj.com/site/about/guidelines.xhtml

87. Peiris D, Williams C, Holbrook R, Lindner R, Reeve J, Das A, Maher C: **A Web-based clinical decision support tool for primary health care management of back pain: Development and mixed methods evaluation**. *Journal of Medical Internet Research* 2014, **16**(4):1.
88. Stenner K, Courtenay M: **The role of inter-professional relationships and support for nurse prescribing in acute and chronic pain**. *Journal of Advanced Nursing* 2008, **63**(3):276-284.
89. Stenner K, Courtenay M: **Benefits of nurse prescribing for patients in pain: nurses' views**. *Journal of Advanced Nursing* 2008, **63**(1):27-36.
90. Bjorner T, Kjolsrod L: **How GPs understand patients' stories: A qualitative study of benzodiazepine and minor opiate prescribing in Norway**. *European Journal of General Practice* 2002, **8**(1):25-30.
91. Strand LI, Kvale A, Raheim M, Ljunggren AE: **Do Norwegian manual therapists provide management for patients with acute low back pain in accordance with clinical guidelines?** *Manual Therapy* 2005, **10**(1):38-43.
92. Haskins R, Osmotherly G, Southgate E, Rivett A: **Physiotherapists' knowledge, attitudes and practices regarding clinical prediction rules for low back pain**. *Manual therapy* 2014, **19**(2):142.
93. Josephson I, Bulow P, Hedberg B: **Physiotherapists' clinical reasoning about patients with non-specific low back pain, as described by the International Classification of Functioning, Disability and Health**. *Disability and Rehabilitation* 2011, **33**(23-24):2217-2228.
94. Josephson I, Hedberg B, Bulow P: **Problem-solving in physiotherapy--physiotherapists' talk about encounters with patients with non-specific low back pain**. *Disability and Rehabilitation* 2013, **35**(8):668-677.
95. Miller WL, Yanoshik MK, Crabtree BF, Reymond WK: **Patients, family physicians, and pain: visions from interview narratives**. *Family medicine* 1994, **26**(3):179.
96. Costa-Black KM, Durand M, Imbeau D, Baril R, Loisel P: **Interdisciplinary team discussion on work environment issues related to low back disability: a multiple case study**. *Work* 2007, **28**(3):249-266.
97. Gunby SS: **The lived experience of nursing students in caring for suffering individuals**. *Holistic nursing practice* 1996, **10**(3):63.
98. Eifried S: **Bearing witness to suffering: the lived experience of nursing students**. *The Journal of nursing education* 2003, **42**(2):59.
99. Chancellor J, Martin M, Liedgens H, Baker G, Müller-Schwefe HH: **Stated preferences of physicians and chronic pain sufferers in the use of classic strong opioids**. *Value in health : the journal of the International Society for Pharmacoeconomics and Outcomes Research* 2012, **15**(1):106.
100. Noll E, Key A, Jensen G: **Clinical reasoning of an experienced physiotherapist: insight into clinician decision-making regarding low back pain**. *Physiotherapy Research International* 2001, **6**(1):40-52.
101. Undeland M, Malterud K: **Diagnostic interaction: the patient as a source of knowledge?** *Scandinavian journal of primary health care* 2008, **26**(4):222.
102. Espeland A, Baerheim A: **General practitioners' views on radiology reports of plain radiography for back pain**. *Scandinavian Journal of Primary Health Care* 2007, **25**(1):15-20.
103. van WP, Beetsma A, Neels H, Roussel N, Nijs J: **Physical therapists should integrate illness perceptions in their assessment in patients with chronic musculoskeletal pain; a qualitative analysis**. *Manual therapy* 2014, **19**(3):229.
104. Steihaug S, Malterud K: **Part process analysis: a qualitative method for studying provider-patient interaction**. *Scandinavian Journal of Public Health* 2003, **31**(2):107-113.
105. Dysvik E, Furnes B: **Nursing leadership in a chronic pain management group approach**. *Journal of nursing management* 2012, **20**(2):187.

For peer review only

For peer review only - http://bmjopen.bmj.com/site/about/guidelines.xhtml

106. Baker C, Gallois C, Driedger SM, Santesso N: **Communication accommodation and managing musculoskeletal disorders: Doctors' and patients' perspectives**. *Health Communication* 2011, **26**(4):379.
107. Wermeling M, Scherer M, Himmel W: **GPs' experiences of managing non-specific neck pain - A qualitative study**. *Family Practice* 2011, **28**(3):300-306.
108. Nielsen M, Keefe J, Bennell K, Jull A: **Physical Therapist-Delivered Cognitive-Behavioral Therapy: A Qualitative Study of Physical Therapists' Perceptions and Experiences**. *Physical Therapy* 2014, **94**(2):197-210.
109. Kee CC, Epps CD: **Pain management practices of nurses caring for older patients with osteoarthritis**. *Western Journal of Nursing Research* 2001, **23**(2):195-211.
110. Manias E: **Complexities of pain assessment and management in hospitalised older people: A qualitative observation and interview study**. *International Journal of Nursing Studies* 2012, **49**(10):1243-1255.
111. Blondal K, Halldorsdottir S: **The challenge of caring for patients in pain: from the nurse's perspective**. *Journal of Clinical Nursing* 2009, **18**(20):2897-2907.
112. Hildebran C, Cohen J, Irvine M, Foley C, O'Kane N, Beran T, Deyo A: **How clinicians use prescription drug monitoring programs: a qualitative inquiry**. *Pain medicine (Malden, Mass)* 2014, **15**(7):1179.
113. Donovan J: **Learning disability nurses' experiences of being with clients who may be in pain**. *Journal of advanced nursing* 2002, **38**(5):458.
114. Ladak SJ, McPhee C, Muscat M, Robinson S, Kastanias P, Snaith K, Elkhouri M, Shobbrook C: **The journey of the pain resource nurse in improving pain management practices: Understanding role implementation**. *Pain Management Nursing* 2013, **14**(2):68.
115. Skjutar A, Schult M-L, Christensson K, Müllersdorf M: **Indicators of need for occupational therapy in patients with chronic pain: occupational therapists' focus groups**. *Occupational therapy international* 2010, **17**(2):93.
116. Wainwright D, Boichat C, McCracken M: **Competing patient and professional agendas in service development**. *Journal of health organization and management* 2014, **28**(6):777.
117. Hooten WM, Vickers S, Shi Y, Ebnet L, Townsend O, Patten A, Warner O: **Smoking cessation and chronic pain: Patient and pain medicine physician attitudes**. *Pain Practice* 2011, **11**(6):552.
118. Beattie A, Shaw A, Yardley L, Little P, Sharp D: **Participating in and delivering the ATEAM trial (Alexander technique lessons, exercise, and massage) interventions for chronic back pain: A qualitative study of professional perspectives**. *Complementary Therapies in Medicine* 2010, **18**(3-4):119-127.
119. Spiers J: **Expressing and responding to pain and stoicism in home-care nurse-patient interactions**. *Scandinavian Journal of Caring Sciences* 2006, **20**(3):293-302.
120. Tellier PP, Belanger E, Rodriguez C, Ware MA, Nancy PN: **Improving undergraduate medical education about pain assessment and management: A qualitative descriptive study of stakeholders' perceptions**. *Pain Research and Management* 2013, **18**(5):259-265.
121. Mackintosh-Franklin C: **The impact of experience on undergraduate preregistration student nurses' responses to patients in pain: A 2-year qualitative longitudinal study**. *Pain Management Nursing* 2014, **15**(1):199.
122. Samuels G, Leveille M: **Senior nursing students' clinical judgments in pain management**. *Nurse educator* 2010, **35**(5):220.
123. Walker JM: **Caring for elderly people with persistent pain in the community: a qualitative perspective on the attitudes of patients and nurses**. *Health & Social Care in the Community* 1994, **2**(4):221-229.
124. Parsons A, Mamdani M, Bhattacharyya O, Fortin CM, Melo M, Salmon C, Raptis R, Bain D, O'Campo P: **Narcotic analgesic utilization amongst injured workers: using concept mapping**

For peer review only - http://bmjopen.bmj.com/site/about/guidelines.xhtml

to understand current issues from the perspectives of physicians and pharmacists. *BMC health services research* 2011, **11**:280.
125. Kaasalainen S, Brazil K, Akhtar-Danesh N, Coker E, Ploeg J, Donald F, Martin-Misener R, DiCenso A, Hadjistavropoulos T, Dolovich L *et al*: **The evaluation of an interdisciplinary pain protocol in long term care**. *Journal of the American Medical Directors Association* 2012, **13**(7):664.
126. Tousignant-Laflamme Y, Tousignant M, Lussier D, Lebel P, Savoie M, Lalonde L, Choiniere M: **Educational needs of health care providers working in long-term care facilities with regard to pain management**. *Pain Research and Management* 2012, **17**(5):341-346.
127. Schofield P: **Talking to older people in care homes: Perceptions of their pain and their preferred management strategies. Results of a pilot study**. *International Journal on Disability and Human Development* 2006, **5**(1):53.
128. Corbett M, Foster N, Ong BN: **GP attitudes and self-reported behaviour in primary care consultations for low back pain**. *Family practice* 2009, **26**(5):359.
129. Crowe M, Whitehead L, Jo M, Baxter D, Panckhurst A: **Self-management and chronic low back pain: A qualitative study**. *Journal of Advanced Nursing* 2010, **66**(7):1478-1486.
130. Hadker N, Garg S, Chandran B, Crean M, McNett M, Silverman L: **Efficient practices associated with diagnosis, treatment and management of fibromyalgia among primary care physicians**. *Pain research & management : the journal of the Canadian Pain Society = journal de la société canadienne pour le traitement de la douleur* 2011, **16**(6):440.
131. Dysvik E, Stephens P: **Conducting rehabilitation groups for people suffering from chronic pain**. *International journal of nursing practice* 2010, **16**(3):233.
132. Kotarba A, Seidel V: **Managing the problem pain patient: Compliance or social control?** *Social Science & Medicine* 1984, **19**(12):1393.
133. Coutu MF, Legare F, Stacey D, Durand MJ, Corbiere M, Bainbridge L, Labrecque ME: **Occupational therapists' shared decision-making behaviors with patients having persistent pain in a work rehabilitation context: A cross-sectional study**. *Patient Education and Counseling* 2015, **98**(7):864-870.
134. Corrigan C, Desnick L, Marshall S, Bentov N, Rosenblatt A: **What can we learn from first-year medical students' perceptions of pain in the primary care setting?** *Pain medicine (Malden, Mass)* 2011, **12**(8):1216.
135. Wilson N, Pope C, Roberts L, Crouch R: **Governing healthcare: Finding meaning in a clinical practice guideline for the management of non-specific low back pain**. *Social Science & Medicine* 2014, **102**:138.
136. Slade SC, Molloy E, Keating JL: **The dilemma of diagnostic uncertainty when treating people with chronic low back pain: a qualitative study**. *Clinical Rehabilitation* 2011, **26**(6):558-570.
137. Wainwright D, Calnan M, O'Neil C, Winterbottom A, Watkins C: **When pain in the arm is 'all in the head': The management of medically unexplained suffering in primary care**. *Health, Risk & Society* 2006, **8**(1):71.
138. Afrell M, Rudebeck CE: **'We got the whole story all at once': physiotherapists' use of key questions when meeting patients with long-standing pain**. *Scandinavian journal of caring sciences* 2010, **24**(2):281.
139. Oien AM, Steihaug S, Iversen S, Raheim M: **Communication as negotiation processes in long-term physiotherapy: a qualitative study**. *Scandinavian Journal of Caring Sciences* 2011, **25**(1):53-62.
140. Macneela P, Gibbons A, McGuire B, Murphy A: **"We need to get you focused": general practitioners' representations of chronic low back pain patients**. *Qualitative Health Research* 2010, **20**(7):977-986.
141. Gropelli T, Sharer J: **Nurses' Perceptions of Pain Management in Older Adults**. *MEDSURG Nursing* 2013, **22**(6):375-383.

For peer review only - http://bmjopen.bmj.com/site/about/guidelines.xhtml

142. Eccleston C, De CACA, Rogers WS: **Patients' and professionals' understandings of the causes of chronic pain: blame, responsibility and identity protection**. *Social Science & Medicine* 1997, **45**(5):699-710.
143. Dobbs D, Baker T, Carrion IV, Vongxaiburana E, Hyer K: **Certified nursing assistants' perspectives of nursing home residents' pain experience: communication patterns, cultural context, and the role of empathy**. *Pain management nursing : official journal of the American Society of Pain Management Nurses* 2014, **15**(1):87-96.
144. Patel S, Peacock S, McKinley R, Carter DC, Watson PJ: **GPs' experience of managing chronic pain in a South Asian community--a qualitative study of the consultation process**. *Family practice* 2008, **25**(2):71.
145. Patel S, Peacock SM, McKinley RK, Clark-Carter D, Watson PJ: **GPs' perceptions of the service needs of South Asian people with chronic pain: A qualitative enquiry**. *Journal of Health Psychology* 2009, **14**(7):909-918.
146. Sloots M, Scheppers EF, Bartels EAC, Dekker JHM, Geertzen JHB, Dekker J: **First rehabilitation consultation in patients of non-native origin: factors that lead to tension in the patient-physician interaction**. *Disability & Rehabilitation* 2009, **31**(22):1853-1862.
147. Sloots M, Dekker HM, Pont M, Bartels AC, Geertzen HB, Dekker J: **Reasons of drop-out from rehabilitation in patients of Turkish and Moroccan origin with chronic low back pain in The Netherlands: a qualitative study**. *Journal of rehabilitation medicine* 2010, **42**(6):566.
148. Paulson M, Danielson E, Norberg A: **Nurses' and physicians' narratives about long-term non-malignant pain among men**. *Journal of Advanced Nursing* 1999, **30**(5):1097-1106.
149. Blomqvist K: **Older people in persistent pain: nursing and paramedical staff perceptions and pain management**. *Journal of Advanced Nursing* 2003, **41**(6):575-585.
150. Cameron A, Smith H, Schofield A: **HEALTH CARE PROFESSIONALS' ACCOUNTS OF CHRONIC PAIN MANAGEMENT FOR OLDER ADULTS**. *Journal of the Physiotherapy Pain Association* 2015(38):17-28.
151. Clark L, Jones K, Pennington K: **Pain assessment practices with nursing home residents**. *Western Journal of Nursing Research* 2004, **26**(7):733-751.
152. Fox P, Solomon P, Raina P, Jadad A: **Barriers and facilitators in pain management in long-term care institutions: A qualitative study**. *Canadian Journal on Aging* 2004, **23**(3):269-280.
153. Kaasalainen S, Coker E, Dolovich L, Papaioannou A, Hadjistavropoulos T, Emili A, Ploeg J: **Pain management decision making among long-term care physicians and nurses**. *Western journal of nursing research* 2007, **29**(5):561.
154. Löckenhoff E, Laucks S, Port D, Tung J, Wethington E, Reid MC: **Temporal horizons in pain management: understanding the perspectives of physicians, physical therapists, and their middle-aged and older adult patients**. *The Gerontologist* 2013, **53**(5):850.
155. Mentes C, Teer J, Cadogan P: **The pain experience of cognitively impaired nursing home residents: perceptions of family members and certified nursing assistants**. *Pain management nursing : official journal of the American Society of Pain Management Nurses* 2004, **5**(3):118.
156. Ruiz JG, Qadri SS, Nader S, Wang J, Lawler T, Hagenlocker B, Roos BA: **Primary care management of chronic nonmalignant pain in veterans: a qualitative study**. *Educational Gerontology* 2010, **36**(5):372-394.
157. Blomberg A-M, Hylander I, Törnkvist L: **District nurses' involvement in pain care: A theoretical model**. *Journal of clinical nursing* 2008, **17**(15):2022.
158. Bergman A, Matthias S, Coffing M, Krebs E: **Contrasting Tensions Between Patients and PCPs in Chronic Pain Management: A Qualitative Study**. *Pain Medicine* 2013, **14**(11):1689-1698.
159. Kaasalainen S, Brazil K, Coker E, Ploeg J, Martin-Misener R, Donald F, DiCenso A, Hadjistavropoulos T, Dolovich L, Papaioannou A *et al*: **An action-based approach to**

For peer review only - http://bmjopen.bmj.com/site/about/guidelines.xhtml

improving pain management in long-term care. *Canadian Journal on Aging* 2010, **29**(4):503-518.
160. MacNeela P, Gibbons A, McGuire B, Murphy A: **'We need to get you focused': general practitioners' representations of chronic low back pain patients**. *Qualitative Health Research* 2010, **20**(7):977-987.
161. Esquibel AY, Borkan J: **Doctors and patients in pain: Conflict and collaboration in opioid prescription in primary care**. *Pain* 2014, **155**(12):2575-2582.
162. Dahan R, Borkan J, Brown JB, Reis S, Hermoni D, Harris S: **The challenge of using the low back pain guidelines: A qualitative research**. *Journal of Evaluation in Clinical Practice* 2007, **13**(4):616-620.
163. Toye F, Jenkins S, Seers K, Barker K: **Exploring the value of qualitative research films in clinical education**. *BMC Medical Research Methodology* 2015, **15**(214):http://bmcmededuc.biomedcentral.com/articles/10.1186/s12909-12015-10491-12902.
164. Clarke EB, French B, Bilodeau ML, Capasso VC, Edwards A, Empoliti J: **Pain management knowledge, attitudes and clinical practice: The impact of nurses' characteristics and education**. *Journal of Pain and Symptom Management* 1996, **11**(1):18-31.
165. Berg M, Arnsten H, Sacajiu G, Karasz A: **Providers' experiences treating chronic pain among opioid-dependent drug users**. *Journal of general internal medicine* 2009, **24**(4):482.
166. Briones-Vozmediano E, Vives-Cases C, Ronda-Perez E, Gil-Gonzalez D: **Patients' and professionals' views on managing fibromyalgia**. *Pain Research and Management* 2013, **18**(1):19-24.
167. Baszanger I: **Deciphering chronic pain**. *Sociology of Health and Illness* 1992, **14**(2):181-215.
168. Foucault M: **The birth of the clinic**. Abingdon, Oxon, UK: Routledge Classics; 2003.
169. Cartmill C, Soklaridis S, Cassidy J: **Transdisciplinary teamwork: The experience of clinicians at a functional restoration program**. *Journal of occupational rehabilitation* 2011, **21**(1):1.
170. Scott-Dempster C, Toye F, Truman J, Barker K: **Physiotherapists' experiences of activity pacing with people with chronic musculoskeletal pain: an interpretative phenomenological analysis**. *Physiotherapy theory and practice* 2014, **30**(5):319.
171. Siedlecki L, Modic MB, Bernhofer E, Sorrell J, Strumble P, Kato I: **Exploring how bedside nurses care for patients with chronic pain: a grounded theory study**. *Pain management nursing : official journal of the American Society of Pain Management Nurses* 2014, **15**(3):565.
172. Zanini C, Sarzi-Puttini P, Atzeni F, Di FM, Rubinelli S: **Doctors' insights into the patient perspective: a qualitative study in the field of chronic pain**. *BioMed Research International* 2014:514230-514231.
173. Wynne-Jones G, van dD, Ong BN, Bishop A, Cowen J, Artus M, Sanders T: **Perceptions of health professionals towards the management of back pain in the context of work: a qualitative study**. *BMC musculoskeletal disorders* 2014, **15**:210.
174. Barker KL, Heelas L, Toye F: **Introducing Acceptance and Commitment Therapy to a physiotherapy led pain rehabilitation programme: An Action Research Study**. *British Journal of Pain* 2015.
175. Tveiten S, Meyer I: **'Easier said than done': Empowering dialogues with patients at the pain clinic – the health professionals' perspective**. *Journal of nursing management* 2009, **17**(7):804.
176. McCrorie C, Closs SJ, House A, Petty D, Ziegler L, Glidewell L, West R, Foy R: **Understanding long-term opioid prescribing for non-cancer pain in primary care: a qualitative study**. *BMC family practice* 2015, **16**:121.
177. Barry T, Irwin S, Jones S, Becker C, Tetrault M, Sullivan E, Hansen H, O'Connor G, Schottenfeld S, Fiellin A: **Opioids, chronic pain, and addiction in primary care**. *The journal of pain : official journal of the American Pain Society* 2010, **11**(12):1442.

For peer review only - http://bmjopen.bmj.com/site/about/guidelines.xhtml

178. Howarth M, Warne T, Haigh C: **"Let's stick together" – A grounded theory exploration of interprofessional working used to provide person centered chronic back pain services**. *Journal of interprofessional care* 2012, **26**(6):491.
179. Daykin R, Richardson B: **Physiotherapists' pain beliefs and their influence on the management of patients with chronic low back pain**. *Spine* 2004, **29**(7):783.
180. Spitz A, Moore A, Papaleontiou M, Granieri E, Turner J, Reid MC: **Primary care providers' perspective on prescribing opioids to older adults with chronic non-cancer pain: a qualitative study**. *BMC geriatrics* 2011, **11**:35.
181. Åsbring P, Närvänen A: **Ideal versus reality: physicians perspectives on patients with chronic fatigue syndrome (CFS) and fibromyalgia**. *Social Science & Medicine* 2003, **57**(4):711-721.
182. Poitras S, Durand M-J, Côté A-M, Tousignant M: **Use of low-back pain guidelines by occupational therapists: A qualitative study of barriers and facilitators**. *Work: Journal of Prevention, Assessment & Rehabilitation* 2011, **39**(4):465.
183. Harting J, Rutten GM, Rutten ST, Kremers P: **A qualitative application of the diffusion of innovations theory to examine determinants of guideline adherence among physical therapists**. *Physical therapy* 2009, **89**(3):221.
184. Dowell D, Haegerich TM, Chou R: **CDC (Centers for Disease Control and Prevention) Guideline for Prescribing Opioids for Chronic Pain — United States, 2016**. *Morbidity and Mortality Weekly* 2016, **Report 65, March 15**:1-49.
185. Royal-College-of-Anaesthetists: **Opioids Aware: A resource for patients and healthcare professionals to support prescribing of opioid medicines for pain**. 2016(8th November 2016).
186. Chou R, Deyo R, Devine B, Hansen R, Sullivan S, Jarvik JG, Blazina I, Dana T, Bougatsos C, Turner J: **The Effectiveness and Risks of Long-Term Opioid Treatment of Chronic Pain**. *The Evidence Report/Technology Assessment, Number 218* 2014, **Prepared by the Pacific Northwest Evidence-based Practice Center under Contract No. 290-2012-00014-I**(Agency for Healthcare Research and Quality (AHRQ) Publication No. 14-E005-EF):https://www.effectivehealthcare.ahrq.gov/ehc/products/557/1971/chronic-pain-opioid-treatment-report-141205.pdf.
187. Clark L, Fink R, Pennington K, Jones K: **Nurses' Reflections on Pain Management in a Nursing Home Setting**. *Pain Management Nursing* 2006, **7**(2):71.
188. Côté P, Clarke J, Deguire S, Frank JW, Yassi A: **Chiropractors and return-to-work: the experiences of three Canadian focus groups**. *Journal of manipulative and physiological therapeutics* 2001, **24**(5):309.
189. Daykin AR, Richardson B: **Physiotherapists' pain beliefs and their influence on the management of patients with low back pain**. *Spine* 2004, **29**(7):783-795.
190. Espeland A, Baerheim A: **Factors affecting general practitioners' decisions about plain radiography for back pain: implications for classification of guideline barriers--a qualitative study**. *BMC Health Services Research* 2003, **3**(1).
191. Hayes M, Myhal C, Thornton F, Camerlain M, Jamison C, Cytryn N, Murray S: **Fibromyalgia and the therapeutic relationship: Where uncertainty meets attitude**. *Pain Research & Management* 2010, **15**(6):385.
192. Hellström O, Bullington J, Karlsson G, Lindqvist P, Mattsson B: **Doctors' attitudes to fibromyalgia: a phenomenological study**. *Scandinavian journal of social medicine* 1998, **26**(3):232.
193. Holloway K, McConigley R: **Descriptive, exploratory study of the role of nursing assistants in Australian residential aged care facilities: The example of pain management**. *Australasian Journal on Ageing* 2009, **28**(2):70-74.

For peer review only - http://bmjopen.bmj.com/site/about/guidelines.xhtml

194. Krebs E, Bergman A, Coffing M, Campbell R, Frankel M, Matthias S: **Barriers to guideline-concordant opioid management in primary care—A qualitative study**. *The Journal of Pain* 2014, **15**(11):1148.

195. Kristiansson MH, Brorsson A, Wachtler C, Troein M: **Pain, power and patience--a narrative study of general practitioners' relations with chronic pain patients**. *BMC family practice* 2011, **12**:31.

196. Lundh C, Segesten K, Björkelund C: **To be a helpless helpoholic--GPs' experiences of women patients with non-specific muscular pain**. *Scandinavian journal of primary health care* 2004, **22**(4):244.

197. Parsons S, Harding G, Breen A, Foster N, Pincus T, Vogel S, Underwood M: **Will shared decision making between patients with chronic musculoskeletal pain and physiotherapists, osteopaths and chiropractors improve patient care?** *Family Practice* 2012, **29**(2):203-212.

198. Starrels L, Wu B, Peyser D, Fox D, Batchelder A, Barg K, Arnsten H, Cunningham O: **It made my life a little easier: primary care providers' beliefs and attitudes about using opioid treatment agreements**. *Journal of opioid management* 2014, **10**(2):95.

199. Stinson J, White M, Isaac L, Campbell F, Brown S, Ruskin D, Gordon A, Galonski M, Pink L, Buckley N *et al*: **Understanding the information and service needs of young adults with chronic pain: perspectives of young adults and their providers**. *The Clinical journal of pain* 2013, **29**(7):600.

200. Allegretti A, Borkan J, Reis S, Griffiths F: **Paired interviews of shared experiences around chronic low back pain: Classic mismatch between patients and their doctors**. *Family Practice* 2010, **27**(6):676-683.

201. Coutu MF, Baril R, Durand MJ, Cote D, Cadieux G: **Clinician-patient agreement about the work disability problem of patients having persistent pain: why it matters**. *Journal of Occupational Rehabilitation* 2013, **23**(1):82-92.

202. Fontana JS: **The social and political forces affecting prescribing practices for chronic pain**. *Journal of Professional Nursing* 2008, **24**(1):30-36.

203. Kilaru S, Gadsden M, Perrone J, Paciotti B, Barg K, Meisel F: **How do physicians adopt and apply opioid prescription guidelines in the emergency department? A qualitative study**. *Annals of emergency medicine* 2014, **64**(5):482.

204. O'Connor BB, Eisenberg DM, Buring JE, Liang CL, Osypiuk K, Levy DB, Wayne PM: **Within-team patterns of communication and referral in multimodal treatment of chronic low back pain patients by an integrative care team**. *Global Advances In Health and Medicine* 2015, **4**(2):36-45.

205. Scott-Dempster C, Toye F, Truman J, Barker K: **Physiotherapists' experiences of activity pacing with people with chronic musculoskeletal pain. An interpretative phenomenological analysis.** *Physiotherapy Theory & Practice* 2014, **30**(5):319-328.

206. Seamark D, Seamark C, Greaves C, Blake S: **GPs prescribing of strong opioid drugs for patients with chronic non-cancer pain: a qualitative study**. *The British journal of general practice : the journal of the Royal College of General Practitioners* 2013, **63**(617).

207. Oosterhof B, Dekker JH, Sloots M, Bartels EA, Dekker J: **Success or failure of chronic pain rehabilitation: the importance of good interaction - a qualitative study under patients and professionals**. *Disability and Rehabilitation* 2014, **36**(22):1903-1910.

208. Schulte E, Heift A, Welke J, Hermann K, Schäfer M, Boemke W: **Specialised pain therapy from the general practitioners' perspective: A qualitative assessment**. *Journal of Pain Management* 2010, **3**(3):263.

209. Shye D, Freeborn DK, Romeo J, Eraker S: **Understanding physicians' imaging test use in low back pain care: the role of focus groups**. *International journal for quality in health care : journal of the International Society for Quality in Health Care / ISQua* 1998, **10**(2):83.

For peer review only - http://bmjopen.bmj.com/site/about/guidelines.xhtml

210. Sloots M, Dekker JHM, Pont M, Bartels EA, Geertzen JHB, Dekker J: **Reasons for drop-out from rehabilitation in patients of Turkish and Moroccan origin with chronic low back pain in The Netherlands: a qualitative**. *Journal of Rehabilitation Medicine* 2010, **42**(6):566-574.
211. Chew-Graham C, May C: **Chronic low back pain in general practice: The challenge of the consultation**. *Family practice* 1999, **16**(1):46.
212. Baldacchino A, Gilchrist G, Fleming R, Bannister J: **Guilty until proven innocent: A qualitative study of the management of chronic non-cancer pain among patients with a history of substance abuse**. *Addictive Behaviors* 2010, **35**(3):270-272.
213. Gooberman-Hill R, Heathcote C, Reid M, Horwood J, Beswick D, Williams S, Ridd J: **Professional experience guides opioid prescribing for chronic joint pain in primary care**. *Family practice* 2011, **28**(1):102.
214. McConigley R, Toye C, Goucke R, Kristjanson J: **Developing recommendations for implementing the Australian Pain Society's pain management strategies in residential aged care**. *Australasian journal on ageing* 2008, **27**(1):45.
215. Hansson M, Bostrom C, Harms-Ringdahl K: **Living with spine-related pain in a changing society--a qualitative study**. *Disability & Rehabilitation* 2001, **23**(7):286-295.
216. Hellman T, Jensen I, Bergström G, Busch H: **Returning to work - a long-term process reaching beyond the time frames of multimodal non-specific back pain rehabilitation**. *Disability & Rehabilitation* 2015, **37**(6):499-506.
217. Liu YW: **Exploring nursing assistants' roles in the process of pain management for cognitively impaired nursing home residents: a qualitative study**. *Journal of Advanced Nursing* 2014, **70**(5):1065-1078.
218. Holloway K, McConigley R: **Understanding nursing assistants' experiences of caring for older people in pain: the Australian experience [corrected] [published erratum appears in PAIN MANAGE NURS 2009 Sep;10(3):115]**. *Pain Management Nursing* 2009, **10**(2):99-107.

For peer review only

Table 1: Geographic context, sample size, data collection and analytic approach, order of analysis and professional group/topic

AUTHOR/YEAR	COUNTRY	N	DATA COLLECTION	ANALYTIC APPROACH	ORDER OF ANALYSIS & PROFESSIONAL GROUP/TOPIC
AFRELL 2010	NORWAY	6	FOCUS GROUPS	PHENOMENOLOGY	4. SPECIALIST PHYSIOS
ALLEGRETTI 2010	USA	13	SEMI-STRUCTURED INTERVIEW	IMMERSION-CRYSTALLISATION	1. PRIMARY CARE PHYSICIANS/GPS
ASBRING 2003	SWEDEN	26	SEMI-STRUCTURED INTERVIEW	GROUNDED THEORY	5. FIBROMYALGIA
BALDACCHINO 2010	SCOTLAND	29	FOCUS GROUPS AND INTERVIEWS	FRAMEWORK ANALYSIS	8. OPIOID PRESCRIPTION
BARKER 2015	UK	7	SEMI-STRUCTURED INTERVIEWS	ACTION RESEARCH	4. SPECIALIST PHYSIOS
BARRY 2010	USA	23	SEMI-STRUCTURED INTERVIEW	GROUNDED THEORY	8. OPIOID PRESCRIPTION
BASZANGER 1992	FRANCE	NK	ETHNOGRAPHY	GROUNDED THEORY	6. CHRONIC PAIN SERVICES
BERG 2009	USA	16	SEMI-STRUCTURED INTERVIEW	THEMATIC ANALYSIS	8. OPIOID PRESCRIPTION
BERGMAN 2013	USA	14	INTERVIEWS	THEMATIC ANALYSIS	1. PRIMARY CARE PHYSICIANS/GPS
BLOMBERG 2008	SWEDEN	20	FOCUS GROUPS	GROUNDED THEORY	11. NURSING
BLOMQVIST 2003	SWEDEN	52	INTERVIEWS	CONTENT ANALYSIS	9. OLDER ADULTS
BRIONES-VOZMEDIANO 2013	SPAIN	9	SEMI-STRUCTURED INTERVIEW	DISCOURSE ANALYSIS	5. FIBROMYALGIA
CAMERON 2015	SCOTLAND	13	SEMI-STRUCTURED TELEPHONE INTERVIEWS	THEMATIC ANALYSIS	9. OLDER ADULTS
CARTMILL 2011	CANADA	10	SEMI-STRUCTURED INTERVIEW	GROUNDED THEORY	6. CHRONIC PAIN SERVICES
CHEW-GRAHAM 1999	UK	20	SEMI-STRUCTURED INTERVIEW	GROUNDED THEORY	1. PRIMARY CARE PHYSICIANS/GPS
CLARK 2004	USA	NK	FOCUS GROUPS	ETHNOGRAPHY	10. PAIN IN AGE CARE FACILITES
CLARK 2006	USA	103	SEMI-STRUCTURED INTERVIEWS	CONTENT ANALYSIS	10. PAIN IN AGE CARE FACILITES
COTE 2001	CANADA	30	FOCUS GROUPS	THEMATIC ANALYSIS	7. PAIN RELATED WORK DISABILITY
COUTU 2013	CANADA	5	SEMI-STRUCTURED INTERVIEWS	THEMATIC ANALYSIS	7. PAIN RELATED WORK DISABILITY
DAHAN 2007	ISRAEL	38	FOCUS GROUPS	IMMERSION-CRYSTALLIZATION	9. GUIDELINES
DAYKIN 2004	UK	6	SEMI-STRUCTURED INTERVIEWS	GROUNDED THEORY	3. PHYSIOTHERAPISTS
DOBBS 2014	USA	28	FOCUS GROUPS	CONTENT ANALYSIS	10. PAIN IN AGE CARE FACILITES
ECCLESTON 1997	UK	11	Q-ANALYSIS	Q-ANALYSIS	2. MIXED HEALTHCARE PROFESSIONALS

For peer review only - http://bmjopen.bmj.com/site/about/guidelines.xhtml

ESPELAND 2003	NORWAY	13	FOCUS GROUPS	PHENOMENOLOGY	9. GUIDELINES
ESQUIBEL 2014	USA	21	INTERVIEWS	IMMERSION-CRYSTALLIZATION	8. OPIOID PRESCRIPTION
FONTANA 2008	USA	9	SEMI-STRUCTURED INTERVIEW	EMANCIPATORY RESEARCH	8. OPIOID PRESCRIPTION
FOX 2004	CANADA	54	FOCUS GROUPS	THEMATIC ANALYSIS	10. PAIN IN AGE CARE FACILITES
GOOBERMAN-HILL 2011	UK	27	SEMI-STRUCTURED INTERVIEW	THEMATIC ANALYSIS	8. OPIOID PRESCRIPTION
GROPELLI 2013	USA	16	SEMI-STRUCTURED INTERVIEWS	CONTENT ANALYSIS	10. PAIN IN AGE CARE FACILITES
HANSSON 2001	SWEDEN	4	INTERVIEWS	GROUNDED THEORY	7. PAIN RELATED WORK DISABILITY
HARTING 2009	NETHERLANDS	30	FOCUS GROUPS	CONTENT ANALYSIS	9. GUIDELINES
HAYES 2010	CANADA	32	FOCUS GROUPS AND INTERVIEWS	GROUNDED THEORY	5. FIBROMYALGIA
HELLMAN 2015	SWEDEN	15	SEMI-STRUCTURED INTERVIEWS	THEMATIC ANALYSIS	7. PAIN RELATED WORK DISABILITY
HELLSTROM 1998	SWEDEN	20	INTERVIEWS	PHENOMENOLOGY	5. FIBROMYALGIA
HOLLOWAY 2009A	AUSTRALIA	6	SEMI-STRUCTURED INTERVIEWS	CONSTANT COMPARISON	10. PAIN IN AGE CARE FACILITES
HOLLOWAY 2009B#	AUSTRALIA	6	SEMI-STRUCTURED INTERVIEWS	CONSTANT COMPARISON	10. PAIN IN AGE CARE FACILITES
HOWARTH 2012	UK	9	INTERVIEWS AND FOCUS GROUPS	GROUNDED THEORY	6. CHRONIC PAIN SERVICES
KAASALAINEN 2007	CANADA	66	INTERVIEWS AND FOCUS GROUPS	GROUNDED THEORY	10. PAIN IN AGE CARE FACILITES
KAASALAINEN 2010A	CANADA	NK	INTERVIEWS AND FOCUS GROUPS	THEMATIC ANALYSIS	10. PAIN IN AGE CARE FACILITES
KAASALAINEN 2010B	CANADA	53	INTERVIEWS AND FOCUS GROUPS	CASE-STUDY ANALYSIS (YIN)	10. PAIN IN AGE CARE FACILITES
KILARU 2014	USA	61	SEMI-STRUCTURED INTERVIEW	GROUNDED THEORY	8. OPIOID PRESCRIPTION
KREBS 2014	USA	14	SEMI-STRUCTURED INTERVIEW	IMMERSION-CRYSTALLISATION	8. OPIOID PRESCRIPTION
KRISTIANSSON 2011	SWEDEN	5	INTERVIEWS	NARRATIVE ANALYSIS	1. PRIMARY CARE PHYSICIANS/GPS
LIU 2014	HONG KONG	49	INTERVIEWS AND FOCUS GROUPS	CONTENT ANALYSIS	10. PAIN IN AGE CARE FACILITES
LOCKENHOFF 2013	USA	44	FOCUS GROUPS	CONTENT ANALYSIS	2. MIXED HEALTHCARE PROFESSIONALS
LUNDH 2004	SWEDEN	14	FOCUS GROUPS	CONSTANT COMPARISON	1. PRIMARY CARE PHYSICIANS/GPS
MACNEELA 2010	IRELAND	12	CRITICAL INCIDENT INTERVIEW	THEMATIC ANALYSIS	1. PRIMARY CARE PHYSICIANS/GPS
MCCONIGLEY 2008	AUSTRALIA	34	INTERVIEWS AND FOCUS GROUPS	THEMATIC ANALYSIS	10. PAIN IN AGE CARE FACILITES
MCCRORIE 2015	UK	15	FOCUS GROUPS	GROUNDED THEORY	8. OPIOID PRESCRIPTION
MENTES 2004	USA	11	SEMI-STRUCTURED INTERVIEWS	THEMATIC ANALYSIS	10. PAIN IN AGE CARE FACILITES
OCONNOR 2015	USA	NK	ETHNOGRAPHY	CONSTANT COMPARISON	6. CHRONIC PAIN SERVICES

For peer review only - http://bmjopen.bmj.com/site/about/guidelines.xhtml

OIEN 2011	NORWAY	6	INTERVIEWS, FOCUS GROUPS, OBSERVATION	CASE STUDY (YIN)	3. PHYSIOTHERAPISTS
OOSTERHOF 2014	NETHERLANDS	10	INTERVIEWS AND OBSERVATION	THEMATIC ANALYSIS	6. CHRONIC PAIN SERVICES
PARSONS 2012	UK	19	SEMI-STRUCTURED INTERVIEWS	FRAMEWORK ANALYSIS	2. MIXED HEALTHCARE PROFESSIONALS
PATEL 2008	UK	18	SEMI-STRUCTURED INTERVIEW	THEMATIC ANALYSIS	1. PRIMARY CARE PHYSICIANS/GPS
PATEL 2009#	UK	18	SEMI-STRUCTURED INTERVIEW	THEMATIC ANALYSIS	1. PRIMARY CARE PHYSICIANS/GPS
PAULSON 1999	SWEDEN	21	INTERIEWS	PHENOMENOLOGY	5. FIBROMYALGIA
POITRAS 2011	CANADA	9	SEMI-STRUCTURED INTERVIEWS	THEMATIC ANALYSIS	9. GUIDELINES
RUIZ 2010	USA	21	FOCUS GROUPS AND INTERVIEWS	GROUNDED THEORY	9. OLDER ADULTS
SCHULTE 2010	GERMANY	10	SEMI-STRUCTURED INTERVIEW	THEMATIC ANALYSIS	1. PRIMARY CARE PHYSICIANS/GPS
SCOTT-DEMPSTER 2014	UK	6	SEMI-STRUCTURED INTERVIEWS	IPA*	4. SPECIALIST PHYSIOS
SEAMARK 2013	UK	22	INTERVIEWS AND FOCUS GROUPS	THEMATIC ANALYSIS	8. OPIOID PRESCRIPTION
SHYE 1998	USA	22	FOCUS GROUPS	IMMERSION-CRYSTALLIZATION	9. GUIDELINES
SIEDLECKI 2014	USA	48	INTERVIEWS	GROUNDED THEORY	11. NURSING
SLADE 2012	AUSTRALIA	23	FOCUS GROUPS	GROUNDED THEORY	3. PHYSIOTHERAPISTS
SLOOTS 2009	NETHERLANDS	4	SEMI-STRUCTURED INTERVIEWS	THEMATIC ANALYSIS	6. CHRONIC PAIN SERVICES
SLOOTS 2010	NETHERLANDS	10	SEMI-STRUCTURED INTERVIEWS	THEMATIC ANALYSIS	6. CHRONIC PAIN SERVICES
SPITZ 2011	USA	26	FOCUS GROUPS	THEMATIC ANALYIS	8. OPIOID PRESCRIPTION
STARRELS 2014	USA	28	TELEPHONE INTERVIEW	GROUNDED THEORY	8. OPIOID PRESCRIPTION
STINSON 2013	CANADA	17	FOCUS GROUPS	THEMATIC ANALYSIS	6. CHRONIC PAIN SERVICES
THUNBERG 2001	SWEDEN	22	INTERVIEWS	GROUNDED THEORY	6. CHRONIC PAIN SERVICES
TOYE 2015	UK	19	FOCUS GROUPS	GROUNDED THEORY	2. MIXED HEALTHCARE PROFESSIONALS
TVEITEN 2009	NORWAY	5	FOCUS GROUPS	CONTENT ANALYSIS	6. CHRONIC PAIN SERVICES
WAINWRIGHT 2006	UK	14	INTERVIEWS	THEMATIC ANALYSIS	1. PRIMARY CARE PHYSICIANS/GPS
WILSON 2014	UK	NK	INTERVIEWS, LETTERS, DOCUMENTS	ETHNOGRAPHY	9. GUIDELINES
WYNNE-JONES 2014	UK	17	SEMI-STRUCTURED INTERVIEWS	CONSTANT COMPARISON	7. PAIN RELATED WORK DISABILITY
ZANINI 2014	ITALY	17	SEMI-STRUCTURED INTERVIEWS	THEMATIC ANALYSIS	6. CHRONIC PAIN SERVICES

SAMPLE REPORTED IN TWO PAPERS
*INTERPRETIVE PHENOMENOLOGICAL ANALYSIS

For peer review only - http://bmjopen.bmj.com/site/about/guidelines.xhtml

Table 2: Confidence in review findings – GRADE-CERQual assessment

REVIEW FINDING	ADEQUACY NUMBER OF CONCEPTS	COHERENCE* NUMBER OF STUDIES/77	METHODOLOGICAL LIMITATIONS n = satisfactory (n= key)	RELEVANCE	OVERALL ASSESSMENT OF CONFIDENCE
SCEPTICAL CULTURAL LENS	42	29 [40, 135, 136, 138, 140, 142, 143, 149, 151, 158, 159, 161-163, 171, 176, 177, 180, 181, 187-199]	29 (0)	22 direct, 4 indirect, 2 partial, 1 uncertain	MODERATE
NAVIGATING JUXTAPOSED MODELS OF MEDICINE	77	44 [40, 136-138, 140, 142, 144, 146, 148-152, 157-159, 161, 163, 165-167, 169, 171, 174, 176, 177, 180-183, 188, 189, 192, 194, 196, 197, 199-206]	42 (2)	37 direct, 4 indirect, 2 partial, 1 uncertain	HIGH
NAVIGATING TREACHEROUS BORDERLAND	92	36 [40, 136-140, 154, 161-163, 165-167, 171-177, 181, 182, 188, 190, 192, 195, 196, 199-201, 205, 207-211]	34 (2)	29 direct, 3 indirect, 3 partial, 1 uncertain	MODERATE
THE CRAFT OF PAIN MANAGEMENT	60	31 [135, 136, 140, 141, 143, 152, 156, 158, 159, 162, 165, 171, 173, 174, 177, 178, 180-183, 188-190, 196, 197, 202, 206, 207, 212-214]	29 (2)	27 direct, 2 indirect, 1 partial, 1 uncertain	MODERATE
CHALLENGE OF DUAL ADVOCACY	70	36 [40, 136, 140, 141, 145, 150-153, 155-159, 162, 166, 169, 171, 173, 176-178, 182, 187, 188, 193, 195, 199, 204, 208, 209, 214-218]	35 (1)	26 direct, 4 indirect, 5 partial, 1 uncertain	MODERATE
PERSONAL COST	71	33 [138, 139, 143, 144, 149, 151-153, 155, 157, 158, 162, 163, 165, 166, 171-177, 181, 189, 192, 193, 195, 196, 199, 200, 205, 211, 217, 218]	32 (1)	28 direct, 4 indirect, 1 partial,	MODERATE

*15/371 concepts did not fit conceptual categories

For peer review only - http://bmjopen.bmj.com/site/about/guidelines.xhtml

Figure 1 Example search syntax for Medline

**(I) QUALITATIVE SUBJECT HEADINGS**	EXP QUALITATIVE RESEARCH<br>EXP INTERVIEWS AS TOPIC<br>EXP FOCUS GROUPS<br>NURSING METHODOLOGY RESEARCH<br>ATTITUDE TO HEALTH
**(II) QUALITATIVE FREE TEXT TERMS**	qualitative adj5 (theor* or study or studies or research or analysis)<br>ethno.ti,ab<br>emic or etic. ti,ab<br>phenomenolog*.ti,ab<br>hermeneutic*.ti,ab<br>heidegger* or husserl* or colaizzi* or giorgi* or glaser or strauss or (van and kaam*) or (van and manen) or ricoeur or spiegelberg* or merleau).ti,ab<br>constant adj3 compar*.ti,ab<br>focus adj3 group*.ti,ab<br>grounded adj3 (theor* or study or studies or research or analysis).ti,ab<br>narrative adj3 analysis.ti,ab<br>discourse adj3 analysis.ti,ab<br>(lived or life) adj3 experience*.ti,ab<br>(theoretical or purposive) adj3 sampl*.ti,ab<br>(field adj note*) or (field adj record*) or fieldnote*.ti,ab<br>participant* adj3 observ*.ti,ab<br>action adj research.ti,ab<br>(digital adj record) or audiorecord* or taperecord* or videorecord* or videotap*).ti,ab<br>(cooperative and inquir*) or (co and operative and inquir*) or (co-operative and inquir*) .ti,ab<br>(semi-structured or semistructured or unstructured or structured) adj3 interview*.ti,ab<br>(informal or in-depth or indepth or "in depth") adj3 interview*.ti,ab<br>(face-to-face" or "face to face") adj3 interview*.ti,ab<br>"ipa" or "interpretive phenomenological analysis".ti,ab<br>"appreciative inquiry".ti,ab<br>(social and construct*) or (postmodern* or post-structural*) or (post structural* or poststructural*) or (post modern*) or post-modern* or feminis*.).ti,ab<br>humanistic or existential or experiential.ti,ab
**(III) PAIN SUBJECT HEADINGS**	EXP BACK PAIN/ OR EXP CHRONIC PAIN/ OR EXP LOW BACK PAIN/ OR EXP MUSCULOSKELETAL PAIN/ OR EXP PAIN/ OR EXP PAIN CLINICS/.<br>EXP FIBROMYALGIA/<br>EXP PAIN MANAGEMENT/
**(IV) PAIN FREE TEXT TERMS**	(chronic* or persistent* or long-stand* or longstand* or unexplain* or un-explain*)<br>fibromyalgia<br>"back ache" or back-ache or backache<br>"pain clinic" or pain-clinic*<br>pain adj5 syndrome*

For peer review only

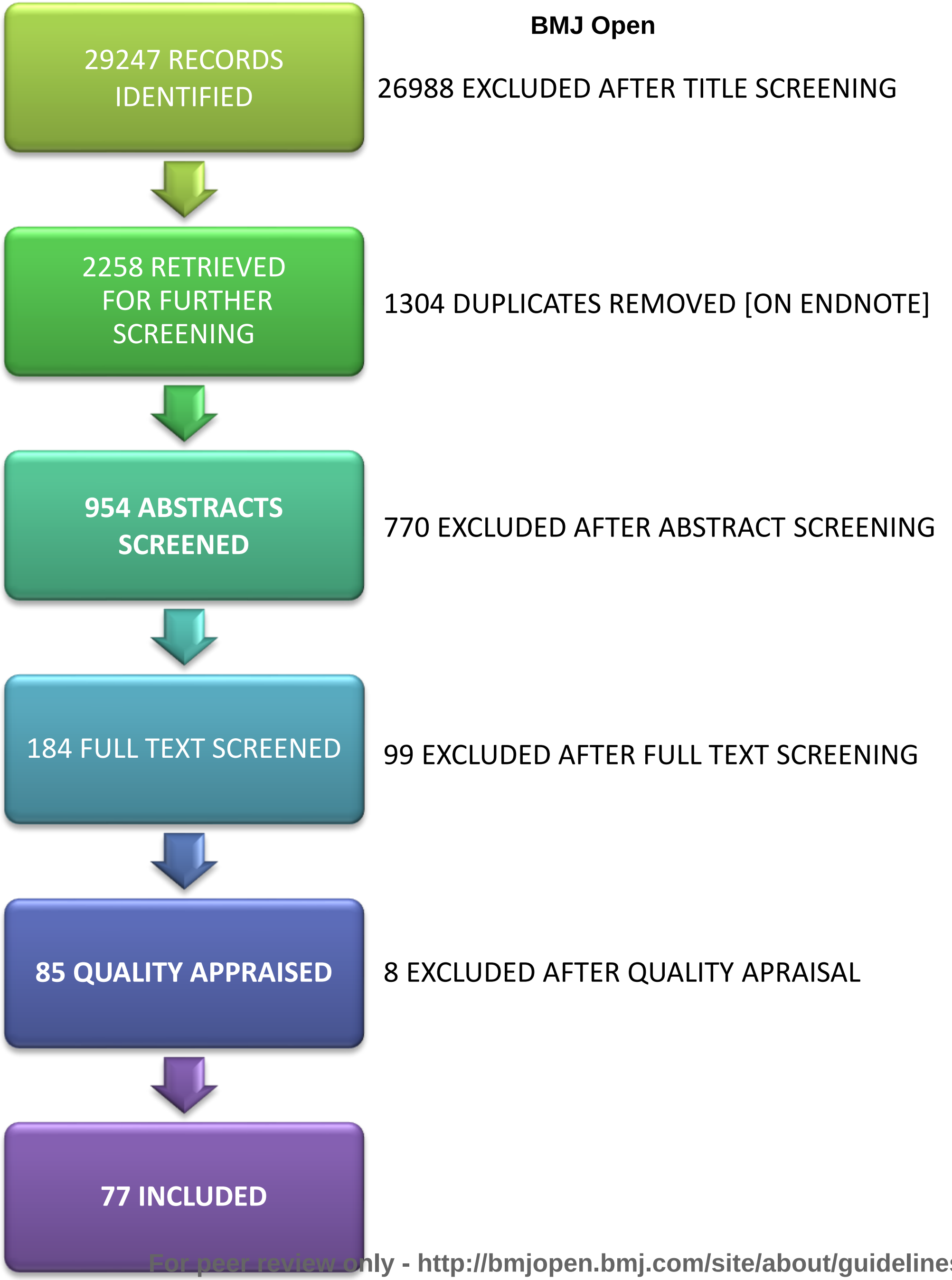
29247 RECORDS IDENTIFIED
26988 EXCLUDED AFTER TITLE SCREENING
2258 RETRIEVED FOR FURTHER SCREENING
1304 DUPLICATES REMOVED [ON ENDNOTE]
954 ABSTRACTS SCREENED
770 EXCLUDED AFTER ABSTRACT SCREENING
184 FULL TEXT SCREENED
99 EXCLUDED AFTER FULL TEXT SCREENING
85 QUALITY APPRAISED
8 EXCLUDED AFTER QUALITY APRAISAL
77 INCLUDED

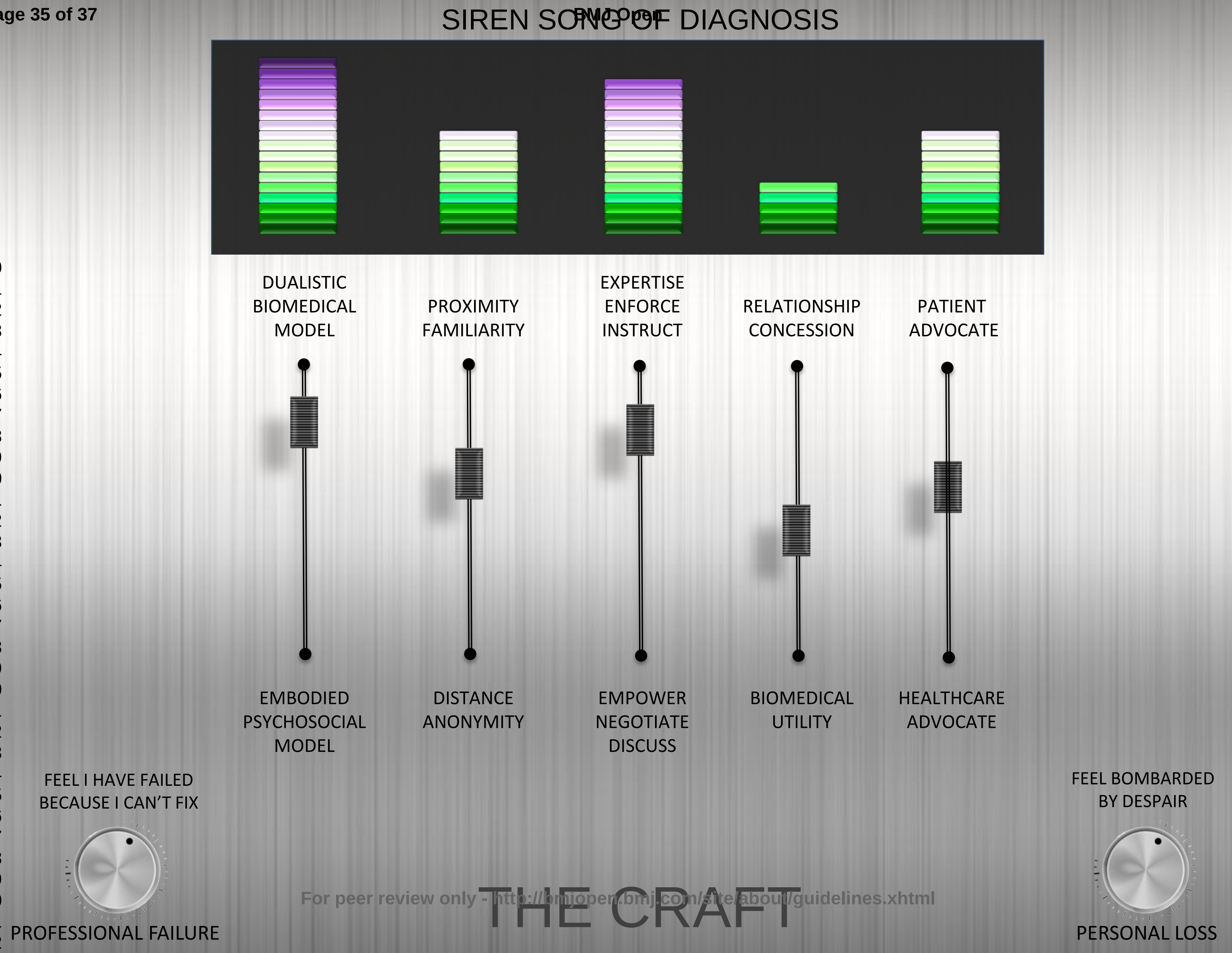
SIREN SONG OF DIAGNOSIS
DUALISTIC BIOMEDICAL MODEL
PROXIMITY FAMILIARITY
EXPERTISE ENFORCE INSTRUCT
RELATIONSHIP CONCESSION
PATIENT ADVOCATE
EMBODIED PSYCHOSOCIAL MODEL
DISTANCE ANONYMITY
EMPOWER NEGOTIATE DISCUSS
BIOMEDICAL UTILITY
HEALTHCARE ADVOCATE
FEEL I HAVE FAILED BECAUSE I CAN'T FIX
PROFESSIONAL FAILURE
FEEL BOMBARDED BY DESPAIR
PERSONAL LOSS
THE CRAFT

Appendix 1: Concepts not fitting the conceptual analysis

1. **BARRY 2010: Logistical Factors - Ancillary Staff**: Physicians expressed concern that they had insufficient qualified staff to implement pain management.
2. **BARRY 2010: Logistical Factors - Insurance Coverage:** Some physicians expressed concerns about the logistics of insurance coverage for pain management services and the difficulty in characterizing patients' pain status because of restrictions from insurance companies.
3. **FONTANA 2008: critical analysis:** A conflict of interest in which the patients' best interests are given a low priority. Nurses did not see prescribing decisions as ethical ones and, as a result, did not recognize the conflicts that were at work when they made these decisions.
4. **HOLLOWAY 2009A: Initiating clinical care**: The ability to provide pain management for residents when needed varied considerably between facilities; for some it involved basic care such as emotional support, positioning and using hot-packs, whereas in some facilities, they administered pain medication and had responsibility for monitoring the effectiveness of the pain management interventions and documentation.
5. **HOLLOWAY 2009B: Perfect Positioning (Rewards of Getting It Right):** Assistants felt sustained and fulfilled by the rewarding aspects of caring. All spoke of their passion for, enjoyment of, and love for their work (and this is why they stayed in it). Despite the emotional distress associated with observing people in pain, assistants gained satisfaction from seeing residents relieved of pain. Discussed poor financial remuneration they received and expressed the view that it was emotional fulfilment that made the job worthwhile.
6. **KAASALAINEN 2010A: interactions with long-term care staff and managers:** Nurse Practitioner was viewed as a nurse with added skills who assisted other healthcare team members with managing uncontrolled pain and was often used as an additional resource for nurses.
7. **LIU 2014: Instigator implementing non-pharmacological interventions**: Skills in distraction, reassurance and being gentle. Nursing assistants explained how they distracted or reassured residents who were in pain.
8. **LOCKENHOFF 2013: Age Differences in Time Horizons (treatment planning):** Consistently reported that they planned and administered pain management regimens for the long run.
9. **LUNDH 2004: variation 1:** ''I can feel very curious! What do these symptoms stand for?''
10. **OOSTERHOF 2014: Experiences concerning the treatment outcome (Learning new behaviour):** HCPs recognised that behaviour change takes a lot of effort, and requires a combination of explanation and practice. Some patients managed to learn new behaviour

For peer review only

and implement it within their daily life because they have always been active or because of their good body awareness or physical preference. Other might find it difficult to keep up effort due to personal problems and poor social support.

11. **SCOTT-DEMPSTER 2015: ''It's not a One Trick Pony'':** Physiotherapists regarded activity pacing as part of the pain management tool box to bring about change. Activity pacing was not described as something that was clearly definable or had fixed parameters. Achieving this flexibility could be challenging, as it meant that the physiotherapist had to adapt activity pacing for each individual.
12. **SEAMARK 2013: Cost:** Some did not consider cost and prescribed what was needed. Others felt it was important to bear in mind.
13. **SIEDLECKI 2014: CORE CONCEPTS/ TAKING OWNERSHIP:** Some did not take ownership of the problem and saw it as someone else's problem.
14. **STINSON 2013: Barriers to Care (patient-specific barriers):** Difficult to maintain a consistent pain management regimen because of time commitments and reluctance of younger people with pain.
15. **STINSON 2013: Pain Management Strategies (support systems)**: HCPs recognised the importance of peer support for patients.

For peer review only

BMJ Open

A meta-ethnography to understand healthcare professionals' experience of treating adults with chronic non-malignant pain

Journal:	*BMJ Open*
Manuscript ID	bmjopen-2017-018411.R1
Article Type:	Research
Date Submitted by the Author:	20-Sep-2017
Complete List of Authors:	Toye, Francine; Nuffield Orthopaedic Centre, Oxford, Nuffield Orthopaedic Centre<br>Seers, Kate; Royal College of Nursing Research Institute, Warwick Medical School, University of Warwick, RCN RI<br>Barker, Karen L.; University of Oxford, Nuffield Department of Orthopaedics, Rheumatology and Musculoskeletal Sciences
**Primary Subject Heading**:	Qualitative research
Secondary Subject Heading:	Patient-centred medicine
Keywords:	QUALITATIVE RESEARCH, PAIN MANAGEMENT, meta-ethnography, qualitative evidence synthesis, healthcare professionals

SCHOLARONE™
Manuscripts

A meta-ethnography to understand healthcare professionals' experience of treating adults with chronic non-malignant pain

Fran Toye (corresponding author) Nuffield Orthopaedic Centre, Oxford University Hospitals NHS Foundation Trust, Windmill Road, Oxford, OX3 7HE, UK; frantoye@hotmail.com; +44 1865 737526

Kate Seers, Royal College of Nursing Research Institute, Warwick Medical School, University of Warwick, Coventry, UK

Karen Barker, Nuffield Department of Orthopaedics, Rheumatology and Musculoskeletal Sciences (NDORMS), University of Oxford, Oxford, UK.

KEY WORDS

QUALITATIVE RESEARCH

PAIN MANAGEMENT

Meta-ethnography

Healthcare professionals

Word count 6488

For peer review only - http://bmjopen.bmj.com/site/about/guidelines.xhtml

Abstract

Objectives: We aimed to explore healthcare professionals' experience of treating chronic non-malignant pain by conducting a qualitative evidence synthesis. Understanding this experience from the perspective of healthcare professionals will contribute to improvements in the provision of care.

Design: Qualitative evidence synthesis using meta-ethnography. We searched five electronic bibliographic databases from inception to November 2016. We included studies that explore healthcare professionals' experience of treating adults with chronic non-malignant pain. We utilised the GRADE-CERQual framework to rate confidence in review findings.

Results: We screened the 954 abstracts and 184 full texts and included 77 published studies reporting the experiences of over 1551 international healthcare professionals including doctors, nurses and other health professionals. We abstracted 6 themes: (1) a sceptical cultural lens; (2) navigating juxtaposed models of medicine; (3) navigating the geography between patient and clinician; (4) challenge of dual advocacy; (5) personal costs; (6) the craft of pain management. We rated confidence in review findings as moderate to high.

Conclusions: This is the first qualitative evidence synthesis of healthcare professionals' experiences of treating people with chronic non-malignant pain. We have presented a model that we developed to help healthcare professionals to understand, think about and modify their experiences of treating patients with chronic pain. Our findings highlight scepticism about chronic pain that might explain why patients feel they are not believed. Findings also indicate a dualism in the bio-psychosocial model and the complexity of navigating therapeutic relationships. Our model may be transferable to other patient groups or situations.

For peer review only

For peer review only - http://bmjopen.bmj.com/site/about/guidelines.xhtml

Strengths and limitations of this study

- This study brings together, for the first time, a large number of qualitative studies (n= 77) that explore the experience of healthcare professionals' experience of treating people with chronic non-malignant pain.
- Meta-ethnography provides the reviewers' interpretation of qualitative findings abstracted into a line of argument with the aim of providing food for thought.
- There is no consensus on how to assess the quality of primary qualitative studies.
- Although the GRADE-CERQual provides a useful framework for determining confidence in qualitative syntheses, there is currently no consensus on how to do this.

The original protocol

The original study protocol and supporting documentation can be found at:

https://www.journalslibrary.nihr.ac.uk/programmes/hsdr/1419807/#/

A short film presenting the key themes can be found on YouTube (https://www.youtube.com/watch?v=477yTJPg10o)

Funding statement

National Institute for Health Research Health Services and Delivery Research Programme (14/198/07). The views and opinions expressed therein are those of the authors and do not necessarily reflect those of the HS&DR Programme, NIHR, NHS or the Department of Health.

Competing interests statement.

This study was funded by the National Institute for Health Research Health Services and Delivery Research Programme (14/198/07). KB and FT authored 2 of the 77 studies included in this review.

Data sharing statement

The original protocol, full monograph and supporting material can be found at: http://www.nets.nihr.ac.uk/projectsOld/hsdr/1419807

Contributor statement

FT, KB and KS made a substantial contribution to the design, acquisition, analysis and interpretation of data. FT drafted the first, subsequent and final versions. KS and KB revised all versions for important intellectual content and approved the final version. All authors agree to be accountable for the accuracy and integrity of the work

For peer review only - http://bmjopen.bmj.com/site/about/guidelines.xhtml

Background

Chronic pain is defined as pain which persists beyond the three months expected time of healing [1]. In 2009, an estimated five million people in the UK develop chronic pain [2], and a recent systematic review suggests that this may underestimate the problem[3]. Around 20% of adults in Europe have chronic pain [4] and in the USA more than 25 million adults (11%) experience chronic pain [5]. Chronic pain is challenging because it persists beyond healing time and is not easy to explain or treat. A range of clinical staff are involved in caring for people with chronic pain and in the UK there is a wide range in the provision of specialist care [6]. Not all patients with chronic pain have access to specialist services, and a national UK audit in 2012 indicated that only 40% of pain clinics met the minimum stand of having a psychologist, physiotherapist and physician [6]. The audit suggests that as many as 20% of patients with chronic pain visit accident and emergency departments even after visiting their general practitioner (GP), and as many as 66% visit a clinician three times within a six month period. A survey of undergraduate pain curricula for healthcare professionals in the UK [7] indicates that although these curricula are available, pain education is highly variable and 'woefully inadequate given the prevalence and burden of pain' [7] (page 78).

The Cochrane Qualitative Research Methods Group acknowledges the importance of including qualitative findings within evidence based healthcare [8]. Qualitative evidence synthesis (QES) aims to bring together qualitative research findings to make them accessible for public, policy, practice and education. A recent synthesis of 11 qualitative evidence syntheses (QES) has highlighted the personal challenge of living with chronic non-malignant pain and the loss of personal credibility that is integral to this experience [9]. Findings from a QES of 77 qualitative studies exploring patients experience of living with chronic non-malignant pain also demonstrate that patients can experience healthcare as an adversarial battle [10]. Understanding this from the perspective of healthcare professionals (HCPs) will help us to unpick this experience and thus contribute to improvements in care provision. Although there is a large body of qualitative research exploring HCPs experience of treating chronic non-malignant pain, there has been no attempt to systematically search for and integrate this knowledge into a QES. We aimed to conduct a QES using the methods of meta-ethnography [11]. Meta-ethnography is widely used and has provided insight into healthcare experiences such as; medicine taking [12], diabetes [13] antidepressants [14], osteoporosis [15], chronic musculoskeletal pain [10, 16] and chronic pelvic pain [17].

For peer review only - http://bmjopen.bmj.com/site/about/guidelines.xhtml

Methods

Meta-ethnography is a method developed by Noblit and Hare which aims to synthesise qualitative research findings into a whole that is greater than the sum of its original parts [11]. We used the methods of Meta-ethnography developed, refined and reported by Toye and colleagues [10, 18]. There are various methods for synthesising qualitative research [19-23]. An important distinction is between (a) those that *describe* findings and (b) those, like meta-ethnography, that develop *conceptual* understandings through a process of constant comparison and abstraction [11]. There are seven stages to meta-ethnography: getting started, deciding what is relevant, reading the studies, determining how studies are related, translating studies into each other, synthesising translations and expressing the synthesis [11].

In their original text, Noblit and Hare do not advocate an exhaustive search [11] and the number of studies included in meta-ethnographies ranges [20, 22, 24]. Unlike quantitative syntheses, qualitative syntheses do not aim to summarise the entire body of available knowledge or make statistical inference. We searched five electronic bibliographic databases (Medline, Embase, Cinahl, PsycINFO, Amed) using terms adapted from the InterTASC Information Specialists' Sub-Group (ISSG) Search Filter Resources [25-28]. We used subject headings and free text terms for *qualitative research*, combined with subject heading and free text terms for *pain* (table 1). We did not include citation checks, hand searching, grey literature or PhDs as in our previous QES 95% of the included studies were identified in the first three databases searched [10]. We included studies that explored the experience of all clinical healthcare staff involved in the care of patients with chronic pain. We excluded: acute pain, head pain and arthritic conditions. XX and YY screened the titles, abstracts and full text of potential studies.

There is currently no consensus on what makes a qualitative study *good enough* for QES [24, 29]. However, a growing number of reviewers are appraising studies for QES [22]. We did not intend to use rigid guidelines, but felt it important to seriously consider quality. We used three methods of appraisal: (a) The Critical Appraisal Skills Programme (CASP) questions for qualitative research [30]; (b) constructs from a qualitative study in a previous meta-ethnography [31]; (c) a global appraisal of whether the study was: 'key' (conceptually rich); 'satisfactory'; 'irrelevant'; or 'fatally flawed' [29]. As some journals are not explicit about ethical approval, we screened potential studies for ethical standards (CASP question 7: Have ethical issues been taken into consideration? [30]). If XX and YY did not agree about inclusion, they consulted ZZ for a final decision. We utilised the GRADE-CERQual framework [32] which aims to rate how much confidence readers can place in review findings. GRADE-CERQual suggest four domains: (1) '*Methodological limitations*'; (2) '*Relevance*' ; (3)

For peer review only - http://bmjopen.bmj.com/site/about/guidelines.xhtml

'*Adequacy of data'* (the 'degree of richness and quantity of data supporting a review finding'); *(4)* '*Coherence'* (consistency across primary studies); and finally, an overall rating of confidence (high, moderate, low, very low).

We planned to develop a line of argument synthesis, which involves 'making a whole into something more than the parts alone imply' [11] (page 28). Analysis in large QES involves a process of: identifying concepts from qualitative studies; abstracting these concepts into conceptual categories; further abstracting categories into themes; and finally developing a line of argument that makes sense of the themes. We read studies in batches of topic or professional grouping. We did not use an *index* paper to *orientate* the synthesis [33], as we felt that this choice can have a dramatic impact on the interpretation [18]. Two reviewers read each paper to identify, describe and list concepts. If they agreed that there was no clear concept then it was excluded. Through constantly comparing and discussing concepts three reviewers abstracted concepts into conceptual categories, using NVivo 11 software for qualitative analysis to keep track of our analytical decisions [34]. NVivo is particularly useful for collaborative analysis as it allows the team to keep a record and compare interpretations. Once we had agreed and defined conceptual categories, these were printed onto cards and sent to our advisory group to read and sort into thematic groups. This group consisted of patients, allied health professionals, nursing professionals, doctors and managers. Then, during advisory meeting, the reviewers worked alongside the advisory group to finalise the themes that would be included in the line of argument. In this way, we were able to challenge our own interpretations. Some reviewers do not present a line of argument as part of their QES findings. Frost and colleagues indicate that there has been a move away from interpretation and theory development in QES towards aggregative forms of synthesis [35]. The final analytic stage, 'synthesising translations' involved the three reviewers working together alongside the advisory group to craft the final themes into a 'line of argument' to build up a picture of the whole, grounded in the themes.

For peer review only

Findings

We retrieved 184 full texts and excluded 101 studies (figure 1). We excluded 16 studies that were not qualitative or that included limited qualitative data [36-51]. We agreed that 85 studies were out of scope [52-136] (for example, they did not present the HCP voice, or they did not explore the experience of chronic pain). Of the 83 studies remaining, we unanimously excluded six on the grounds of methodological report [137-142]. XX and YY unanimously appraised five studies as 'key papers' [143-147] and the remaining studies were appraised as 'satisfactory'. They did not agree about four studies [148-151] which were subsequently included. We included 77 published studies [143, 144, 146-220] reporting the experiences of over 1551 HCPs from USA (20 studies), UK (18

For peer review only - http://bmjopen.bmj.com/site/about/guidelines.xhtml

studies), Canada (10 studies), Sweden (10 studies), Netherlands (4 studies), Norway (4 studies), Australia (4 studies), France (4 studies), Germany (1 study), Hong Kong (1 study), Ireland (1 study), Israel (1 study), Italy (1 study) and Spain (1 study) (table 2). We agreed that ethical issues had been satisfactorily considered in the study design of all 77 studies and none were excluded on ethical grounds. Six studies were published before 2000; 37 were published between 2000 and 2010; 34 were published from 2011 onwards. HCPs included doctors, nurses and other health professionals in various contexts and geographical locations. Not all of the studies reported the number of participants from specific professional groups, which means that it was not possible to give the exact number of participants from each profession. Table 2 shows the author, year of publication, country, sample size, data collection, analytic approach, and professional group/context, participants and study focus. The studies explored the experience of: general practitioners (10 studies); mixed HCPs in diverse contexts (4 studies); physiotherapists (3 studies); physiotherapists with a speciality in chronic pain (3 studies); mixed HCPs in Fibromyalgia (5 studies); mixed HCPs in chronic pain services (11 studies); mixed HCPs in pain management related to employment (5 studies); mixed HCPs prescribing opioids to patients with chronic pain (12 studies); mixed HCPs utilising guidelines for chronic pain (6 studies); mixed HCPs working with older adults with chronic pain (3 studies); mixed HCPs working in long term care facilities (13 studies); nurses (2 studies).

Two reviewers identified 371 concepts from the 77 studies included. They organised the 371 concepts into 42 conceptual categories and then into six themes: Fifteen out of 371 concepts did not fit our analysis (appendix 1). There were several topics with insufficient weight to develop robust themes: ethnicity [172, 201, 202, 211, 212]; gender [203] and older people [160, 161, 163, 166, 176, 186, 193, 197, 205]. These may indicate useful areas of further research. Experience specific to opioid prescribing is reported elsewhere. A short film presenting the key themes is available on YouTube [https://www.youtube.com/watch?v=477yTJPg10o] and a report giving further details of analytical decisions is being published by the NIHR Journals library https://www.journalslibrary.nihr.ac.uk/programmes/hsdr/1419807/#/.

The six final themes were:(1) a sceptical cultural lens; (2) navigating juxtaposed models of medicine; (3) navigating the geography between patient and clinician; (4) the challenge of dual advocacy; (5) personal cost; (6) the craft of pain management. These themes are illustrated below with narrative exemplars. Indicators of confidence in each review findings are shown in table 3, which shows: the number of studies rated as key/valuable or satisfactory (*methodological limitations*); the number of concepts (*adequacy*); the number of studies out of 77 (*coherence*); an assessment of study relevance; our overall assessment of confidence. We rated our confidence in the review finding as

For peer review only - http://bmjopen.bmj.com/site/about/guidelines.xhtml

high when it was supported by more than half of the studies (n≥39). However, there is currently no agreed way of making an assessment of confidence for QES. We aimed to explore healthcare professionals' experience of treating chronic non-malignant pain. We found that studies explored the experiences of diagnosing *and* treating chronic pain, and that these experiences were inextricably linked. The studies supporting each theme are shown in table 3. The themes are drawn from a wide range of HCPs, including those specialising in chronic pain management who may be more likely to adopt a biopsychosocial approach [146, 147, 155, 157, 164, 185, 198, 199, 211, 212, 215, 217, 220]

A sceptical cultural lens

This theme describes a culturally entrenched sceptical view of chronic non-malignant pain from which HCPs did not always trust patients' reports of pain. This lack of trust meant that clinical work involved determining whether the pain was *something* or *nothing*. HCPs found themselves making judgements, based on personal factors rather than clinical findings, about whether the pain was *real* or *imagined*. They pondered dissonance between what the patient said and what the HCP could see.

> Sometimes I could have a patient sitting there and saying that they are hurting, 10 out of 10, and they are sitting like you and I[159]. [Bergman 2013, primary care, GP, USA]

> Some people say "This is the worst pain I've had in my whole life" without any real sort of physical signs of pain so it's really tough; we have a complex job in assessing that [188]. [Kaasalainen 2010b, aged care facilities, unspecified HCP, Canada]

There was a sense that HCPs were 'on guard' [149] against exploitation from fraudulent claims. For example, the following family practitioners felt concerned about being 'manipulated 'or 'exploited' by patients:

> It is not clear to me why he is the way he is... this catastrophic pain and what he is telling himself about it . . . but there is always a little bit . . . of concern; am I being manipulated, is this really real? [174] [Esquibel 2014, opioid prescription, family practitioner, USA]

> Such people . . . ones whose wishes you cannot fathom – provoke anger and frustration because at some point, you don't always know how to verify their complaints. You feel somewhat exploited. It is a very unpleasant feeling [170] [Dahan 2007, guidelines, GP, Israel]

HCPs engaged in a process of categorisation to decipher patients' truth claims. This categorisation hinged upon deciphering a multiplicity of dualities which were superimposed on a polarity of *'good'* and *'bad.* HCP described these dualities as follows: easy/difficult; explained/not explained, local/diffuse pain; adherent/non-adherent; stoical/weak; motivated/unmotivated;

For peer review only - http://bmjopen.bmj.com/site/about/guidelines.xhtml

accepting/resisting; non-complaining/complaining; deserving/non-deserving. Some recognised that this categorisation was flawed and advocated trust as the basis of the therapeutic relationship. For example the following HCPs describe how at times they had made the wrong judgement about patients who were truly in pain:

> Sometimes we say 'oh she came in with back pain but I don't think she's really in pain'. . . but really even if somebody is in pain and distress, [it] doesn't always have to be in how they present themselves . . . that doesn't mean she is not in pain [216]. [Toye 2015, mixed HCPs on a pain education course, unspecified HCP, UK]

> "I hate to say it . . . but I used to be one of the people that used to say, 'Oh, well, they are probably just wanting attention.' But I've changed in that matter. People are in pain, and it's not just to get attention." [167] [Clark 2006, age care facilities, unspecified HCP, USA]

Navigating juxtaposed models of medicine

This describes the challenge of navigating juxtaposed models of medicine: the biomedical and the bio-psychosocial. The biomedical model takes disease to be an objective biomedical category not influenced by psychosocial factors, whereas the bio-psychosocial model incorporates psychosocial factors influencing the pain experience [221]. There was a culturally entrenched pull towards the biomedical *siren song* of diagnosis[144].

> Being able to track something gives me more comfort than going by what you're telling me . . . because I like to see proof . . . You [want to] . . . be convinced that you're treating *something* and that what you're treating is real [158]. [Berg 2009, opioid prescription, physician, USA]

> I will listen to their story, I will examine them and I always say you have got to exclude the physical first that is your job . . . we have an obligation to exclude the physical first and not jump into [psychosocial explanations] because it reduces the patient to being an un-necessary complainer and I don't believe that they really are [145] [Wainwright 2006, primary care, GP, UK]

The following GP describes how chronic pain can obscure 'real' tangible health problems (such as high cholesterol) with the implication that chronic pain is less real:

> They don't seem to worry about issues that might be real . . . like his cholesterol is high . . . there are some other issues that he needs to attend to . . . his father died when he was fifty two. He's not worried [159]. [Bergman 2013, primary care, GP, USA]

For peer review only - http://bmjopen.bmj.com/site/about/guidelines.xhtml

Some HCPs utilised a dualistic bio-psychosocial model, whereby once *something* biomedical has been excluded, they made an abrupt shift towards psychosocial explanations. Here clinical work shifted away from diagnosis towards persuading patients that psychosocial factors influenced pain. This abrupt shift could threaten the therapeutic relationship and HCPs described how psychological explanations came with a stigma attached.

> The terminology . . . psychiatric and psychological . . . have a stigma attached to them that is not intended . . . we accept that patients with long term pain will have a psychological component to it but actually labelling it as that [216] [Toye 2015, mixed HCPs on a pain education course, unspecified HCP, UK]

HCPs therefore tended to default to physical explanations or used 'bridging' strategies to keep the patient on board. The following HCPs described the importance of approaching psychological explanations in a very careful way:

> It is a subtlety and if you present [the explanation for pain] as a completely airy fairy psychological, it is up to you, then they are going to go away dissatisfied, so you have got to lead them in gently [145] [Wainwright 2006, primary care, GP, UK]

> If you start from the body and if you ask a little carefully how things work when you are physically like that, then it is not threatening, and you can approach things, like, through the body [146] [Afrell 2010, pain specialist, physiotherapist, Norway]

Some felt that a diagnosis could help a patient to move forward, or give a sense of relief. One HCP described how they might 'feign diagnostic certainty' to achieve this goal [145]. Another HCP voiced ethical concerns about the deception of *feigning* diagnostic certainty.

> I think giving it a label that actually has no justification I think is misleading to the patient and I actually feel quite strongly about that [145] [Wainwright 2006, primary care, GP, UK]

Not all HCPs utilised a dualistic bio-psychosocial model and did not make this abrupt shift towards psychosocial explanations. Rather, they utilised an embodied bio-psychosocial approach with 'no breaking point where the physical becomes psychological'[157]. There was a sense that pain is multidimensional and that the 'physician gaze' [222] multifocal. Here clinical work involved understanding person's suffering from the outset of care. The following HCPs working in specialist pain services describe feelings of empathy and understanding:

For peer review only - http://bmjopen.bmj.com/site/about/guidelines.xhtml

> While we talked . . . many losses came up and I began myself to think about what all this was about in fact, what is this pain? Where it came out that there was a lot of disappointment, where there was divorce and . . . yes, it can't be purely physiological [146] [Afrell 2010, pain specialist, physiotherapist, Norway]

> Once a person's life has fallen apart it's not so much about the pain and the injury anymore. It's about all these other things in their life and it's all these other things that need to be addressed in order to get them better and get them back on track [164]. [Cartmill 2011, chronic pain services, unspecified HCP, Canada]

Those who utilised a more embodied psychosocial model recognised that sitting alongside and supporting patients, rather trying to 'fix' them, could be rewarding for both the patient and their HCP.

> I think the sort of traditional model of treatment doesn't allow people to express how pain has affected their whole life, it is very homed in to the particular area of the body and trying to fix it, and I just find it more satisfying to work in a way that acknowledges and discusses the impact[207] [Scott-Dempster 2014, pain specialist, physiotherapist, UK]

HCPs also described how time restrictions could encourage HCPs to focus on the physical body and were perceived as a barrier to an embodied approach.

> We are limited by the amount of time with the patient. I know this sounds bad, but [talking about pain] opens a can of worms[210]. [Siedleki 2014, acute care, nursing, USA]

Navigating the geography between patient and clinician

This describes the complexity of navigating the geography between patient and HCP. The metaphor of geography is used to portray a sense that the terrain could prove treacherous. The following HCP describes patients feeling dissatisfied by the health encounter:

> People feel let down by their doctors . . . The degree of satisfaction is very low . . . basically because we don't solve their problem . . . They go from one to the other, they find a doctor who gives them hope and they go to him [162]. [Briones-Vozmediano, Fibromyalgia, Occupational health doctor, Spain]

For peer review only - http://bmjopen.bmj.com/site/about/guidelines.xhtml

HCPs therefore made concessions in order to navigate the geography between patient and clinician. For example, they might make choices of doubtful medical utility, such as prescribing pain killers or referring for an investigation, in order to maintain relationships. Concessions were sometimes necessary to balance long and short term gains. The following HCP describes referring a patient for a test in order to show the patient that they are listening to them:

> Sometimes patients refuse to believe that their condition cannot be treated . . . and insistently ask for a series of medical investigations that you, as a doctor, would not perform. In such cases, a medical investigation can work as a therapy because it . . . shows that you listened to them [220]. [Zanini 2014, chronic pain services, neurology physician, USA]

HCPs also described the personal challenge that accompanied a need to balance professional expertise and patient empowerment. The following HCPs described how they could find it difficult to stand back and let patients make what they felt was a *'wrong'* decision. The following examples highlight HCPs personal struggle with this challenge:

> Trying to allow myself to listen objectively and to . . . sit with the fact that actually [the patient] might want to do something which is wholly *unsensible*, but allowing that to happen if that truly is what they want [207][Scott-Dempster 2014, pain specialist, physiotherapist, UK]

> I recognise that . . . we are trying to promote learning by giving choice and allowing people to get it wrong . . . we learn by *doing* not by being told what to do. I get that, although it is still hard . . . not to give advice when I see . . . that the advice can be really helpful[155] Barker 2015, pain specialist, physiotherapist, UK]

If a conflict arose, the 'short-circuit' [217] could be to take control, but there remained a sense that discussion rather than enforcement was more effective in the long-term. Empowering patients involved helping them to make decisions for themselves with HCP support.

> [If we think] 'I know that this is the correct answer' . . . then you do not allow the patient to participate. He then becomes a receiver. But if you share your knowledge . . . then you offer the patient an opportunity to think and decide by himself [217] [Tveiten 2009, chronic pain

For peer review only - http://bmjopen.bmj.com/site/about/guidelines.xhtml

services, unspecified HCP, Norway]

Patients have to embrace our suggestion because they are convinced that it is the right one and not because we want them to choose a particular option. If you propose something that is inconsistent with their experience or knowledge, there is a risk that they will not listen to you [220] [Zanini 2014, chronic pain services, rheumatology physician, USA]

The challenge of dual advocacy

This theme describes the HCP as being simultaneously an advocate of the patient and an advocate of the healthcare system. Whilst representing the patients' interests, at the same time, HCPs represented the healthcare system and made important decisions as representatives of that system. This resonates with the challenge of making decisions based on what is best for the individual patient alongside utilitarian decisions for the greatest number. At times this sense of dual advocacy could create an uncomfortable feeling that healthcare colleagues were not working on the same side as each other or the patient. At times, it could feel like the experience was spiralling of control ('a ship without a rudder')

It all ends up on our doorstep. It is not only we who face the system – we are mediators of sorts between the patient and the system. Not only must we work with the patient against the system, but with the system as well [170].[Dahan 2007, guidelines, GP, Israel]

As soon as someone gets sort of uncomfortable they will shift to a different prescriber and they will push them along a certain course . . . . and I honestly think it's like a ship without a rudder and it's just going round and round in circles[196].[McCrorie 2015, opioid prescription, GP, UK]

HCPs also described how it could prove difficult to access specialist pain services, and that it could feel like there was a mismatch between what the HCP expected and what they received.

There is a really big access issue with the pain clinics right now . . . while I can refer them, their likelihood of getting an appointment, even with strong advocacy from me, is very low . . . Often I find that they are not accomplishing any more than I was and [patients] are often sent back to me with them essentially saying, 'we did our best.' It's very frustrating, because if they were easy . . . they wouldn't have been referred.[156] [Barry 2010, opioid prescription, physician, USA]

For peer review only - http://bmjopen.bmj.com/site/about/guidelines.xhtml

HPCs recognised the benefits of a healthcare system where the cogs worked smoothly: the benefits of reciprocity and collaboration, being confident in the capabilities of colleagues and reciprocal respect.

> We get a lot of mileage about slapping each other on the back a little bit. And increasing other members of the team's confidence by respecting other members of the team, their profile is improved [185].[Howarth 2012, chronic pain services, physician, UK]

> If the team sort of echoes the same message and provides richness in terms of their different perspective on it . . . then I think there's less confusion for the poor clients and they're able to follow through on a unified evidence-based recommendation [164]. [Cartmill 2011, chronic pain services, unspecified HCP, Canada]

Personal costs

This theme describes the emotional costs of treating patients with chronic pain. First, the biomedical model could create a sense of professional failure for not being able to *fix*; 'how did we fail them?' [210]. This sense of failure could be demoralising and undermine HCPs sense of professionalism.

> You become a doctor not to tell people I can't do anything, I can't find anything, you have this perception of yourself as well that you're going to sort it out and if you can't sort it out, it's frustrating. What's the point of you being there? [201] [Patel 2008, primary care, GP, UK]

> It's awful, and I think it's demoralizing when you leave people in pain. That's just so disrespectful. I mean you're supposed to be a doctor, you're supposed to relieve pain and suffering, and you ignore the pain [156].[Barry 2010, opioid prescription, physician, USA]

However, an embodied bio-psychosocial approach which hinged on recognising human losses could incur a deep sense of personal loss. HCPs described how they had to manage the tension between proximity and distance.

> We forget how much chronic pain affects the patient. They lose their jobs, they have emotional stress and depression and the depression itself is a big loss of productivity to the patient but also to the entire family and to the community[210] [Siedleki 2014, acute care, nurse, USA]

For peer review only - http://bmjopen.bmj.com/site/about/guidelines.xhtml

> Trying to listen to the person . . . sort of empathise . . . . [but] almost protected professionally . . . trying to see where that person was coming from but not letting it become too personal . . . I've used the phrase detached empathy [216]. [Toye 2015, mixed HCPs on a pain education course, unspecified HCP, UK]

The craft of pain management

This describes clinical work as an experience-based competence or 'craft' [171] gained from experience rather than didactic education. At times HCPs felt under-skilled in chronic pain management.

> The problem is, we don't know how to treat pain. And so everybody is telling me I'm not treating pain well, but nobody is helping me figure out how to treat the pain[213]. [Spitz 2011, opioid prescription, physician, USA]

Personal experience or maturity, amount of experience treating patients with chronic pain and learning from colleagues underpinned craft knowledge.

> One becomes more stable as a person [with age], and does not really have the same demands and does not believe that one can do everything, that one is able to solve everything . . . Young doctors can have in them, that they believe that they will solve everything[153]. [Asbring 2003, fibromyalgia, physician, USA]

> New grads can't learn all of this, they need a certain number of years, you can't teach them all of this[144].[Slade 2012, physiotherapists, Australia]

Although some HCPs felt that clinical guidelines could support a more patient-centred approach [179, 204], there was a stronger sense that they constrained craft knowledge. HCPs therefore used guidelines pragmatically within the remit of their own knowledge.

> Treatment has to be tailored to patient's needs and prescriptive guidelines promoting "one size fits all" is not acceptable[143]. [Wilson 2014, guidelines, unspecified HCP, UK]

For peer review only - http://bmjopen.bmj.com/site/about/guidelines.xhtml

> If you work according to the guidelines, you are constrained in your performance . . . what would be left of your independence, your own competence, your own practical experience . . . Am I to conclude then that my training was useless. . . I'm free to take or leave these things, to look at whether they suit my own ideas of how to approach my patients[179]. [Harting 2009, guidelines, physiotherapist, Netherlands]

Line of Argument

The final phase of meta-ethnographic analysis is to develop a model or *line of argument* that is abstracted from, *but more than the sum of*, the themes (figure 2) [11]. Through discussion with each other, and the advisory group, the reviewers developed a model which made sense of the final themes. The model is underpinned by a series of tensions that can help us to understand and reflect on the experience of treating patients with chronic non-malignant pain : (a) between a dualistic biomedical model and an embodied psychosocial model; (b) between professional distance and proximity; (c) between professional expertise and patient empowerment (d) between a need to make concessions in order to maintain relationships and known biomedical utility (e) between patient and healthcare system advocacy. We conceptualise these tensions, on a mixing console[1], as underpinning the craft of chronic non-malignant pain management. The poles are neither inherently *good* nor *bad*; just as bass and treble are neither inherently good nor bad. It is the correct mix within a context that contributes to the quality of music. The levels indicated in figure 2 are an example and do not indicate any sense of 'correct' balance. Different HCPs may adjust the balance differently for each individual and context. Our console also incorporates the *pitch* or level of loss, both professional and personal, that can contribute to the harmony or dissonance of a therapeutic encounter. The siren song of diagnosis, reflecting the cultural pull of the biomedical model, is also shown as a factor that can have an important impact on the balance between poles.

Discussion

Our innovation is to present the first internationally relevant QES of healthcare professionals' experiences of treating chronic non-malignant pain. Already we know that, from the patient perspective, this experience can be adversarial [10]. Patients with chronic pain struggle to affirm their sense of self; their present and future appears unpredictable; they search for a credible explanation for their pain; they do not always feel heard, believed or valued by healthcare

[1] Idea for image of a mixing console from Cathy Jenkins, OUH NHS Foundation Trust, Oxford

For peer review only - http://bmjopen.bmj.com/site/about/guidelines.xhtml

professionals; they struggle to prove themselves in the face of scepticism. We present our line of argument as a mixing console that can help healthcare professionals to understand, think about and modify their experience of treating patients with chronic pain. For example, A HCP could consider: am I making a sudden shift to psychosocial explanations when I can find nothing biomedical or am I considering psychosocial factors alongside medical investigations; do I understand this patient's experience or am I too distant; have I discussed and negotiated the various options or am I trying to instruct and enforce; am I considering medical utility or am I making a concession (and is this concession for my benefit or my patient's benefit); am I effectively balancing my role as dual advocate? Beyond these dualities, our model encourages HCPs to consider the personal impact of treating patients with chronic non-malignant pain. How often do you find yourself wondering whether you have failed as a professional? (professional loss) Are you feeling *bombarded by despair*? (personal loss). If the answer is yes to either of these, what measures are there in place to tackle this?

Culture has been described as the 'inherited lens' through which individuals understand the world and learn how to live in it [223]. Both patients and their HCPs are embedded in a wider culture where biomedical explanations have the power to bestow credibility. The studies included explore the experience of both diagnosing and treating pain and demonstrate that these are inextricably linked. Our findings highlight the cultural scepticism that underpins the *siren song of diagnosis*, where HCPs and patients can be driven by the need for a diagnosis. This may help us to understand why patients with chronic pain often experience a strong sense of not being believed. They also demonstrate how the bio-psychosocial model can hide a continuing dualism, where HCPs prioritise biomedical findings and make an abrupt switch to psychosocial explanations when no diagnosis is found. This abrupt shift may explain patients' feeling of lost credibility. A more embodied non-dualistic bio-psychosocial approach at the outset would help HCPs to support patients with chronic pain. Our findings also demonstrate the complexity of navigating the geography between patients and HCPs. In this borderland, HCPs sometimes make concessions that are not evidence-based in order to maintain effective relationships. These concessions have policy and practice implications, for example, in the context of recent USA [224]and UK [225] guidelines on opioid prescription for chronic non-malignant pain, it might help to explain why an increasing number of HCPs are prescribing opioids despite very limited evidence for long-term opioid therapy for chronic pain outside of end-of-life care [226]. Our findings also have educational implications: for example, navigating relationships requires skills to finely balance the tensions inherent in the model whilst managing potential personal and professional losses. HCPs included in this review did not discuss

For peer review only - http://bmjopen.bmj.com/site/about/guidelines.xhtml

their own personal life context which intuitively might contribute to a sense of loss. This might indicate that there were topics that were not explored in the initial interview studies and further research might explore the impact of this on HCPs' resilience to challenges of treating people with chronic pain and other conditions. HCPs described experience of treating chronic non-malignant pain that was not boundaried to a particular body system, but was a summative experience cutting across conditions. Further research might focus on specific diagnosis (such as neuropathic, visceral, pelvic or phantom pain, arthritis) in order to explore potential similarities and difference in HCP experiences of treating these conditions.

Although we utilised the GRADE CERQual approach, there is currently no agreed way to determine confidence in QES findings. It would be useful for future studies to consider the following issues: First, although GRADE CERQual considers methodological limitations as having an impact on confidence in reviews, there is limited agreement about what a good qualitative study is [29, 31]. Indeed, a significant number of QES reviewers choose not to appraise studies [24]. Although quality appraisal might highlight methodological flaws, it does not necessarily help us to appraise the usefulness of findings for the purposes of QES. It could be argued that good studies are excluded if our primary concern is methodology rather than conceptual insight [24, 31]. It would be useful for future studies to address how reviewers can be more discerning about the value of particular studies and the influence on analytical decision. This issue will become more important as the number of primary qualitative research studies grows. Although our reviewers agreed about which studies were 'key', 'fatally flawed' or 'irrelevant'[21] the majority of studies were appraised as 'satisfactory'. As only five studies were appraised as 'key' this status did not influence data analysis. Secondly, GRADE CERQual considers adequacy (weight) and coherence (consistency) of data as important factors that can contribute to confidence in a review finding. However do these necessarily equate to validity and how do we know *what* is adequate? The issue of determining adequacy resonates with the unresolved question 'how many qualitative interviews is enough?'[227]. We chose to rate our confidence in a finding as high when a theme was supported by a least half of the studies (n≥39). However, although you could argue that weight and consistency [32] of findings contribute to the persuasiveness of a finding, it is important to consider that a unique idea can exert a significant pull. It is thus important not to ignore unique or inconsistent findings. We have found that confidence in QES findings can grow when you incorporate a large number of studies. However, QES reviewers can be caught between a rock and a hard place as they face criticisms for undertaking reviews that are 'too small' (and thus anecdotal) or 'too large' (not in-depth). Another potential criticism of a QES that includes a large number of studies is that it is possible to lose sight of the nuances of the

For peer review only - http://bmjopen.bmj.com/site/about/guidelines.xhtml

primary studies. We found that using Nvivo qualitative analysis software allowed us to keep track of our analytical decisions whilst being able to continually refer back to the primary studies. This helped us to ensure that our findings remained grounded in these primary studies.

Findings from QES in health aim to provide ideas that can help to improve the experience of healthcare. We have presented a novel line of argument that helps us to understand, think about and modify our experience of diagnosing and treating patients with chronic non-malignant pain. Our line of argument may be transferable to other patient groups or situations. We conceptualise dualities, on a mixing console, as a useful way to frame the patient-clinician relationship. It would be useful for HCPs to consider their individual mix and contemplate a *re-mix* if necessary in order to successfully support people with chronic pain. Now we have a body of qualitative knowledge exploring patients' experiences of chronic pain[9] and healthcare professionals' experiences, the next challenge in practice is to bring these two bodies of knowledge together and look at how HCPs and patients can work together in managing pain.

For peer review only

For peer review only - http://bmjopen.bmj.com/site/about/guidelines.xhtml

References

1. IASP: **Classification of Chronic Pain**. Seattle, USA: International Association for the Study of Pain (IASP) Press; 1994.
2. Fayaz A, Croft P, Langford RM, Donaldson LJ, Jones GT: **Prevalence of chronic pain in the UK: a systematic review and meta-analysis of population studies**. *BMJ Open* 2016, **6**(6):http://bmjopen.bmj.com/content/6/6/e010364.
3. Donaldson L: **2009 Annual Report of the Chief Medical Office (Department of Health**. In. http://www.sthc.co.uk/Documents/CMO_Report_2009.pdf [accessed 11 Sept 12]; 2009.
4. Breivik H, Collett B, Ventafridda V, Cohen R, Gallacher D: **Survey of chronic pain in Europe: Prevalence, impact on daily life, and treatment**. *European Journal of Pain* 2006, **10**(4):287-333.
5. Nahin R: **Estimates of Pain Prevalence and Severity in Adults: United States, 2012**. *Journal of Pain* 2015, **16**(8):769-780.
6. Price C, Hoggart B, Olukoga O, CdeC-Williams A, Bottle A: **National Pain Audit**: British Pain Society; 2012.
7. Briggs E, Carr E, Whittaker M: **Survey of undergraduate pain curricula for healthcare professionals in the United Kingdom**. *European Journal of Pain* 2011, **15**(8):789–795.
8. Noyes J, Popay J, Pearson A, Hannes K, Booth A: **Qualitative Research and Cochrane Reviews**. In: *Cochrane Handbook for Systematic Reviews.* edn. Edited by Higgins J, Green S. Chichester, UK: John Wiley & Sons; 2008.
9. Toye F, Seers K, Hannink E, Barker K: **A mega-ethnography of eleven qualitative evidence syntheses exploring the experience of living with chronic non-malignant pain**. *BMC Medical Research Methodology* 2017, **17**(116):https://bmcmedresmethodol.biomedcentral.com/track/pdf/10.1186/s12874-12017-10392-12877?site=bmcmedresmethodol.biomedcentral.com.
10. Toye F, Seers K, Allcock N, Briggs M, Carr E, Andrews J, Barker K: **A meta-ethnography of patients' experiences of chronic non-malignant musculoskeletal pain**. *Health Services and Delivery Research* 2013, **1**(12):1-189.
11. Noblit G, Hare R: **Meta-ethnography: Synthesising Qualitative Studies**. California: Sage Publications; 1988.
12. Britten N, Campbell R, Pope C, Donnovan J, Morgan M, Pill R: **Using meta ethnography to synthesise qualitative research: a worked example**. *Journal of Health Services and Research Policy* 2002, **7**(4):209-215.
13. Campbell R, Pound P, Pope C, Britten N, Pill R, Morgan M, Donovan J: **Evaluating meta-ethnography: a synthesis of qualitative research on lay experience of diabetes and diabetes care**. *Social Science & Medicine* 2003, **56**:671-684.
14. Malpass A, Shaw A, Sharp D, Walter F, Feder G, Ridd M, Kessler D: **"Medication career" or "Moral career"? The two sides of managing anti-dperessants: A meta-ethnography of patients experience of antidepressants**. *Social Science and Medicine* 2009, **68**:154-168.
15. Barker K, Toye F, MinnsLowe C: **A qualitative systematic review of patients' experience of osteoporosis using meta-ethnography**. *Archives of Osteoporosis* 2016, **11**(33):DOI 10.1007/s11657-11016-10286-z.
16. Toye F, Seers K, Allcock N, Briggs M, Carr E, Barker K: **A synthesis of qualitative research exploring the barriers to staying in work with chronic musculoskeletal pain**. *Disability & Rehabilitation* 2016, **38**(6):566-572.
17. Toye F, Seers K, Barker K: **A meta-ethnography of patients' experiences of chronic pelvic pain: struggling to construct chronic pelvic pain as 'real'**. *Journal of Advanced Nursing* 2014, **70**(12):2713-2727.

For peer review only - http://bmjopen.bmj.com/site/about/guidelines.xhtml
For peer review only

18. Toye F, Seers K, Allcock N, Briggs M, Carr E, Barker K: **Meta-ethnography 25 years on: challenges and insights for synthesising a large number of qualitative studies**. *BMC Medical Research Methodology* 2014, **14**(80).
19. Sandelowski M, Barrosso J: **Handbook for synthesising qualitative research**. New York: Springer Publishing Company; 2007.
20. Dixon-Woods M, Booth A, Sutton A: **Synthesizing qualitative research: a review of published reports**. *Qualitative Research* 2007, **7**:375-422.
21. Dixon-Woods M, Agarwal S, Jones D, Young B, Sutton A: **Synthesising qualitative and quantitative research evidence: a review of possible methods**. *Journal of Health Services and Research Policy* 2005, **10**(1):45-53.
22. Hannes K, Macaitis K: **A move to more systematic and transparent approaches in qualitative evidence synthesis: update on a review of published papers**. *Qualitative Research* 2012, **12** (4):402-442
23. Barnett-Page E, Thomas J: **Methods for synthesis of qualitative research: a critical review**. *Economic and Social Research Council Research Methods* 2009, **National Centre for Research Methods Working Paper Series**(01/09).
24. Campbell R, Pound P, Morgan M, Daker-White G, Britten N, Pill R, Yardley L, Pope C, Donovan J: **Evaluating meta-ethnography: systematic analysis and synthesis of qualitative research**. *Health Technology Assessment* 2011, **15**(43).
25. Wong SS WN, Haynes RB. : **Developing optimal search strategies for detecting clinically relevant qualitative studies in MEDLINE**. *Medinfo* 2004, **11**(1):311-316.
26. Wilczynski NL MS, Haynes RB. : **Search strategies for identifying qualitative studies in CINAHL**. *Qualitative Health Research* 2007, **17**(5):705-710.
27. McKibbon KA WN, Haynes RB. : **Developing optimal search strategies for retrieving qualitative studies in PsycINFO**. *Evaluation & the Health Professions* 2006, **29**(4):440-454.
28. Walters LA WN, Haynes RB; Hedges Team. : **Developing optimal search strategies for retrieving clinically relevant qualitative studies in EMBASE**. *Qualitative Health Research* 2006, **16**(1):162-168.
29. Dixon-Woods M, Sutton A, Shaw R, Miller T, Smith J, Young B, Bonas S, Booth A, Jones D: **Appraising qualitative research for inclusion in systematic reviews: a quantitative and qualitative comparison of three methods**. *Journal of Health Services and Research Policy* 2007, **12**(1):42-47.
30. CASP: **Critical Appraisal Skills Programme: making sense of evidence about clinical effectiveness: 10 questions to help you make sense of qualitative research**. *http://wwwcasp-uknet/wp-content/uploads/2011/11/CASP_Qualitative_Appraisal_Checklist_14oct10pdf* 2010.
31. Toye F, Seers K, Allcock N, Briggs M, Carr E, Andrews J, Barker K: **'Trying to pin down jelly' - exploring intuitive processes in quality assessment for meta-ethnography**. *BMC Medical Research Methodology* 2013, **13**:46.
32. Lewin S, Glenton C, Munthe-Kaas H, Carlsen B, Colvin CJ, Gülmezoglu M, Noyes J, Booth A, Garside R, Rashidian A: **Using Qualitative Evidence in Decision Making for Health and Social Interventions: An Approach to Assess Confidence in Findings from Qualitative Evidence Syntheses (GRADE-CERQual)**. *PLOS Medicine* 2016, **12**(10):e1001895. doi:1001810.1001371/journal. pmed.1001895.
33. Pope C, Mays N, Popay J: **Synthesizing Qualitative and Quantitative Health Research: A Guide to Methods**. Berkshire, UK: Open University Press; 2007.
34. Nvivo: **Nvivo qualitatitve data analysis and software, QSR International Pty Ltd.** In: *software for qualitative data analysis.* 9 edn; 2010.
35. Frost J, Garside R, Cooper C, Britten N: **Meta-study as diagnostic: toward content over form in qualitative synthesis**. *Qualitative Health Research* 2016, **26**(3):307-3019.

For peer review only - http://bmjopen.bmj.com/site/about/guidelines.xhtml

36. Chancellor J, Martin M, Liedgens H, Baker G, Müller-Schwefe HH: **Stated preferences of physicians and chronic pain sufferers in the use of classic strong opioids**. *Value in health : the journal of the International Society for Pharmacoeconomics and Outcomes Research* 2012, **15**(1):106.
37. Coutu MF, Legare F, Stacey D, Durand MJ, Corbiere M, Bainbridge L, Labrecque ME: **Occupational therapists' shared decision-making behaviors with patients having persistent pain in a work rehabilitation context: A cross-sectional study**. *Patient Education and Counseling* 2015, **98**(7):864-870.
38. Al-Shaer D, Hill PD, Anderson MA: **Nurses' knowledge and attitudes regarding pain assessment and intervention**. *Medsurg nursing : official journal of the Academy of Medical-Surgical Nurses* 2011, **20**(1):7-11.
39. Cedraschi C, Robert J, Perrin E, Fischer W, Goerg D, Vischer TL: **The role of congruence between patient and therapist in chronic low back pain patients**. *Journal of Manipulative and Physiological Therapeutics* 1996, **19**(4):244-249.
40. Cedraschi C, Robert J, Goerg D, Perrin E, Fischer W, Vischer TL: **Is chronic non-specific low back pain chronic? Definitions of a problem and problems of a definition**. *The British journal of general practice : the journal of the Royal College of General Practitioners* 1999, **49**(442):358.
41. Ferrell B: **Ethical perspectives on pain and suffering**. *Pain management nursing : official journal of the American Society of Pain Management Nurses* 2005, **6**(3):83.
42. Gillmore R, Hill T: **Reactions to patients who complain of pain: Effects of ambiguous diagnosis**. *Journal of Applied Social Psychology* 1981, **11**(1):14.
43. Haq I, Fuller J, Dacre J: **The use of patient partners with back pain to teach undergraduate medical students**. *Rheumatology* 2006, **45**(4):430-434.
44. Kotarba A: **Perceptions of death, belief systems and the process of coping with chronic pain**. *Social Science & Medicine* 1983, **17**(10):681.
45. Sloan TJ, Walsh DA: **Explanatory and diagnostic labels and perceived prognosis in chronic low back pain**. *Spine* 2010, **35**(21):E1120-NaN.
46. Valjakka AL, Salantera S, Laitila A, Julkunen J, Hagelberg NM: **The association between physicians' attitudes to psychosocial aspects of low back pain and reported clinical behaviour: A complex issue**. *Scandinavian Journal of Pain* 2013, **4**(1):25-30.
47. Walker JM, Akinsanya JA, Davis BD, Marcer D: **The nursing management of elderly patients with pain in the community: study and recommendations**. *Journal of Advanced Nursing* 1990, **15**(10):1154-1162.
48. Kaasalainen S, Agarwal G, Dolovich L, Brazil K, Papaioannou A: **Managing pain medications in long-term care: nurses' views**. *British Journal of Nursing* 2015, **24**(9):484-490.
49. Parsons A, Mamdani M, Bhattacharyya O, Fortin CM, Melo M, Salmon C, Raptis R, Bain D, O'Campo P: **Narcotic analgesic utilization amongst injured workers: using concept mapping to understand current issues from the perspectives of physicians and pharmacists**. *BMC health services research* 2011, **11**:280.
50. Tousignant-Laflamme Y, Tousignant M, Lussier D, Lebel P, Savoie M, Lalonde L, Choiniere M: **Educational needs of health care providers working in long-term care facilities with regard to pain management**. *Pain Research and Management* 2012, **17**(5):341-346.
51. Walker JM: **Caring for elderly people with persistent pain in the community: a qualitative perspective on the attitudes of patients and nurses**. *Health & Social Care in the Community* 1994, **2**(4):221-229.
52. Askew R, Kibelstis C, Overbaugh S, Walker S, Nixon-Cave K, Shepard KF: **Physical therapists' perception of patients' pain and its effect on management**. *Physiotherapy Research International* 1998, **3**(1):37-58.

For peer review only - http://bmjopen.bmj.com/site/about/guidelines.xhtml

53. Baker C, Gallois C, Driedger SM, Santesso N: **Communication accommodation and managing musculoskeletal disorders: Doctors' and patients' perspectives**. *Health Communication* 2011, **26**(4):379.
54. Barker KL, Reid M, Lowe CJM: **Divided by a lack of common language? - A qualitative study exploring the use of language by health professionals treating back pain**. *BMC Musculoskeletal Disorders* 2009, **10**(1).
55. Beattie A, Shaw A, Yardley L, Little P, Sharp D: **Participating in and delivering the ATEAM trial (Alexander technique lessons, exercise, and massage) interventions for chronic back pain: A qualitative study of professional perspectives**. *Complementary Therapies in Medicine* 2010, **18**(3-4):119-127.
56. Billis V, McCarthy J, Stathopoulos I, Kapreli E, Pantzou P, Oldham A: **The clinical and cultural factors in classifying low back pain patients within Greece: A qualitative exploration of Greek health professionals**. *Journal of evaluation in clinical practice* 2007, **13**(3):337.
57. Bjorner T, Kjolsrod L: **How GPs understand patients' stories: A qualitative study of benzodiazepine and minor opiate prescribing in Norway**. *European Journal of General Practice* 2002, **8**(1):25-30.
58. Blondal K, Halldorsdottir S: **The challenge of caring for patients in pain: from the nurse's perspective**. *Journal of Clinical Nursing* 2009, **18**(20):2897-2907.
59. Breen A, Austin H, Campion-Smith C, Carr E, Mann E: **"You feel so hopeless": A qualitative study of GP management of acute back pain**. *European Journal of Pain* 2007, **11**(1):21-29.
60. Buchbinder M, Wilbur R, McLean S, Sleath B: **"Is there any way I can get something for my pain?" Patient strategies for requesting analgesics**. *Patient Education and Counseling* 2015, **98**(2):137-143.
61. Buijs PC, Lambeek LC, Koppenrade V, Hooftman WE, Anema JR: **Can workers with chronic back pain shift from pain elimination to function restore at work? Qualitative evaluation of an innovative work related multidisciplinary programme**. *Journal of Back and Musculoskeletal Rehabilitation* 2009, **22**(2):65-73.
62. Bullington J, Nordemar R, Nordemar K, Sjöström-Flanagan C: **Meaning out of chaos: a way to understand chronic pain**. *Scandinavian Journal of Caring Sciences* 2003, **17**(4):325-332.
63. Bussieres AE, Patey AM, Francis JJ, Sales AE, Grimshaw JM, Canada PT, Brouwers M, Godin G, Hux J, Johnston M *et al*: **Identifying factors likely to influence compliance with diagnostic imaging guideline recommendations for spine disorders among chiropractors in North America: a focus group study using the Theoretical Domains Framework**. *Implementation science : IS* 2012, **7**.
64. Carr CJ, Worswick L, Wilcock M, Campion-Smith C, Hettinga D: **Improving services for back pain: putting the patient at the centre of interprofessional education**. *Quality in primary care* 2012, **20**(5):345.
65. Corrigan C, Desnick L, Marshall S, Bentov N, Rosenblatt A: **What can we learn from first-year medical students' perceptions of pain in the primary care setting?** *Pain medicine (Malden, Mass)* 2011, **12**(8):1216.
66. Costa-Black KM, Durand M, Imbeau D, Baril R, Loisel P: **Interdisciplinary team discussion on work environment issues related to low back disability: a multiple case study**. *Work* 2007, **28**(3):249-266.
67. Cruz B, Moore A, Cross V: **Clinical reasoning and patient-centred care in musculoskeletal physiotherapy in Portugal--a qualitative study**. *Manual therapy* 2012, **17**(3):246.
68. Dean G, Smith A, Payne S, Weinman J: **Managing time: an interpretative phenomenological analysis of patients' and physiotherapists' perceptions of adherence to therapeutic exercise for low back pain**. *Disability and rehabilitation* 2005, **27**(11):625.
69. Donovan J: **Learning disability nurses' experiences of being with clients who may be in pain**. *Journal of advanced nursing* 2002, **38**(5):458.

For peer review only - http://bmjopen.bmj.com/site/about/guidelines.xhtml

70. Doran J: **Experiencing Wellness Within Illness: Exploring a Mindfulness-Based Approach to Chronic Back Pain**. *Qualitative Health Research* 2014, **24**(6):749-761.
71. Droes NS: **Role of the nurse practitioner in managing patients with pain**. *Internet Journal of Advanced Nursing Practice* 2004, **6**(2):12p-NaN.
72. Dysvik E, Furnes B: **Nursing leadership in a chronic pain management group approach**. *Journal of nursing management* 2012, **20**(2):187.
73. Eifried S: **Bearing witness to suffering: the lived experience of nursing students**. *The Journal of nursing education* 2003, **42**(2):59.
74. Elder N, Ricer R, Tobias B: **How respected family physicians manage difficult patient encounters**. *Journal of the American Board of Family Medicine : JABFM* 2006, **19**(6):533.
75. Espeland A, Baerheim A: **General practitioners' views on radiology reports of plain radiography for back pain**. *Scandinavian Journal of Primary Health Care* 2007, **25**(1):15-20.
76. Francke AL, Lemmens ABT, Abu-Saad HH, Grypdonck M: **Nurses' perceptions of factors influencing the use of a pain program**. *Journal of Pain & Symptom Management* 1997, **14**(5):300-311.
77. Gunby SS: **The lived experience of nursing students in caring for suffering individuals**. *Holistic nursing practice* 1996, **10**(3):63.
78. Haskins R, Osmotherly G, Southgate E, Rivett A: **Physiotherapists' knowledge, attitudes and practices regarding clinical prediction rules for low back pain**. *Manual therapy* 2014, **19**(2):142.
79. Hildebran C, Cohen J, Irvine M, Foley C, O'Kane N, Beran T, Deyo A: **How clinicians use prescription drug monitoring programs: a qualitative inquiry**. *Pain medicine (Malden, Mass)* 2014, **15**(7):1179.
80. Hooten WM, Vickers S, Shi Y, Ebnet L, Townsend O, Patten A, Warner O: **Smoking cessation and chronic pain: Patient and pain medicine physician attitudes**. *Pain Practice* 2011, **11**(6):552.
81. Howarth M, Warne T, Haigh C: **Pain from the inside: understanding the theoretical underpinning of person-centered care delivered by pain teams**. *Pain management nursing : official journal of the American Society of Pain Management Nurses* 2014, **15**(1):340-348.
82. Jeffrey E, Foster E: **A qualitative investigation of physical therapists' experiences and feelings of managing patients with nonspecific low back pain**. *Physical therapy* 2012, **92**(2):266.
83. Josephson I, Bulow P, Hedberg B: **Physiotherapists' clinical reasoning about patients with non-specific low back pain, as described by the International Classification of Functioning, Disability and Health**. *Disability and Rehabilitation* 2011, **33**(23-24):2217-2228.
84. Josephson I, Hedberg B, Bulow P: **Problem-solving in physiotherapy--physiotherapists' talk about encounters with patients with non-specific low back pain**. *Disability and Rehabilitation* 2013, **35**(8):668-677.
85. Karstens S, Joos S, Hill JC, Krug K, Szecsenyi J, Steinhauser J: **General practitioners views of implementing a stratified treatment approach for low back pain in Germany: A qualitative study**. *PLoS ONE* 2015, **10**(8).
86. Kee CC, Epps CD: **Pain management practices of nurses caring for older patients with osteoarthritis**. *Western Journal of Nursing Research* 2001, **23**(2):195-211.
87. Ladak SJ, McPhee C, Muscat M, Robinson S, Kastanias P, Snaith K, Elkhouri M, Shobbrook C: **The journey of the pain resource nurse in improving pain management practices: Understanding role implementation**. *Pain Management Nursing* 2013, **14**(2):68.
88. Lærum E, Indahl A, Skouen JS: **What is 'the good back-consultation'? A combined qualitative and quantitative study of chronic low back pain patients' interaction with and perceptions of consultations with specialists**. *Journal of Rehabilitation Medicine (Taylor & Francis Ltd)* 2006, **38**(4):255-263.

For peer review only

For peer review only - http://bmjopen.bmj.com/site/about/guidelines.xhtml

89. Larsson H, Blomqvist K: **From a diagnostic and particular approach to a person-centred approach: a development project**. *Journal of Clinical Nursing* 2015, **24**(3/4):465-475.
90. Mackintosh-Franklin C: **The impact of experience on undergraduate preregistration student nurses' responses to patients in pain: A 2-year qualitative longitudinal study**. *Pain Management Nursing* 2014, **15**(1):199.
91. Magnusson JE, Fennell JA: **Understanding the role of culture in pain: Maori practitioner perspectives relating to the experience of pain**. *New Zealand Medical Journal* 2011, **124**(1328):41-51.
92. Manias E: **Complexities of pain assessment and management in hospitalised older people: A qualitative observation and interview study**. *International Journal of Nursing Studies* 2012, **49**(10):1243-1255.
93. May C, Allison G, Chapple A, Chew-Graham C, Dixon C, Gask L, Graham R, Rogers A, Roland M: **Framing the doctor-patient relationship in chronic illness: A comparative study of general practitioners' accounts**. *Sociology of health & illness* 2004, **26**(2):135.
94. May S, Greasley A, Reeve S, Withers S: **Expert therapists use specific clinical reasoning processes in the assessment and management of patients with shoulder pain: a qualitative study**. *The Australian journal of physiotherapy* 2008, **54**(4):261-266.
95. McDonald DD: **Trialing to Pain Control: A Grounded Theory**. *Research in Nursing & Health* 2014, **37**(2):107-117.
96. McGee J, Kaylor D, Emmott H, Christopher J: **Defining chronic pain ethics**. *Pain Medicine* 2011, **12**(9):1376.
97. McIntosh A, Shaw FM: **Barriers to patient information provision in primary care: patients' and general practitioners' experiences and expectations of information for low back pain**. *Health expectations : an international journal of public participation in health care and health policy* 2003, **6**(1):19.
98. Miller WL, Yanoshik MK, Crabtree BF, Reymond WK: **Patients, family physicians, and pain: visions from interview narratives**. *Family medicine* 1994, **26**(3):179.
99. Miller S, Pinnington A: **Straightforward consultation or complicated condition? General practitioners' perceptions of low back pain**. *The European journal of general practice* 2003, **9**(1):3.
100. Montali L, Monica C, Riva P, Cipriani R: **Conflicting representations of pain: A qualitative analysis of health care professionals' discourse**. *Pain Medicine* 2011, **12**(11):1585.
101. Nielsen M, Keefe J, Bennell K, Jull A: **Physical Therapist-Delivered Cognitive-Behavioral Therapy: A Qualitative Study of Physical Therapists' Perceptions and Experiences**. *Physical Therapy* 2014, **94**(2):197-210.
102. Noll E, Key A, Jensen G: **Clinical reasoning of an experienced physiotherapist: insight into clinician decision-making regarding low back pain**. *Physiotherapy Research International* 2001, **6**(1):40-52.
103. Oien AM, Iversen S, Stensland P: **Narratives of embodied experiences - Therapy processes in Norwegian psychomotor physiotherapy**. *Advances in Physiotherapy* 2007, **9**(1):31-39.
104. Oien AM, Råheim M, Iversen S, Steihaug S: **Self-perception as embodied knowledge -- changing processes for patients with chronic pain**. *Advances in Physiotherapy* 2009, **11**(3):121-130.
105. Ong BN, Hooper H: **Involving users in low back pain research**. *Health Expectations* 2003, **6**(4):332-341.
106. Ong BN, Hooper H: **Comparing clinical and lay accounts of the diagnosis and treatment of back pain**. *Sociology of Health & Illness* 2006, **28**(2):203-223.
107. Ottenheijm PG, Hesselmans JJM, Kemper A, Moser A, de AR, Dinant G-J, Cals WL: **GPs' perspectives on the diagnostic work-up in patients with shoulder pain: a qualitative study**. *Journal of evaluation in clinical practice* 2014, **20**(3):239.

For peer review only - http://bmjopen.bmj.com/site/about/guidelines.xhtml

108. Parke B: **Gerontological nurses' ways of knowing. Realizing the presence of pain in cognitively impaired older adults**. *Journal of gerontological nursing* 1998, **24**(6):21.
109. Peiris D, Williams C, Holbrook R, Lindner R, Reeve J, Das A, Maher C: **A Web-based clinical decision support tool for primary health care management of back pain: Development and mixed methods evaluation**. *Journal of Medical Internet Research* 2014, **16**(4):1.
110. Perreault K, Dionne CE, Rossignol M, Morin D: **Interprofessional practices of physiotherapists working with adults with low back pain in Quebec's private sector: Results of a qualitative study**. *BMC Musculoskeletal Disorders* 2014, **15**(1).
111. Pincus T, Vogel S, Breen A, Foster N, Underwood M: **Persistent back pain - Why do physical therapy clinicians continue treatment? A mixed methods study of chiropractors, osteopaths and physiotherapists**. *European Journal of Pain* 2006, **10**(1):67-76.
112. Roberts C, Adebajo AO, Long S: **Improving the quality of care of musculoskeletal conditions in primary care**. *Rheumatology (Oxford, England)* 2002, **41**(5):503.
113. Rogers A: **Whose autonomy? Which choice? A study of GPs' attitudes towards patient autonomy in the management of low back pain**. *Family practice* 2002, **19**(2):140.
114. Rutledge N, Cantero P, Ruiz J: **Managing chronic pain in low income overweight Latinos: provider perceptions**. *Communicating Nursing Research* 2013, **46**:639-640.
115. Samuels G, Leveille M: **Senior nursing students' clinical judgments in pain management**. *Nurse educator* 2010, **35**(5):220.
116. Sanders T, Foster E, Ong BN: **Perceptions of general practitioners towards the use of a new system for treating back pain: a qualitative interview study**. *BMC medicine* 2011, **9**:49.
117. Sanders T, Foster E, Bishop A, Ong BN: **Biopsychosocial care and the physiotherapy encounter: physiotherapists' accounts of back pain consultations**. *BMC musculoskeletal disorders* 2013, **14**:65.
118. Schers H, Wensing M, Huijsmans Z, van M, Grol R: **Implementation barriers for general practice guidelines on low back pain a qualitative study**. *Spine* 2001, **26**(15):E348-353.
119. Skelton AM, Murphy EA, Murphy RJ, O'Dowd TC: **Patient education for low back pain in general practice**. *Patient education and counseling* 1995, **25**(3):329.
120. Skelton AM, Murphy EA, Murphy RJ, O'Dowd TC: **General practitioner perceptions of low back pain patients**. *Family practice* 1995, **12**(1):44.
121. Skjutar A, Schult M-L, Christensson K, Müllersdorf M: **Indicators of need for occupational therapy in patients with chronic pain: occupational therapists' focus groups**. *Occupational therapy international* 2010, **17**(2):93.
122. Smart K, Doody C: **The clinical reasoning of pain by experienced musculoskeletal physiotherapists**. *Manual therapy* 2007, **12**(1):40.
123. Spiers J: **Expressing and responding to pain and stoicism in home-care nurse-patient interactions**. *Scandinavian Journal of Caring Sciences* 2006, **20**(3):293-302.
124. Steihaug S, Malterud K: **Part process analysis: a qualitative method for studying provider-patient interaction**. *Scandinavian Journal of Public Health* 2003, **31**(2):107-113.
125. Stenner K, Courtenay M: **Benefits of nurse prescribing for patients in pain: nurses' views**. *Journal of Advanced Nursing* 2008, **63**(1):27-36.
126. Stenner K, Courtenay M: **The role of inter-professional relationships and support for nurse prescribing in acute and chronic pain**. *Journal of Advanced Nursing* 2008, **63**(3):276-284.
127. Strand LI, Kvale A, Raheim M, Ljunggren AE: **Do Norwegian manual therapists provide management for patients with acute low back pain in accordance with clinical guidelines?** *Manual Therapy* 2005, **10**(1):38-43.
128. Tellier PP, Belanger E, Rodriguez C, Ware MA, Nancy PN: **Improving undergraduate medical education about pain assessment and management: A qualitative descriptive study of stakeholders' perceptions**. *Pain Research and Management* 2013, **18**(5):259-265.
129. Thomson D: **An ethnographic study of physiotherapists' perceptions of their interactions with patients on a chronic pain unit**. *Physiotherapy theory and practice* 2008, **24**(6):408.

For peer review only

For peer review only - http://bmjopen.bmj.com/site/about/guidelines.xhtml

130. Thunberg KA, Hallberg LRM: **The need for organizational development in pain clinics: A case study**. *Disability and Rehabilitation* 2002, **24**(14):755-762.
131. Undeland M, Malterud K: **Diagnostic interaction: the patient as a source of knowledge?** *Scandinavian journal of primary health care* 2008, **26**(4):222.
132. van WP, Beetsma A, Neels H, Roussel N, Nijs J: **Physical therapists should integrate illness perceptions in their assessment in patients with chronic musculoskeletal pain; a qualitative analysis**. *Manual therapy* 2014, **19**(3):229.
133. Vegni E, Mauri E, Moja EA: **Stories from doctors of patients with pain. A qualitative research on the physicians' perspective**. *Supportive Care in Cancer* 2005, **13**(1):18-25.
134. Wainwright D, Boichat C, McCracken M: **Competing patient and professional agendas in service development**. *Journal of health organization and management* 2014, **28**(6):777.
135. Wermeling M, Scherer M, Himmel W: **GPs' experiences of managing non-specific neck pain - A qualitative study**. *Family Practice* 2011, **28**(3):300-306.
136. Wootton IM, Wood V, Cook F: **Who wants expert patient programmes for chronic mechanical spinal pain?. An investigation into the value of, and recruitment to, an expert patient programme as part of the physiotherapy management of chronic spinal pain**. *Physiotherapy* 2008, **94**(1):78-84.
137. Schofield P: **Talking to older people in care homes: Perceptions of their pain and their preferred management strategies. Results of a pilot study**. *International Journal on Disability and Human Development* 2006, **5**(1):53.
138. Corbett M, Foster N, Ong BN: **GP attitudes and self-reported behaviour in primary care consultations for low back pain**. *Family practice* 2009, **26**(5):359.
139. Crowe M, Whitehead L, Jo M, Baxter D, Panckhurst A: **Self-management and chronic low back pain: a qualitative study**. *Journal of Advanced Nursing* 2010a, **66**(7):1478-1486.
140. Hadker N, Garg S, Chandran B, Crean M, McNett M, Silverman L: **Efficient practices associated with diagnosis, treatment and management of fibromyalgia among primary care physicians**. *Pain research & management : the journal of the Canadian Pain Society = journal de la société canadienne pour le traitement de la douleur* 2011, **16**(6):440.
141. Dysvik E, Stephens P: **Conducting rehabilitation groups for people suffering from chronic pain**. *International journal of nursing practice* 2010, **16**(3):233.
142. Kotarba A, Seidel V: **Managing the problem pain patient: Compliance or social control?** *Social Science & Medicine* 1984, **19**(12):1393.
143. Wilson N, Pope C, Roberts L, Crouch R: **Governing healthcare: Finding meaning in a clinical practice guideline for the management of non-specific low back pain**. *Social Science & Medicine* 2014, **102**:138.
144. Slade SC, Molloy E, Keating JL: **The dilemma of diagnostic uncertainty when treating people with chronic low back pain: a qualitative study**. *Clinical Rehabilitation* 2012, **26**(6):558-570.
145. Wainwright D, Calnan M, O'Neil C, Winterbottom A, Watkins C: **When pain in the arm is 'all in the head': The management of medically unexplained suffering in primary care**. *Health, Risk & Society* 2006, **8**(1):71.
146. Afrell M, Rudebeck CE: **'We got the whole story all at once': physiotherapists' use of key questions when meeting patients with long-standing pain**. *Scandinavian journal of caring sciences* 2010, **24**(2):281.
147. Thunberg KA, Carlsson SG, Hallberg LR: **Health care professionals' understanding of chronic pain: a grounded theory study**. *Scandinavian Journal of Caring Sciences* 2001, **15**(1):99-105.
148. Oien AM, Steihaug S, Iversen S, Raheim M: **Communication as negotiation processes in long-term physiotherapy: a qualitative study**. *Scandinavian Journal of Caring Sciences* 2011, **25**(1):53-62.
149. Macneela P, Gibbons A, McGuire B, Murphy A: **"We need to get you focused": general practitioners' representations of chronic low back pain patients**. *Qualitative Health Research* 2010, **20**(7):977-986.

For peer review only - http://bmjopen.bmj.com/site/about/guidelines.xhtml

150. Gropelli T, Sharer J: **Nurses' Perceptions of Pain Management in Older Adults**. *MEDSURG Nursing* 2013, **22**(6):375-383.
151. Eccleston C, De CACA, Rogers WS: **Patients' and professionals' understandings of the causes of chronic pain: blame, responsibility and identity protection**. *Social Science & Medicine* 1997, **45**(5):699-710.
152. Allegretti A, Borkan J, Reis S, Griffiths F: **Paired interviews of shared experiences around chronic low back pain: classic mismatch between patients and their doctors**. *Family Practice* 2010, **27**(6):676-683.
153. Åsbring P, Närvänen A: **Ideal versus reality: physicians perspectives on patients with chronic fatigue syndrome (CFS) and fibromyalgia**. *Social Science & Medicine* 2003, **57**(4):711-721.
154. Baldacchino A, Gilchrist G, Fleming R, Bannister J: **Guilty until proven innocent: A qualitative study of the management of chronic non-cancer pain among patients with a history of substance abuse**. *Addictive Behaviors* 2010, **35**(3):270-272.
155. Barker KL, Heelas L, Toye F: **Introducing Acceptance and Commitment Therapy to a physiotherapy led pain rehabilitation programme: An Action Research Study**. *British Journal of Pain* 2015.
156. Barry T, Irwin S, Jones S, Becker C, Tetrault M, Sullivan E, Hansen H, O'Connor G, Schottenfeld S, Fiellin A: **Opioids, chronic pain, and addiction in primary care**. *The journal of pain : official journal of the American Pain Society* 2010, **11**(12):1442.
157. Baszanger I: **Deciphering chronic pain**. *Sociology of Health and Illness* 1992, **14**(2):181-215.
158. Berg M, Arnsten H, Sacajiu G, Karasz A: **Providers' experiences treating chronic pain among opioid-dependent drug users**. *Journal of general internal medicine* 2009, **24**(4):482.
159. Bergman A, Matthias S, Coffing M, Krebs E: **Contrasting Tensions Between Patients and PCPs in Chronic Pain Management: A Qualitative Study**. *Pain Medicine* 2013, **14**(11):1689-1698.
160. Blomberg AM, Hylander I, Tornkvist L: **District nurses' involvement in pain care: a theoretical model**. *Journal of Clinical Nursing* 2008, **17**(15):2022-2031.
161. Blomqvist K: **Older people in persistent pain: nursing and paramedical staff perceptions and pain management**. *Journal of Advanced Nursing* 2003, **41**(6):575-585.
162. Briones-Vozmediano E, Vives-Cases C, Ronda-Perez E, Gil-Gonzalez D: **Patients' and professionals' views on managing fibromyalgia**. *Pain Research and Management* 2013, **18**(1):19-24.
163. Cameron A, Smith H, Schofield A: **HEALTH CARE PROFESSIONALS' ACCOUNTS OF CHRONIC PAIN MANAGEMENT FOR OLDER ADULTS**. *Journal of the Physiotherapy Pain Association* 2015(38):17-28.
164. Cartmill C, Soklaridis S, Cassidy J: **Transdisciplinary teamwork: The experience of clinicians at a functional restoration program**. *Journal of occupational rehabilitation* 2011, **21**(1):1.
165. Chew-Graham C, May C: **Chronic low back pain in general practice: The challenge of the consultation**. *Family practice* 1999, **16**(1):46.
166. Clark L, Jones K, Pennington K: **Pain assessment practices with nursing home residents**. *Western Journal of Nursing Research* 2004, **26**(7):733-751.
167. Clark L, Fink R, Pennington K, Jones K: **Nurses' Reflections on Pain Management in a Nursing Home Setting**. *Pain Management Nursing* 2006, **7**(2):71.
168. Côté P, Clarke J, Deguire S, Frank JW, Yassi A: **Chiropractors and return-to-work: the experiences of three Canadian focus groups**. *Journal of manipulative and physiological therapeutics* 2001, **24**(5):309.
169. Coutu MF, Baril R, Durand MJ, Cote D, Cadieux G: **Clinician-patient agreement about the work disability problem of patients having persistent pain: why it matters**. *Journal of Occupational Rehabilitation* 2013, **23**(1):82-92.

For peer review only - http://bmjopen.bmj.com/site/about/guidelines.xhtml

170. Dahan R, Borkan J, Brown JB, Reis S, Hermoni D, Harris S: **The challenge of using the low back pain guidelines: a qualitative research**. *Journal of Evaluation in Clinical Practice* 2007, **13**(4):616-620.
171. Daykin AR, Richardson B: **Physiotherapists' pain beliefs and their influence on the management of patients with low back pain**. *Spine* 2004, **29**(7):783-795.
172. Dobbs D, Baker T, Carrion IV, Vongxaiburana E, Hyer K: **Certified nursing assistants' perspectives of nursing home residents' pain experience: communication patterns, cultural context, and the role of empathy**. *Pain management nursing : official journal of the American Society of Pain Management Nurses* 2014, **15**(1):87-96.
173. Espeland A, Baerheim A: **Factors affecting general practitioners' decisions about plain radiography for back pain: implications for classification of guideline barriers--a qualitative study**. *BMC Health Services Research* 2003, **3**(1).
174. Esquibel AY, Borkan J: **Doctors and patients in pain: Conflict and collaboration in opioid prescription in primary care**. *Pain* 2014, **155**(12):2575-2582.
175. Fontana JS: **The social and political forces affecting prescribing practices for chronic pain**. *Journal of Professional Nursing* 2008, **24**(1):30-36.
176. Fox P, Solomon P, Raina P, Jadad A: **Barriers and facilitators in pain management in long-term care institutions: a qualitative study**. *Canadian Journal on Aging* 2004, **23**(3):269-280.
177. Gooberman-Hill R, Heathcote C, Reid M, Horwood J, Beswick D, Williams S, Ridd J: **Professional experience guides opioid prescribing for chronic joint pain in primary care**. *Family practice* 2011, **28**(1):102.
178. Hansson M, Bostrom C, Harms-Ringdahl K: **Living with spine-related pain in a changing society--a qualitative study**. *Disability & Rehabilitation* 2001, **23**(7):286-295.
179. Harting J, Rutten GM, Rutten ST, Kremers P: **A qualitative application of the diffusion of innovations theory to examine determinants of guideline adherence among physical therapists**. *Physical therapy* 2009, **89**(3):221.
180. Hayes M, Myhal C, Thornton F, Camerlain M, Jamison C, Cytryn N, Murray S: **Fibromyalgia and the therapeutic relationship: Where uncertainty meets attitude**. *Pain Research & Management* 2010, **15**(6):385-391.
181. Hellman T, Jensen I, Bergström G, Busch H: **Returning to work - a long-term process reaching beyond the time frames of multimodal non-specific back pain rehabilitation**. *Disability & Rehabilitation* 2015, **37**(6):499-506.
182. Hellstrom O, Bullington J, Karlsson G, Lindqvist P, Mattsson B: **Doctors' attitudes to fibromyalgia: a phenomenological study**. *Scandinavian Journal of Social Medicine* 1998, **26**(3):232-237.
183. Holloway K, McConigley R: **Descriptive, exploratory study of the role of nursing assistants in Australian residential aged care facilities: The example of pain management**. *Australasian Journal on Ageing* 2009, **28**(2):70-74.
184. Holloway K, McConigley R: **Understanding nursing assistants' experiences of caring for older people in pain: the Australian experience [corrected] [published erratum appears in PAIN MANAGE NURS 2009 Sep;10(3):115]**. *Pain Management Nursing* 2009, **10**(2):99-107.
185. Howarth M, Warne T, Haigh C: **"Let's stick together" – A grounded theory exploration of interprofessional working used to provide person centered chronic back pain services**. *Journal of interprofessional care* 2012, **26**(6):491.
186. Kaasalainen S, Coker E, Dolovich L, Papaioannou A, Hadjistavropoulos T, Emili A, Ploeg J: **Pain management decision making among long-term care physicians and nurses**. *Western journal of nursing research* 2007, **29**(5):561.
187. Kaasalainen S, Martin-Misener R, Carter N, DiCenso A, Donald F, Baxter P: **The nurse practitioner role in pain management in long-term care**. *Journal of Advanced Nursing* 2010, **66**(3):542-552.

For peer review only - http://bmjopen.bmj.com/site/about/guidelines.xhtml

188. Kaasalainen S, Brazil K, Coker E, Ploeg J, Martin-Misener R, Donald F, DiCenso A, Hadjistavropoulos T, Dolovich L, Papaioannou A *et al*: **An action-based approach to improving pain management in long-term care**. *Canadian Journal on Aging* 2010, **29**(4):503-518.
189. Kilaru S, Gadsden M, Perrone J, Paciotti B, Barg K, Meisel F: **How do physicians adopt and apply opioid prescription guidelines in the emergency department? A qualitative study**. *Annals of emergency medicine* 2014, **64**(5):482.
190. Krebs E, Bergman A, Coffing M, Campbell R, Frankel M, Matthias S: **Barriers to guideline-concordant opioid management in primary care—A qualitative study**. *The Journal of Pain* 2014, **15**(11):1148.
191. Kristiansson MH, Brorsson A, Wachtler C, Troein M: **Pain, power and patience--a narrative study of general practitioners' relations with chronic pain patients**. *BMC family practice* 2011, **12**:31.
192. Liu YW: **Exploring nursing assistants' roles in the process of pain management for cognitively impaired nursing home residents: a qualitative study**. *Journal of Advanced Nursing* 2014, **70**(5):1065-1078.
193. Löckenhoff E, Laucks S, Port D, Tung J, Wethington E, Reid MC: **Temporal horizons in pain management: understanding the perspectives of physicians, physical therapists, and their middle-aged and older adult patients**. *The Gerontologist* 2013, **53**(5):850.
194. Lundh C, Segesten K, Björkelund C: **To be a helpless helpoholic--GPs' experiences of women patients with non-specific muscular pain**. *Scandinavian journal of primary health care* 2004, **22**(4):244.
195. McConigley R, Toye C, Goucke R, Kristjanson J: **Developing recommendations for implementing the Australian Pain Society's pain management strategies in residential aged care**. *Australasian journal on ageing* 2008, **27**(1):45.
196. McCrorie C, Closs SJ, House A, Petty D, Ziegler L, Glidewell L, West R, Foy R: **Understanding long-term opioid prescribing for non-cancer pain in primary care: a qualitative study**. *BMC family practice* 2015, **16**:121.
197. Mentes C, Teer J, Cadogan P: **The pain experience of cognitively impaired nursing home residents: perceptions of family members and certified nursing assistants**. *Pain management nursing : official journal of the American Society of Pain Management Nurses* 2004, **5**(3):118.
198. O'Connor BB, Eisenberg DM, Buring JE, Liang CL, Osypiuk K, Levy DB, Wayne PM: **Within-team patterns of communication and referral in multimodal treatment of chronic low back pain patients by an integrative care team**. *Global Advances In Health and Medicine* 2015, **4**(2):36-45.
199. Oosterhof B, Dekker JH, Sloots M, Bartels EA, Dekker J: **Success or failure of chronic pain rehabilitation: the importance of good interaction - a qualitative study under patients and professionals**. *Disability and Rehabilitation* 2014, **36**(22):1903-1910.
200. Parsons S, Harding G, Breen A, Foster N, Pincus T, Vogel S, Underwood M: **Will shared decision making between patients with chronic musculoskeletal pain and physiotherapists, osteopaths and chiropractors improve patient care?** *Family Practice* 2012, **29**(2):203-212.
201. Patel S, Peacock S, McKinley R, Carter DC, Watson PJ: **GPs' experience of managing chronic pain in a South Asian community--a qualitative study of the consultation process**. *Family practice* 2008, **25**(2):71.
202. Patel S, Peacock SM, McKinley RK, Clark-Carter D, Watson PJ: **GPs' perceptions of the service needs of South Asian people with chronic pain: A qualitative enquiry**. *Journal of Health Psychology* 2009, **14**(7):909-918.
203. Paulson M, Danielson E, Norberg A: **Nurses' and physicians' narratives about long-term non-malignant pain among men**. *Journal of Advanced Nursing* 1999, **30**(5):1097-1105.

For peer review only

For peer review only - http://bmjopen.bmj.com/site/about/guidelines.xhtml

204. Poitras S, Durand M-J, Côté A-M, Tousignant M: **Use of low-back pain guidelines by occupational therapists: A qualitative study of barriers and facilitators**. *Work: Journal of Prevention, Assessment & Rehabilitation* 2011, **39**(4):465.

205. Ruiz JG, Qadri SS, Nader S, Wang J, Lawler T, Hagenlocker B, Roos BA: **Primary care management of chronic nonmalignant pain in veterans: a qualitative study**. *Educational Gerontology* 2010, **36**(5):372-394.

206. Schulte E, Heift A, Welke J, Hermann K, Schäfer M, Boemke W: **Specialised pain therapy from the general practitioners' perspective: A qualitative assessment**. *Journal of Pain Management* 2010, **3**(3):263.

207. Scott-Dempster C, Toye F, Truman J, Barker K: **Physiotherapists' experiences of activity pacing with people with chronic musculoskeletal pain. An interpretative phenomenological analysis.** *Physiotherapy Theory & Practice* 2014, **30**(5):319-328.

208. Seamark D, Seamark C, Greaves C, Blake S: **GPs prescribing of strong opioid drugs for patients with chronic non-cancer pain: a qualitative study**. *The British journal of general practice : the journal of the Royal College of General Practitioners* 2013, **63**(617).

209. Shye D, Freeborn DK, Romeo J, Eraker S: **Understanding physicians' imaging test use in low back pain care: the role of focus groups**. *International journal for quality in health care : journal of the International Society for Quality in Health Care / ISQua* 1998, **10**(2):83.

210. Siedlecki L, Modic MB, Bernhofer E, Sorrell J, Strumble P, Kato I: **Exploring how bedside nurses care for patients with chronic pain: a grounded theory study**. *Pain management nursing : official journal of the American Society of Pain Management Nurses* 2014, **15**(3):565.

211. Sloots M, Scheppers EF, Bartels EAC, Dekker JHM, Geertzen JHB, Dekker J: **First rehabilitation consultation in patients of non-native origin: factors that lead to tension in the patient-physician interaction**. *Disability & Rehabilitation* 2009, **31**(22):1853-1862.

212. Sloots M, Dekker JHM, Pont M, Bartels EA, Geertzen JHB, Dekker J: **Reasons for drop-out from rehabilitation in patients of Turkish and Moroccan origin with chronic low back pain in The Netherlands: a qualitative**. *Journal of Rehabilitation Medicine* 2010, **42**(6):566-574.

213. Spitz A, Moore A, Papaleontiou M, Granieri E, Turner J, Reid MC: **Primary care providers' perspective on prescribing opioids to older adults with chronic non-cancer pain: a qualitative study**. *BMC geriatrics* 2011, **11**:35.

214. Starrels L, Wu B, Peyser D, Fox D, Batchelder A, Barg K, Arnsten H, Cunningham O: **It made my life a little easier: primary care providers' beliefs and attitudes about using opioid treatment agreements**. *Journal of opioid management* 2014, **10**(2):95.

215. Stinson J, White M, Isaac L, Campbell F, Brown S, Ruskin D, Gordon A, Galonski M, Pink L, Buckley N *et al*: **Understanding the information and service needs of young adults with chronic pain: perspectives of young adults and their providers**. *The Clinical journal of pain* 2013, **29**(7):600.

216. Toye F, Jenkins S, Seers K, Barker K: **Exploring the value of qualitative research films in clinical education**. *BMC Medical Research Methodology* 2015, **15**(214):http://bmcmededuc.biomedcentral.com/articles/10.1186/s12909-12015-10491-12902.

217. Tveiten S, Meyer I: **'Easier said than done': empowering dialogues with patients at the pain clinic - the health professionals' perspective**. *Journal of Nursing Management* 2009, **17**(7):804-812.

218. Wainwright D, Calnan M, O'Neil C, Winterbottom A, Watkins C: **When pain in the arm is 'all in the head': the management of medically unexplained suffering in primary care**. *Health, Risk & Society* 2006, **8**(1):71-89.

219. Wynne-Jones G, van dD, Ong BN, Bishop A, Cowen J, Artus M, Sanders T: **Perceptions of health professionals towards the management of back pain in the context of work: a qualitative study**. *BMC musculoskeletal disorders* 2014, **15**:210.

For peer review only

220. Zanini C, Sarzi-Puttini P, Atzeni F, Di FM, Rubinelli S: **Doctors' insights into the patient perspective: a qualitative study in the field of chronic pain**. *BioMed Research International* 2014:514230-514231.

221. Engel G: **The need for a new medical model: a challenge for biomedicine** *Science* 1977, **196** 129 - 136

222. Foucault M: **The birth of the clinic**. Abingdon, Oxon, UK: Routledge Classics; 2003.

223. Helman C: **Culture Health and Illness** 4th edn: Butterworth Heinemann. ; 2007.

224. Dowell D, Haegerich TM, Chou R: **CDC (Centers for Disease Control and Prevention) Guideline for Prescribing Opioids for Chronic Pain — United States, 2016**. *Morbidity and Mortality Weekly* 2016, **Report 65, March 15**:1-49.

225. **Opioids Aware: A resource for patients and healthcare professionals to support prescribing of opioid medicines for pain**

226. Chou R, Turner JA, Devine EB, Hansen RN, Sullivan SD, Blazina I, Dana T, Bougatsos C, Deyo RA: **The Effectiveness and Risks of Long-Term Opioid Therapy for Chronic Pain: A Systematic Review for a National Institutes of Health Pathways to Prevention Workshop**. *Annals of Internal Medicine* 2015, **162**(4):1-20 http://annals.org/aim/article/2089370/effectiveness-risks-long-term-opioid-therapy-chronic-pain-systematic-review.

227. Baker SE, Edwards R: **How many qualitative interviews is enough?** *National Centre for Research Methods Review Paper: Expert voices and early career reflections on sampling and cases in qualitative research* 2012, **http://eprints.ncrm.ac.uk/2273/**(last accessed 12th July 2013).

For peer review only

For peer review only - http://bmjopen.bmj.com/site/about/guidelines.xhtml

Figure 1: Flow diagram of records identified and studies removed and included

Figure 2: Line of Argument

For peer review only

For peer review only - http://bmjopen.bmj.com/site/about/guidelines.xhtml

Table 1: Example search syntax for Medline

**(I) QUALITATIVE SUBJECT HEADINGS**	EXP QUALITATIVE RESEARCH<br>EXP INTERVIEWS AS TOPIC<br>EXP FOCUS GROUPS<br>NURSING METHODOLOGY RESEARCH<br>ATTITUDE TO HEALTH
**(II) QUALITATIVE FREE TEXT TERMS**	qualitative adj5 (theor* or study or studies or research or analysis)<br>ethno.ti,ab<br>emic or etic. ti,ab<br>phenomenolog*.ti,ab<br>hermeneutic*.ti,ab<br>heidegger* or husserl* or colaizzi* or giorgi* or glaser or strauss or (van and kaam*) or (van and manen) or ricoeur or spiegelberg* or merleau).ti,ab<br>constant adj3 compar*.ti,ab<br>focus adj3 group*.ti,ab<br>grounded adj3 (theor* or study or studies or research or analysis).ti,ab<br>narrative adj3 analysis.ti,ab<br>discourse adj3 analysis.ti,ab<br>(lived or life) adj3 experience*.ti,ab<br>(theoretical or purposive) adj3 sampl*.ti,ab<br>(field adj note*) or (field adj record*) or fieldnote*.ti,ab<br>participant* adj3 observ*.ti,ab<br>action adj research.ti,ab<br>(digital adj record) or audiorecord* or taperecord* or videorecord* or videotap*).ti,ab<br>(cooperative and inquir*) or (co and operative and inquir*) or (co-operative and inquir*) .ti,ab<br>(semi-structured or semistructured or unstructured or structured) adj3 interview*.ti,ab<br>(informal or in-depth or indepth or "in depth") adj3 interview*.ti,ab<br>(face-to-face" or "face to face") adj3 interview*.ti,ab<br>"ipa" or "interpretative phenomenological analysis".ti,ab<br>"appreciative inquiry".ti,ab<br>(social and construct*) or (postmodern* or post-structural*) or (post structural* or poststructural*) or (post modern*) or post-modern* or feminis*.).ti,ab<br>humanistic or existential or experiential.ti,ab
**(III) PAIN SUBJECT HEADINGS**	EXP BACK PAIN/ OR EXP CHRONIC PAIN/ OR EXP LOW BACK PAIN/ OR EXP MUSCULOSKELETAL PAIN/ OR EXP PAIN/ OR EXP PAIN CLINICS/.<br>EXP FIBROMYALGIA/<br>EXP PAIN MANAGEMENT/
**(IV) PAIN FREE TEXT TERMS**	(chronic* or persistent* or long-stand* or longstand* or unexplain* or un-explain*)<br>fibromyalgia<br>"back ache" or back-ache or backache<br>"pain clinic" or pain-clinic*<br>pain adj5 syndrome*

For peer review only - http://bmjopen.bmj.com/site/about/guidelines.xhtml

Table 2 Author and year of publication, country, data collection method, analytic approach, professional group and context, participants and study focus

AUTHOR/YEAR	COUNTRY	DATA COLLECTION	ANALYTIC APPROACH	ORDER OF ANALYSIS & PROFESSIONAL GROUP/CONTEXT	PARTICIPANTS	STUDY FOCUS
**AFRELL 2010[146]**	NORWAY	FOCUS GROUPS	PHENOMENOLOGY	4. SPECIALIST PHYSIOS	**6 Physiotherapists** with 10-15 years' experience: (3 pain management, 2 primary care, 1 private practise)	To explore physiotherapists experienced prepared key questioning for patients with long-standing pain.
**ALLEGRETTI 2010[152]**	USA	SEMI-STRUCTURED INTERVIEW	IMMERSION-CRYSTALLISATION	1. PRIMARY CARE PHYSICIANS/GPS	**13 Physicians**:<br>5 residents<br>8 attending	To explore shared experiences among chronic LBP patients and their physicians
**ASBRING 2003[153]**	SWEDEN	SEMI-STRUCTURED INTERVIEW	GROUNDED THEORY	5. FIBROMYALGIA	**26 Physicians:** (GP, rheumatology, infectious diseases, rehabilitation, internal medicine and neurology)	To explore: (1) How physicians describe patients with chronic fatigue and fibromyalgia; (2) What the conditions mean to physicians; (3) strategies used.
**BALDACCHINO 2010[154]**	SCOTLAND	FOCUS GROUPS AND INTERVIEWS	FRAMEWORK ANALYSIS	8. OPIOID PRESCRIPTION	**29 Physicians**: (Primary care, addiction specialists, pain specialist, rheumatologist)	To explore physicians' attitudes and experience of prescribing opioids for chronic non-cancer pain with a history of substance abuse
**BARKER 2015[155]**	UK	SEMI-STRUCTURED INTERVIEWS	ACTION RESEARCH	4. SPECIALIST PHYSIOS	**7 Physiotherapists**: (1 clinical lead, 3 advanced practitioners, 2 senior physiotherapists, I assistant practitioner)	To explore the implementation of Acceptance and Commitment Therapy to physiotherapy led pain rehabilitation programme.
**BARRY 2010[156]**	USA	SEMI-STRUCTURED INTERVIEW	GROUNDED THEORY	8. OPIOID PRESCRIPTION	**23 physicians**	To explore physicians' attitudes and experiences about treating chronic non-cancer pain.
**BASZANGER 1992[157]**	FRANCE	ETHNOGRAPHY	GROUNDED THEORY	6. CHRONIC PAIN SERVICES	**NK Physicians**: 326 consultations of pain medicine specialists observed	To explore how physicians specialising in pain medicine work at deciphering chronic pain
**BERG 2009[158]**	USA	SEMI-STRUCTURED	THEMATIC ANALYSIS	8. OPIOID PRESCRIPTION	**12 Physicians**<br>**4 'Physician assistants'**	To explore providers' perceptions of ambiguity, and examine strategies for making diagnostic

For peer review only - http://bmjopen.bmj.com/site/about/guidelines.xhtml

		INTERVIEW				and treatment decisions to manage chronic pain (methadone maintenance therapy)
**BERGMAN 2013[159]**	USA	INTERVIEWS	THEMATIC ANALYSIS	1. PRIMARY CARE PHYSICIANS/GPS	**14 GPs**	To explore the experiences of patients and HCPs communicating with each other about pain management in the primary care setting
**BLOMBERG 2008[160]**	SWEDEN	FOCUS GROUPS	GROUNDED THEORY	11. NURSING	**20 district nurses** (10 with pain management training)	To explore and explain district nurses care of chronic pain sufferers
**BLOMQVIST 2003[161]**	SWEDEN	INTERVIEWS	CONTENT ANALYSIS	9. OLDER ADULTS	**52 mixed HCPs** (35 Auxiliary Nurses , 13 Registered nurses, 4 PT/OTs	To explore HCPs perceptions of older people in persistent pain
**BRIONES-VOZMEDIANO 2013[162]**	SPAIN	SEMI-STRUCTURED INTERVIEW	DISCOURSE ANALYSIS	5. FIBROMYALGIA	**9 mixed HCPs**: (GPs, occupational health physicians, physiotherapists, rheumatologists, psychologists, psychiatrist)	To explore experiences of fibromyalgia management, diagnostic approach, therapeutic management and the health professional-patient relationship
**CAMERON 2015[163]**	SCOTLAND	SEMI-STRUCTURED TELEPHONE INTERVIEWS	THEMATIC ANALYSIS	9. OLDER ADULTS	**13 mixed HCPs**: (GPs, Anaesthetist, Elderly care physician, OT/PT, Nurse, Psychologist)	To explore attitudes and approaches to pain management of older adults, from the perspectives of HCPs' representing multidisciplinary teams
**CARTMILL 2011[164]**	CANADA	SEMI-STRUCTURED INTERVIEW	GROUNDED THEORY	6. CHRONIC PAIN SERVICES	**10 mixed HCPs**: (OT/PT, kinesiology, cognitive behavioural, psychology, work, resource specialty, customer service)	To explore the experience of transition from an interdisciplinary team to a transdisciplinary model of care in a functional restoration program (FRP) for chronic MSK pain.
**CHEW-GRAHAM 1999[165]**	UK	SEMI-STRUCTURED INTERVIEW	GROUNDED THEORY	1. PRIMARY CARE PHYSICIANS/GPS	**20 GPs**	To explore how GPs understand chronic low back pain, how they approach the consultation and how they conceptualize the management of this problem.
**CLARK 2004[166]**	USA	FOCUS GROUPS	ETHNOGRAPHY	10. PAIN IN AGE CARE FACILITES	**NK Mixed:** (licensed and unlicensed care nurses and other worker)	To explore the kinds of pain assessments nursing home staff use with nursing home residents and the characteristics and behaviours of residents that staff consider as they assess pain.
**CLARK 2006[167]**	USA	SEMI-STRUCTURED INTERVIEWS	CONTENT ANALYSIS	10. PAIN IN AGE CARE FACILITES	**103 mixed HCPs:** (9 administrators, 38 registered nurses, 26 licensed practical	To explore the perceptions of a nursing home staff who participated in a study to develop and evaluate a multifaceted pain-management

					nurses, 22 certified nursing assistants, 2 rehab therapists, 3 social workers, 3 directors/assistants)	intervention.
**COTE 2001[168]**	CANADA	FOCUS GROUPS	THEMATIC ANALYSIS	7. PAIN RELATED WORK DISABILITY	**30 chiropractors** (involved in return to work)	To explore concept of timely return-to-work with musculoskeletal injuries, approaches to treatment of injured workers and perspectives on the barriers and facilitators of successful return-to-work.
**COUTU 2013[169]**	CANADA	SEMI-STRUCTURED INTERVIEWS	THEMATIC ANALYSIS	7. PAIN RELATED WORK DISABILITY	**5 occupational therapy/kinesiology**	To explore differences between clinical judgment, workers' representations about their disability and clinicians' interpretations of these representations.
**DAHAN 2007[170]**	ISRAEL	FOCUS GROUPS	IMMERSION-CRYSTALLIZATION	9. GUIDELINES	**38 GPs**	To explore barriers and facilitators for the implementation of low back pain guidelines from GPs perspective.
**DAYKIN 2004[171]**	UK	SEMI-STRUCTURED INTERVIEWS	GROUNDED THEORY	3. PHYSIOTHERAPISTS	**6 Physiotherapists**	To explore physiotherapists' pain beliefs and the role they played within their management of chronic low back pain.
**DOBBS 2014[172]**	USA	FOCUS GROUPS	CONTENT ANALYSIS	10. PAIN IN AGE CARE FACILITES	**28 nursing assistants**	To explore: 1) communication about pain between nursing home residents and nursing assistants; 2) how race and ethnicity influence experiences; and 3) assistants' pain experiences that affect their empathy.
**ECCLESTON 1997[151]**	UK	Q-ANALYSIS	Q-ANALYSIS	2. MIXED HEALTHCARE PROFESSIONALS	**11 mixed HCPs:** (5 anaesthetists, 4 psychologists, 1 nurse, 1 physiotherapist)	To explore how sense is made of the causes of chronic pain
**ESPELAND 2003[173]**	NORWAY	FOCUS GROUPS	PHENOMENOLOGY	9. GUIDELINES	**13 GPs**	To explore: A) that affect GPs decisions about ordering x-rays for back pain and B) barriers to guideline adherence.
**ESQUIBEL 2014[174]**	USA	INTERVIEWS	IMMERSION-CRYSTALLIZATION	8. OPIOID PRESCRIPTION	**21 Family practitioners** (10 residents, 6 attending)	To explore the experiences of adults receiving opioid therapy for relief of chronic non-cancer pain and that of their physicians
**FONTANA 2008[175]**	USA	SEMI-STRUCTURED	EMANCIPATORY RESEARCH	8. OPIOID PRESCRIPTION	**9 Advanced practice nurses**	To explore factors that influences the prescribing practices of advanced practice

For peer review only - http://bmjopen.bmj.com/site/about/guidelines.xhtml

		INTERVIEW				nurses for patients with chronic non-malignant pain.
**FOX 2004[176]**	CANADA	FOCUS GROUPS	THEMATIC ANALYSIS	10. PAIN IN AGE CARE FACILITES	**54 Mixed HCPs:** (13 health care aides, 8 registered practice nurses, 19 registered nurses, 6 physicians, 8 OT/PT)	To explore barriers to the management of pain in long-term care institutions.
**GOOBERMAN-HILL 2011[177]**	UK	SEMI-STRUCTURED INTERVIEW	THEMATIC ANALYSIS	8. OPIOID PRESCRIPTION	**27 GPs**	To explore GPs' opinions about opioids and decision-making processes when prescribing 'strong' opioids for chronic joint pain.
**GROPELLI 2013[150]**	USA	SEMI-STRUCTURED INTERVIEWS	CONTENT ANALYSIS	10. PAIN IN AGE CARE FACILITES	**16 Nurses** (registered and licensed practical nurses)	To explore nurses' perceptions of pain management in older adults in long-term care.
**HANSSON 2001[178]**	SWEDEN	INTERVIEWS	GROUNDED THEORY	7. PAIN RELATED WORK DISABILITY	**4 physicians**	To explore life lived with recurrent, spine-related pain and to explore the development from work to disability pension.
**HARTING 2009[179]**	NETHERLANDS	FOCUS GROUPS	CONTENT ANALYSIS	9. GUIDELINES	**30 physiotherapists**	To explore the determinants of guideline adherence among physical therapists
**HAYES 2010[180]**	CANADA	FOCUS GROUPS AND INTERVIEWS	GROUNDED THEORY	5. FIBROMYALGIA	**32 Physicians**: (GPs, rheumatologists, psychiatrists, neurologists, anaesthesiologists)	To explore knowledge and attitudinal challenges affecting optimal care in fibromyalgia
**HELLMAN 2015[181]**	SWEDEN	SEMI-STRUCTURED INTERVIEWS	THEMATIC ANALYSIS	7. PAIN RELATED WORK DISABILITY	**15 Mixed HCPs:** (4 OTs, 4 PTs, 2 social workers, 3 physicians, 2 psychologists)	To explore health professionals' experience of working with return to work (RTW) in multimodal rehabilitation for people with non-specific back pain.
**HELLSTROM 1998[182]**	SWEDEN	INTERVIEWS	PHENOMENOLOGY	5. FIBROMYALGIA	**20 Physicians:** (10 rheumatologists, 10 GPs)	To explore the clinical experiences of doctors when meeting patients with fibromyalgia
**HOLLOWAY 2009A[183]**	AUSTRALIA	SEMI-STRUCTURED INTERVIEWS	CONSTANT COMPARISON	10. PAIN IN AGE CARE FACILITES	**6 Nursing assistants**	To explore the experiences of nursing assistants who work with older people in residential aged care facilities (chronic pain example)
**HOLLOWAY 2009B#[184]**	AUSTRALIA	SEMI-STRUCTURED INTERVIEWS	CONSTANT COMPARISON	10. PAIN IN AGE CARE FACILITES	**6 Nursing assistants**	To explore the experiences of nursing assistants who work with older people in residential aged care facilities (chronic pain example)

For peer review only - http://bmjopen.bmj.com/site/about/guidelines.xhtml

**HOWARTH 2012[185]**	UK	INTERVIEWS AND FOCUS GROUPS	GROUNDED THEORY	6. CHRONIC PAIN SERVICES	**9 mixed HCPs:** (Consultant nurse, physiotherapist, 2 consultant psychologists, 2 pain nurses, 3 anaesthetists)	To explore person-centred care from the perspectives of people with chronic back pain and the inter-professional teams who care for them.
**KAASALAINEN 2007[186]**	CANADA	INTERVIEWS AND 8 FOCUS GROUPS	GROUNDED THEORY	10. PAIN IN AGE CARE FACILITES	**66 mixed HCPs:** (Physicians (n= 9), registered practical nurses)	To explore the decision-making process of pain management of physicians/nurses and how their attitudes about pain affect their decisions about prescribing among older adults in long-term care
**KAASALAINEN 2010A[187]**	CANADA	INTERVIEWS AND FOCUS GROUPS	THEMATIC ANALYSIS	10. PAIN IN AGE CARE FACILITES	**NK Mixed HCPs:** (Registered nurses, Personal support workers, nurse practitioners, physicians, pharmacist, PTs, clinical nurse specialists)	To explore the perceptions of healthcare team members who provide care for residents and nurse managers views regarding the nurse practitioner role in pain management in long term care.
**KAASALAINEN 2010B[188]**	CANADA	INTERVIEWS AND FOCUS GROUPS	CASE-STUDY ANALYSIS (YIN)	10. PAIN IN AGE CARE FACILITES	**53 Mixed HCPs:** (15 Registered nurses, 6 registered practical nurses, 4 physicians, 20 unlicensed care practitioners, 2 pharmacists, 2 PTs, 4 administrators)	To explore barriers to pain management in long-term care and develop an inter-professional approach to improve pain management.
**KILARU 2014[189]**	USA	SEMI-STRUCTURED INTERVIEW	GROUNDED THEORY	8. OPIOID PRESCRIPTION	**61 emergency physicians**	To explore themes regarding emergency physicians' definition, awareness, use, and opinions of opioid prescribing guidelines.
**KREBS 2014[190]**	USA	SEMI-STRUCTURED INTERVIEW	IMMERSION-CRYSTALLISATION	8. OPIOID PRESCRIPTION	**14 primary care physicians**	To explore physicians' and patients' perspectives on recommended opioid management practices and to identify potential barriers /facilitators of guideline-concordant opioid management in primary care.
**KRISTIANSSON 2011[191]**	SWEDEN	INTERVIEWS	NARRATIVE ANALYSIS	1. PRIMARY CARE PHYSICIANS/GPS	**5 GPs**	To explore GPs' experience in contact with chronic pain patients and what works and does not work in these consultations.
**LIU 2014[192]**	HONG KONG	INTERVIEWS AND FOCUS GROUPS	CONTENT ANALYSIS	10. PAIN IN AGE CARE FACILITES	**49 Nursing assistants**	To explore nursing assistants roles during the process of pain management for residents

For peer review only - http://bmjopen.bmj.com/site/about/guidelines.xhtml

**LOCKENHOFF 2013[193]**	USA	FOCUS GROUPS	CONTENT ANALYSIS	2. MIXED HEALTHCARE PROFESSIONALS	**44 Mixed HCPs:** 21 physicians, 23 physical therapists	To explore how perceptions of chronological time influence the management of chronic non cancer pain in middle aged and older patients
**LUNDH 2004[194]**	SWEDEN	FOCUS GROUPS	CONSTANT COMPARISON	1. PRIMARY CARE PHYSICIANS/GPS	**14 GPs**	To explore what it means to be a GP meeting patients with non-specific muscular pain
**MACNEELA 2010[149]**	IRELAND	CRITICAL INCIDENT INTERVIEW	THEMATIC ANALYSIS	1. PRIMARY CARE PHYSICIANS/GPS	**12 GPs**	To explore how GPs represent chronic low back pain especially in relation to psychosocial care
**MCCONIGLEY 2008[195]**	AUSTRALIA	INTERVIEWS AND FOCUS GROUPS	THEMATIC ANALYSIS	10. PAIN IN AGE CARE FACILITES	**34 Mixed HCPs:** (7 GPs, 11 Registered nurses, 4 enrolled nurses, 8 allied health professionals, 4 facility managers)	To develop recommendations and a 'toolkit' to facilitate implementation of pain management strategies in Australian Residential Aged Care Facilities
**MCCRORIE 2015[196]**	UK	FOCUS GROUPS	GROUNDED THEORY	8. OPIOID PRESCRIPTION	**15 GPs**	To explore the processes which bring about and perpetuate long-term prescribing of opioids for chronic, non-cancer pain
**MENTES 2004[197]**	USA	SEMI-STRUCTURED INTERVIEWS	THEMATIC ANALYSIS	10. PAIN IN AGE CARE FACILITES	**11 Certified nursing assistants**	To evaluate pain information from formal direct caregivers who cared for cognitively impaired residents
**OCONNOR 2015[198]**	USA	ETHNOGRAPHY	CONSTANT COMPARISON	6. CHRONIC PAIN SERVICES	**NK Mixed HCPs** (psychiatrist, chiropractor acupuncturist, yoga /massage/ craniosacral/movement/ massage/ occupational therapists, medical director, health coach, nutritionist)	To explore patterns of communication and decision making amongst clinicians collaborating in the care of challenging patients with chronic low back pain
**OIEN 2011[148]**	NORWAY	INTERVIEWS, FOCUS GROUPS, OBSERVATION	CASE STUDY (YIN)	3. PHYSIOTHERAPISTS	**6 physiotherapists**	To explore communicative patterns about change in demanding physiotherapy treatment situations.
**OOSTERHOF 2014[199]**	NETHERLANDS	INTERVIEWS AND OBSERVATION	THEMATIC ANALYSIS	6. CHRONIC PAIN SERVICES	**10 mixed HCPs:** (3 OTs, 1 rehabilitation physician, 3 physiotherapists, 2 psychologist, 1 social worker)	To explore which factors are associated with a successful treatment outcome in chronic pain patients and professionals participating in a multidisciplinary rehabilitation program

For peer review only - http://bmjopen.bmj.com/site/about/guidelines.xhtml

**PARSONS 2012[200]**	UK	SEMI-STRUCTURED INTERVIEWS	FRAMEWORK ANALYSIS	2. MIXED HEALTHCARE PROFESSIONALS	**19 mixed HCPs:** (5 osteopaths, 4 chiropractors, 10 physiotherapists)	To explore beliefs about chronic muscular pain and its treatment and how these beliefs influenced care seeking and process of care.
**PATEL 2008[201]**	UK	SEMI-STRUCTURED INTERVIEW	THEMATIC ANALYSIS	1. PRIMARY CARE PHYSICIANS/GPS	**18 GPs** (11South Asian/ 7 white British)	To explore GPs' experiences of managing patients with chronic pain from a South Asian community
**PATEL 2009#[202]**	UK	SEMI-STRUCTURED INTERVIEW	THEMATIC ANALYSIS	1. PRIMARY CARE PHYSICIANS/GPS	**18 GPs** (11South Asian/ 7 white British)	To explore the experiences and needs for management of people from a South Asian community who have chronic pain.
**PAULSON 1999 [203]**	SWEDEN	INTERIEWS	PHENOMENOLOGY	5. FIBROMYALGIA	**21 Mixed HCPs**: (17 nurses, 4 physicians)	To explore the experiences of nurses and physicians in their encounter with men with fibromyalgia
**POITRAS 2011[204]**	CANADA	SEMI-STRUCTURED INTERVIEWS	THEMATIC ANALYSIS	9. GUIDELINES	**9 occupational therapists**	To Identify barriers and facilitators related to the use of low-back pain guidelines as perceived by occupational therapists (OTs).
**RUIZ 2010[205]**	USA	FOCUS GROUPS AND INTERVIEWS	GROUNDED THEORY	9. OLDER ADULTS	**19 mixed HCPs:** (14 primary care physicians, 5 nurse practitioners)	To explore the attitudes of primary care clinicians toward chronic non-malignant pain management in older people
**SCHULTE 2010[206]**	GERMANY	SEMI-STRUCTURED INTERVIEW	THEMATIC ANALYSIS	1. PRIMARY CARE PHYSICIANS/GPS	**10 GPs**	To explore the factors that influence whether referrals from GPs are made, and at what stage, to specialised pain centres.
**SCOTT-DEMPSTER 2014[207]**	UK	SEMI-STRUCTURED INTERVIEWS	IPA*	4. SPECIALIST PHYSIOS	**6 Physiotherapists**	To explore physiotherapists' experiences of using activity pacing with people with chronic musculoskeletal pain.
**SEAMARK 2013[208]**	UK	INTERVIEWS AND FOCUS GROUPS	THEMATIC ANALYSIS	8. OPIOID PRESCRIPTION	**22 GPs**	To explore factors influencing GPs' prescribing of strong opioid drugs for chronic non-malignant pain
**SHYE 1998[209]**	USA	FOCUS GROUPS	IMMERSION-CRYSTALLIZATION	9. GUIDELINES	**22 primary care physicians**	To explore why an intervention to reduce variability in imaging rates for low back pain was ineffective among physicians
**SIEDLECKI 2014[210]**	USA	INTERVIEWS	GROUNDED THEORY	11. NURSING	**48 Nurses**	To explore nurses' assessment and decision-making behaviours related to the care of patients with chronic pain in the acute care setting.
**SLADE 2012[144]**	AUSTRALIA	FOCUS GROUPS	GROUNDED THEORY	3. PHYSIOTHERAPIS	**23 Physiotherapists**	To explore how physiotherapists prescribe exercise for people with non-specific chronic low

For peer review only - http://bmjopen.bmj.com/site/about/guidelines.xhtml

				TS		back pain in the absence of definitive or differential diagnoses.
**SLOOTS 2009[211]**	NETHERLANDS	SEMI-STRUCTURED INTERVIEWS	THEMATIC ANALYSIS	6. CHRONIC PAIN SERVICES	**4 Rehabilitation physicians**	To explore factors lead to tension in the patient–physician interaction in the first consultation by rehabilitation physicians of patients with chronic non-specific low back pain of Turkish and Moroccan origin.
**SLOOTS 2010[212]**	NETHERLANDS	SEMI-STRUCTURED INTERVIEWS	THEMATIC ANALYSIS	6. CHRONIC PAIN SERVICES	**10 mixed HCPs:** (8 Rehabilitation physicians, 1 PT, 1 OT)	To explore which factors led to drop-out in patients of Turkish and Moroccan origin with chronic nonspecific low back pain in a rehabilitation programme.
**SPITZ 2011[213]**	USA	FOCUS GROUPS	THEMATIC ANALYIS	8. OPIOID PRESCRIPTION	**26 Mixed HCPs:** (23 physicians, 3 nurse practitioners)	To explore primary care providers' experiences and attitudes towards prescribing opioids as a treatment for chronic pain among older adults.
**STARRELS 2014[214]**	USA	TELEPHONE INTERVIEW	GROUNDED THEORY	8. OPIOID PRESCRIPTION	**28 physicians**	To explore primary care providers' experiences, beliefs and attitudes about using opioid treatment agreements for patients with chronic pain.
**STINSON 2013[215]**	CANADA	FOCUS GROUPS	THEMATIC ANALYSIS	6. CHRONIC PAIN SERVICES	**17 mixed HCPs:** (6 Physicians, 4 registered nurses, 3 Physiotherapists, 1 Pharmacist, 1 Chiropractor, 1 marriage and family therapist, 1 OT)	To explore the information and service needs of young adults with chronic pain to inform the development of a web-based chronic pain self-management program.
**THUNBERG 2001[147]**	SWEDEN	INTERVIEWS	GROUNDED THEORY	6. CHRONIC PAIN SERVICES	**22 Mixed HCPs:** 7 physicians, 3 psychologist, 2 physios, 8 nurses, 2 social workers	To explore the way healthcare professionals perceive chronic pain
**TOYE 2015[216]**	UK	FOCUS GROUPS	GROUNDED THEORY	2. MIXED HEALTHCARE PROFESSIONALS	**19 mixed HCPs:** 11 GPs, 3 nurses, 3 pharmacists, 1 physiotherapist, 1 psychiatrist)	To explore the impact on healthcare professionals of watching and discussing a short film about patients experience of chronic MSK pain
**TVEITEN 2009[217]**	NORWAY	FOCUS GROUPS	CONTENT ANALYSIS	6. CHRONIC PAIN SERVICES	**5 Mixed HCPs:** (Medicine, Nursing Physiotherapy)	To explore the dialogue between the health professionals and the patient at a pain clinic

For peer review only - http://bmjopen.bmj.com/site/about/guidelines.xhtml

**WAINWRIGHT 2006[218]**	UK	INTERVIEWS	THEMATIC ANALYSIS	1. PRIMARY CARE PHYSICIANS/GPS	**14 GPs**	To explore the dilemma of treating medically explained upper limb disorders
**WILSON 2014[143]**	UK	INTERVIEWS, LETTERS, DOCUMENTS	ETHNOGRAPHY	9. GUIDELINES	**NK Mixed HCPs** (involved in the debate)	To explore the meaning of the guideline and the socio-political events associated with it.
**WYNNE-JONES 2014[219]**	UK	SEMI-STRUCTURED INTERVIEWS	CONSTANT COMPARISON	7. PAIN RELATED WORK DISABILITY	**17 Mixed HCPs:** (11 GP, 6 physios)	To explore GPs' and physiotherapists' perceptions of sickness certification in patients with musculoskeletal problems.
**ZANINI 2014[220]**	ITALY	SEMI-STRUCTURED INTERVIEWS	THEMATIC ANALYSIS	6. CHRONIC PAIN SERVICES	**17 Physicians** (12 rheumatology, 2 neurology, 1 immunology, 1 psychiatry, 1 'nervous and mental disease')	To explore aspects that are important to address during a consultation to build a partnership with patients with chronic pain

SAMPLE REPORTED IN TWO PAPERS
*INTERPRETIVE PHENOMENOLOGICAL ANALYSIS

For peer review only - http://bmjopen.bmj.com/site/about/guidelines.xhtml

Table 3: Confidence in review findings – GRADE-CERQual assessment

REVIEW FINDING	**ADEQUACY NUMBER OF CONCEPTS**	**COHERENCE* NUMBER OF STUDIES/77**	METHODOLOGICAL LIMITATIONS **n = satisfactory (n= key)**	RELEVANCE	OVERALL ASSESSMENT OF CONFIDENCE
SCEPTICAL CULTURAL LENS	43	29 [147, 149, 151, 153, 156, 159, 161, 166-168, 170-174, 180, 182, 183, 188, 190, 191, 194, 196, 200, 210, 213-216]	29 (0)	22 direct, 4 indirect, 2 partial, 1 uncertain	MODERATE
NAVIGATING JUXTAPOSED MODELS OF MEDICINE	77	44 [144, 146-149, 152, 153, 155-158, 162, 165, 168-170, 173, 174, 182, 191, 193, 194, 196, 199, 204, 206, 207, 209, 210, 212, 215-220]	42 (2)	37 direct, 4 indirect, 2 partial, 1 uncertain	HIGH
NAVIGATING TTHE GEOGRAPHY BETWEEN PATIENT AND HCP	92	36 [144, 146-149, 152, 153, 155-158, 162, 165, 168-170, 173, 174, 182, 191, 193, 194, 196, 199, 204, 206, 207, 209, 210, 212, 215-220]	34 (2)	29 direct, 3 indirect, 3 partial, 1 uncertain	MODERATE
THE CRAFT OF PAIN MANAGEMENT	60	31 [143, 144, 149, 150, 153-156, 158, 159, 168, 170-173, 175-177, 179, 185, 188, 194, 195, 199, 200, 204, 205, 208, 210, 213, 219]	29 (2)	27 direct, 2 indirect, 1 partial, 1 uncertain	MODERATE
CHALLENGE OF DUAL ADVOCACY	70	36 [144, 147, 149, 150, 156, 159, 160, 162-164, 166-168, 170, 176, 178, 181, 183-186, 188, 191, 192, 195-198, 202, 204-206, 209, 210, 215, 219]	35 (1)	26 direct, 4 indirect, 5 partial, 1 uncertain	MODERATE
PERSONAL COST	71	33 [146, 148, 152, 153, 155, 156, 158, 159, 161, 162, 165, 166, 170-172, 176, 182-184, 186, 191, 192, 194, 196, 197, 201, 207, 210, 215-217, 219, 220]	32 (1)	28 direct, 4 indirect, 1 partial	MODERATE

*15/371 concepts did not fit conceptual categories

For peer review only - http://bmjopen.bmj.com/site/about/guidelines.xhtml

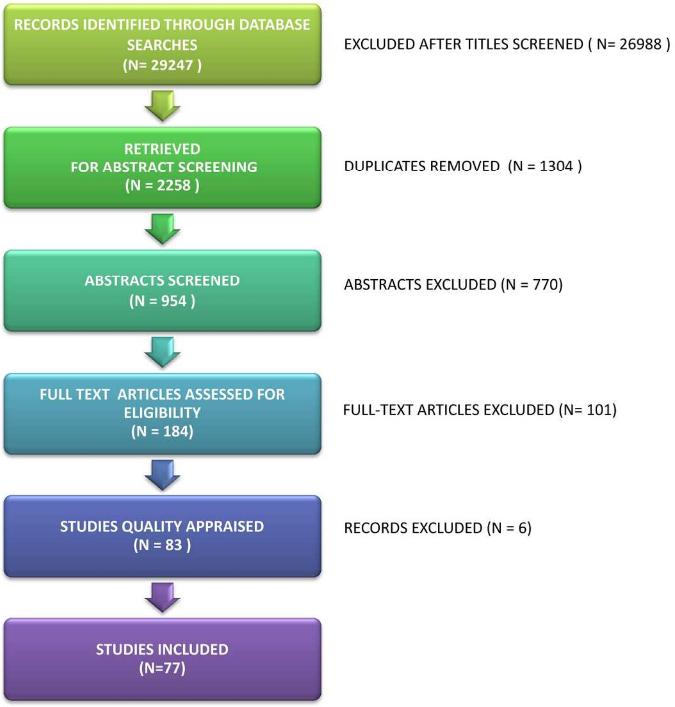

190x142mm (300 x 300 DPI)

ew only

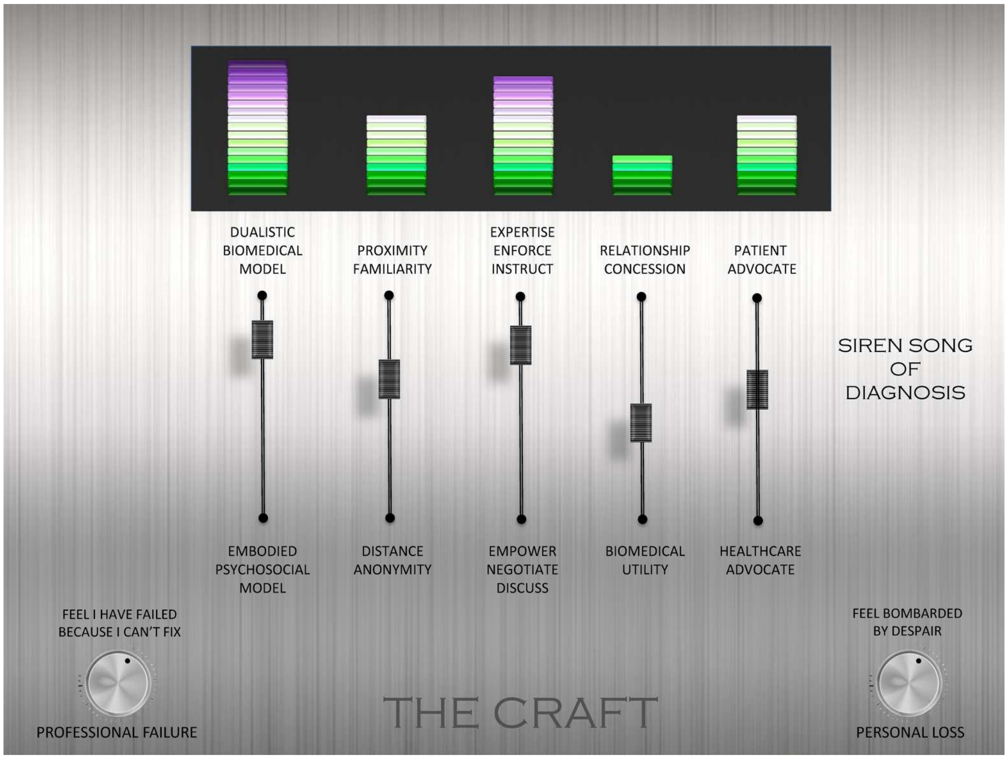

190x142mm (300 x 300 DPI)

For peer review only - http://bmjopen.bmj.com/site/about/guidelines.xhtml

Appendix 1: Concepts not fitting the conceptual analysis

1. **BARRY 2010: Logistical Factors - Ancillary Staff**: Physicians expressed concern that they had insufficient qualified staff to implement pain management.
2. **BARRY 2010: Logistical Factors - Insurance Coverage:** Some physicians expressed concerns about the logistics of insurance coverage for pain management services and the difficulty in characterizing patients' pain status because of restrictions from insurance companies.
3. **FONTANA 2008: critical analysis:** A conflict of interest in which the patients' best interests are given a low priority. Nurses did not see prescribing decisions as ethical ones and, as a result, did not recognize the conflicts that were at work when they made these decisions.
4. **HOLLOWAY 2009A: Initiating clinical care**: The ability to provide pain management for residents when needed varied considerably between facilities; for some it involved basic care such as emotional support, positioning and using hot-packs, whereas in some facilities, they administered pain medication and had responsibility for monitoring the effectiveness of the pain management interventions and documentation.
5. **HOLLOWAY 2009B: Perfect Positioning (Rewards of Getting It Right):** Assistants felt sustained and fulfilled by the rewarding aspects of caring. All spoke of their passion for, enjoyment of, and love for their work (and this is why they stayed in it). Despite the emotional distress associated with observing people in pain, assistants gained satisfaction from seeing residents relieved of pain. Discussed poor financial remuneration they received and expressed the view that it was emotional fulfilment that made the job worthwhile.
6. **KAASALAINEN 2010A: interactions with long-term care staff and managers:** Nurse Practitioner was viewed as a nurse with added skills who assisted other healthcare team members with managing uncontrolled pain and was often used as an additional resource for nurses.
7. **LIU 2014: Instigator implementing non-pharmacological interventions**: Skills in distraction, reassurance and being gentle. Nursing assistants explained how they distracted or reassured residents who were in pain.
8. **LOCKENHOFF 2013: Age Differences in Time Horizons (treatment planning):** Consistently reported that they planned and administered pain management regimens for the long run.
9. **LUNDH 2004: variation 1:** ''I can feel very curious! What do these symptoms stand for?''
10. **OOSTERHOF 2014: Experiences concerning the treatment outcome (Learning new behaviour):** HCPs recognised that behaviour change takes a lot of effort, and requires a combination of explanation and practice. Some patients managed to learn new behaviour

For peer review only

and implement it within their daily life because they have always been active or because of their good body awareness or physical preference. Other might find it difficult to keep up effort due to personal problems and poor social support.

11. **SCOTT-DEMPSTER 2015: ''It's not a One Trick Pony'':** Physiotherapists regarded activity pacing as part of the pain management tool box to bring about change. Activity pacing was not described as something that was clearly definable or had fixed parameters. Achieving this flexibility could be challenging, as it meant that the physiotherapist had to adapt activity pacing for each individual.
12. **SEAMARK 2013: Cost:** Some did not consider cost and prescribed what was needed. Others felt it was important to bear in mind.
13. **SIEDLECKI 2014: CORE CONCEPTS/ TAKING OWNERSHIP:** Some did not take ownership of the problem and saw it as someone else's problem.
14. **STINSON 2013: Barriers to Care (patient-specific barriers):** Difficult to maintain a consistent pain management regimen because of time commitments and reluctance of younger people with pain.
15. **STINSON 2013: Pain Management Strategies (support systems)**: HCPs recognised the importance of peer support for patients.

For peer review only
